# Supplementary material for: Goal management training and psychoeducation / mindfulness for treatment of executive dysfunction in Parkinson’s disease: A feasibility pilot trial
Source: PLoS One. 2022 Feb 18;17(2):e0263108. doi: 10.1371/journal.pone.0263108 (PMC8856541; doi:10.1371/journal.pone.0263108)
Supplement: S1 File — (DOCX) [file pone.0263108.s002.docx]

**Ariane GIGUÈRE-RANCOURT**

"Goal Management Training": Study on the effectiveness of an executive function training program in patients with idiopathic Parkinson's disease

Thesis project presented to

Laval University's School of Psychology

To obtain the rank of

PhD in Psychology, Research and Intervention (Neuropsychology)

*Philosophiæ doctor* (Ph.D.)

Faculty of Social Sciences

Université Laval

June 2017

Quebec, Canada

© Ariane Giguère-Rancourt, 2017

**List of abbreviations**

**ACh** Acetylcholine

**AChE** Acetylcholinesterase

**ACT** *Adaptive Control of Thought*

**AIVQ** Instrumental activities of daily life

**ADL** Activities of Daily Living

**BADS** *Behavioral Assessment of Dysexecutive Syndrome*

**BChE** Butyrilcholinesterase

**BDNF** *Brain Derived Neurotrophic Factor*

**CLES** *Common Language Effect Size*

**COMT** Cathecol-O-Methylransferase

**CVLT** *California Verbal Learning Test*

**DA** Dopamine

**D-KEFS** *Delis and Kaplan Executive Function System*

**DRS-2** *Dementia Rating Scale – 2*

**FDA** *Food and Drug Administration*

**GMT** *Goal Management Training*

**IChE** Cholinesterase Inhibitor

**MAOIS-B** Monoamine Oxidase B Inhibitor

fMRI Functional Magnetic Resonance Imaging

**L-DOPA** Levodopa

**LRRK2** *Leucine-Rich Repeat Kinase 2*

**AD** Alzheimer's disease

**MMSE** *Mini Mental State Examination*

**MoCA** *Montreal Cognitive Assessment*

**MP** Parkinson's disease

**MP-TCL** Parkinson's disease patients with mild cognitive impairment

**MPTP** 1-methyl-4-phenyl-1,2,3,6-tetrahydropyridine

**NINDS** *National Institute of Neurological Disorders and Stroke*

**NMDA** N-Methyl-D-Aspartate

**NPI-12** *NeuroPsychiatric Inventory – 12 items*

**PDQ-39** *Parkinson Disease Questionnaire – 39 items*

**PINK-1** *PTEN-Induced Putative Kinase 1*

**RL/RI-16** Free Recall – 16-item Reminder

**RAVLT** *Rey Auditory Verbal Learning Test*

**SAS** *Supervisory Attentionnal System*

**SPC** Psychological and Behavioral Symptoms

**SPI** Serial Parallel Independent

**TCL** Mild Cognitive Impairment

**ADD** Attention Deficit Disorder

**TMT** *Trail Making Test*

**UKPDBBS** *United*  *Kingdom*  *Parkinson's Disease Brain Bank Society*

**WAIS-IV** *Wechsler Adult Intelligence Scale - IV*

**ZBI-12** *Zarit Burden Interview – 12 items*

**Problematic**

Parkinson's disease (PD) is a neurodegenerative disorder characterized mainly, at the motor level, by tremors at rest and muscle rigidity. After Alzheimer's disease (AD), it is the second most prevalent neurodegenerative disease in the population aged 65 and over (Jellinger and Attems, 2015; Lees, Hardy, and Revesz, 2009; Williams-Gray, Foltynie, Brayne, Robbins, and Barker, 2007). In the general population, the risk of developing PD is estimated at 14 cases per 100,000 people, but in those 65 years of age and older, this risk increases to 160 cases per 100,000 people(Wirdefeldt, Adami, Cole, Trichopoulos, and Mandel,2011). Overall, the lifetime risk of developing PD is estimated to be about 1.3% in women and 2% in men (Wirdefelt et al., 2011).

In addition to the motor symptoms of PD, many other symptoms are observed (Aarsland et al., 2009; Kalbe and Kessler, 2015; Weintraub et al., 2015),such as depressive, apathetic, anxious and cognitive problems, ranging from mild cognitive impairment (MCI) to major cognitive impairment, also known as parkinsonian dementia. The proportion of PD patients who will develop MCI varies between 19 and 36% depending on the studies (Aarsland et al., 2009; Aarsland, Brønnick, and Fladby, 2011). Unfortunately, there is currently no cure for PD patients: there are only symptomatic treatments, which at most delay the progression of motor symptoms (Lees et al., 2009). Current research therefore looks at curative treatments and the identification of genes or risk factors associated with the development of PD (Jellinger and Attems, 2015). Despite these constant efforts to alter the course of the disease through pharmacological methods, there are currently very few alternatives for PD patients with cognitive impairment (Jellinger, 2006; Pagonabarraga and Kulisevsky, 2012; Sollinger, Goldstein, Lah, Levey, and Factor, 2010). To date, only one nootropic drug, rivastigmine, has been approved by Health Canada for the treatment of cognitive impairment in PD, and only for cases of dementia. Given that drug development can take several decades, other non-pharmacological therapeutic avenues may be useful to help patients who are currently struggling with MCI. This project therefore proposes to test the effectiveness of a cognitive training program that will be adapted to PD patients and that could improve or contribute to maintaining their autonomy on a daily basis. To provide context for the project, the following sections will first present the main neuropathological, neurochemical and clinical features of PD and MCI in PD. Then,studies that have already focused on cognitive interventions in PD will be presented and criticized. Overall, promising results emerge from these studies, but there is great variability in the methodologies used. It is therefore difficult to draw clear conclusions from these data. Also, few programs are really transferable in the daily lives of patients. In order to meet some of these limitations, the chosen training program, the "Goal*Management Training"* (GMT) will be addressed. The objectives ofthe two phases oftheproject, the hypotheses and the expected results will be set out. Then, the methodology that will be used to achieve the intended objectives will be explained and ethical considerations will also be presented. Finally, there will be a brief conclusion on the potential benefits of this project.

**Introduction**

*Presentation of Parkinson's disease (PD)*

PD was first described in 1817 by neurologist James Parkinson as "restless paralysis" (Parkinson, 1817). Subsequently, other symptoms were described during the^19th^ century, such as rigidity, scarcity of movement, micrography, and frozen facial expression, symptoms that are now part of the diagnostic criteria for the disease (Hughes, Daniel, Kilford, and Lees, 1992). PD is thus a disease best known for its motor symptoms, caused by significant alterations in the brain. However, several other non-motor manifestations are also frequently found, also induced by several brain lesions and dysfunctions (Dirnberger and Jahanshahi, 2013; Pagonabarraga and Kulisevsky, 2012).

Neuropathological and neurochemical bases

*Synucleinopathy.* It is generally accepted that PD is a proteinopathy, that is, a disease that involves abnormal protein aggregates in the brain (Jellinger, 2012; Lees et al., 2009). More specifically, PD is a synucleinopathy: it therefore involves abnormal aggregates of alpha-synuclein(α-synuclein), a protein whose role is still poorly understood. It is still known thatα-synuclein is involved in the recycling of neurotransmitters and the production of free radicals (Jellinger, 2012). α-synuclein aggregates form Lewy bodies, small cytoplasmic inclusions about 15 μm in diameter, used today as pathological markers for the post-mortem diagnosis of PD (Jellinger, 2012). Lewy bodies would bind to mitochondria and decrease their activity, eventually causing oxidative stress and neuronal death. In fact, Lewy's bodies would learn well before the onset of motor symptoms and spread from the brain stem to subcortical structures (black matter, dorsal nucleus of the vagus nerve, Basal Nucleus of Meynert), to reach the limbic system and frontal cortex during the more advanced stages of the disease (Jellinger, 2012; Lees et al., 2009). In addition, Lewy's bodies are found in the spinal cordandseveral nerves of the autonomic peripheral nervous system.

In order to characterize the course of the disease, Braak and colleagues(Braak, Del Tredici, Rüb, de Vos, Jansen Steur and Braak, 2003)established different stages of propagation of Lewy's bodies and were able to correlate them with motor and cognitive impairment. In stage 1, involvement of the medulla oblongata (motor nucleus and internal reticular zone) is observed. In stage 2, lesions and abnormalities reach the raphe nucleus, the gigantocellular reticular nucleus as well as the coeruleus and subcoeruleus locus. According to this model, stages 1 and 2 are clinically asymptomatic. In stage 3, damage to the black substance *pars compacta* is added to the previous lesions. In stage 4, the lesions reach the transentorhinal mesocotex and allocortex. In stage 5, the sensory associative areas of the neocortex and the prefrontal neocortex are affected. Finally, in stage 6, it is the primary and associative areas of the neocortex and the premotor areas that are reached. In general, these stages correlate with the severity of clinical symptoms of PD and stages 5 and 6 are correlated with the development of dementia (Braak et al., 2003).

In addition to Lewy bodies, PD patients also develop senile plaques formed by aggregation of amyloid-beta peptide_40-42_ (Jellinger, 2012) as well as neurofibrillary tangles, formed by hyperphosphorylation of the tau protein (Jellinger, 2012). However, it would seem that these latter alterations are rather a consequence ofα-synucleinaggregates,and not of the precursors of PD (Jellinger, 2012; for a review).

In PD, the volume of gray matter is reduced, including that of the basal ganglia, the midbrain in general, and the median temporal lobes(Zeighami, Ulla, Iturria-Medina, Dadar, Zhang, et al., 2015). Specifically, there is atrophy in the amygdala, hippocampus and other structures of the limbic system(Kehagia, Barker and Robbins, 2010). Also, the cortical volume in general decreases during the course of the disease(Kehagia et al., 2010).

*Neurochemical abnormalities*. The most important neurotransmitter loss in the neuropathology of PD is dopamine (DA) (Dirnberger and Jahanshahi, 2013; Kehagia et al., 2010). At the time of diagnosis, the degeneration of DA neurons of the black matter *pars compacta* would have already drastically decreased subcortical AD levels by 60% to 70% (Fearnley and Lees, 1991). This loss of AD occurs in particular in the basal ganglia, particularly in the striatum(Dirnberger and Jahanshahi, 2013; Kehagia et al., 2010). It would appear that neurons in the ventrolateral third of the black matter *pars compacta* would be the first to degenerate and then spread to the dorsal part (Fearnley and Lees, 1991; Kehagia et al., 2010). These structures are also strongly involved in sensorimotor control (Dirnberger and Jahanshahi, 2013). Degenerationwould first spread to the nigro-striatal DA pathway, and subsequently the structures of the mesocorticolimbic DA pathway would also be affected (Alcaro et al., 2007), contributing to certain cognitive symptomsin PD patients(Aarsland et al., 2011; Jellinger, 2006).

However, other neurochemical disorders are observed in patients and can partly explain motor and non-motor symptoms. First, cholinergic impairment of the basal nucleus of Meynert is also significant and can cause cognitive deficits, particularly in memory, visuospatial functions and attention (Kehagia et al., 2010; Müller and Bohnen, 2013). Indeed, during the course of the disease, cholinergic projections of the basal nucleus are affected, such as those reaching the occipital cortex, which contributes, in the more advanced stages, to visuospatial problems and visual hallucinations experienced by patients(Müller and Bohnen, 2013). Moreover, Lewy's bodies are found near the basal nucleus of Meynert. However, since acetylcholine (ACh) is also involved in the autonomic nervous system, cholinergic deficits may also have a role to play in postural instability problems and falls (Müller and Bohnen, 2013).

In addition, damage to the locus coeruleus has consequences on the projections of norepinephrine. This structure projects to the entire cerebral cortex, but also receives afferents from the limbic system, so that in the long run, the noradrenergic neurons of these structures will also be affected. Some authors have also noted that, via projections to the prefrontal cortex, the noradrenergic system would be involved in the formation of adaptive behaviors during a new or stressful situation. Altering these projections would therefore have impacts, among other things, on planning and organizational capacity in PD patients (Del Tredici and Braak, 2013).

It also seems that there is a degeneration in the serotonin system. Even in the early stages of the disease, a loss of serotonergic neurons is observed on the one hand in the raphe nucleus, and on the other hand in the striatum (Politis and Niccolini, 2015). This degeneration is thought to be involved in many of the non-motor symptoms, including sleep problems, depressive and anxiety symptoms, which may be present from the very beginning of the disease (Politis and Niccolini, 2015).

Finally, glutamatergic dysfunctions are observed, but this would actually be a consequence of the depletions of AD and ACh. These deficits would occur later in the later stages of the disease, and in Parkinson's dementia (Jellinger, 2012; Kehagia et al., 2010; Lees et al., 2009).

All neurochemical disorders will have long-term repercussions on the anatomical structures that have been linked to themand will contribute to the neuropathological alterations mentioned above.

Riskfactors associated with PD

As with the etiology of Alzheimer's disease(AD), that of PD is still unknown to this day. The main hypothesis considered would be a combination of genetic vulnerability and environmental factors that would modulate the magnitude of neuronal loss (Schapira and Jenner, 2011).

*Genetic vulnerability*. Genetic susceptibility would be involved in 10 to 15% of PD cases and therefore, the majority of cases would be rather idiopathic in nature (Lesage and Brice, 2009). Mutations in the*"Leucine-Rich Repeat Kinase 2"*(LRRK2) and α-synuclein genes are direct factors in the autosomal dominant familial form of PD(Sai, Zou, Peng, and Dong, 2012; Zimprich, Biskup, Leitner, Lichtner, Farrer, et al., 2004). Also, alterations in  *the parkin* and*PTEN-Induced Putative Kinase 1*(PINK1) genes, playing a role in mitochondrial integrity and protection against oxidative stress, are also linked to the development of PD (Sai et al., 2012). It should be noted that other polymorphisms have also been identified, but their contribution is not yet fully clarified; therefore, they will not be addressed in this document.

*Environmental factors.* Several toxins are associated with the development of PD. The most well-known substance related to PD is 1-methyl-4-phenyl-1,2,3,6-tetrahydropyridine (MPTP), discovered by mistake during the 1970s (Cicchetti, Drouin-Ouellet, and Gross, 2009). Indeed, an injection causes irreversible symptoms in humans similar to those of PD and also explainable by a massive loss of AD (Cicchetti et al., 2009; Pan-Montojo and Reichmann, 2014; Tanner, 1989). In the same vein, it has been noted that living in rural areas increases the risk of developing PD(Pan-Montojo and Reichmann, 2014; Tanner, 1989), in particular due to exposure to pesticides and heavy metals (Pan-MontojoandReichmann, 2014).

Specifically for cognitive impairment in PD, other risk factors are beginning to be investigated. Indeed, it has been suggested that the presence of vascular risk factors such as hyperhomocysteinemia and smoking may be associated with the development of cognitive disorders in PD(Doiron, Dupré, Langlois, Provencher and Simard,in press; Doiron and Simard, 2012). However, the importance of the presence of vascular risk factors in the development of cognitive impairment and dementia in PD remains a controversial topic.

Clinical presentation of PD

*Motor presentation.* At the clinical level, PD is primarily diagnosed by the presence of motor impairments in patients(Hughes et al., 1992; Lees et al., 2009). Indeed, even if neuronal loss begins many years before the onset of symptoms, the great magnitudeof cases is diagnosed at the time of the onset of motor symptoms. Motor symptoms are mainly summarized by tremors at rest, unilateral at the beginning of the disease, and / or muscle rigidity (Hughes et al., 1992). There is also bradykinesia, i.e. slowness in movement, postural instability and micrography, i.e. smaller and smaller writing (Hughes et al., 1992; Lees et al., 2009) . A final important point to mention is a positive response to Levodopa (L-DOPA), a drug that acts as a precursor to AD (Lees et al., 2009).

For diagnosis, the criteria mentioned above are those of the*"United*  *Kingdom*  *Parkinson's Disease Brain Bank Society"*(UKPDBBS) which are currently the most widely used in the world (see Annex 1.1 for a description) (Hugheset al., 1992). Other diagnostic criteria have also been developed by the*National Institute of Neurological Disorders and Stroke* in the United States (NINDS;see Table 1.2 of Appendix 1)(Gelb, Oliver, and Gilman,1999). Overall, the US criteria are similar to the UKPDBBS criteria,although some differences exist. While bradykinesia is a mandatory UKPDBBS criterion, it is not required according to NINDS criteria (Doiron and Simard, 2012; for a review). In addition, among the criteria supporting the diagnosis of probable PD, the NINDS criteria indicate that tremors or stiffness must affect the side of the onset of the disease more severely, while the UKPDBBS mentions that the onset of symptoms must be unilateral (Hughes et al., 1992). Recently, the*Movement Disorder Society*hasrevised the diagnostic criteria for PD(Postuma, Berg, Stern, Poewe, Olanow et al., 2015). This time, a vision in terms of positive symptoms (supporting the diagnosis) and negative (suggesting other explanations) was adopted, and the obligatory manifestations are bradykinesia, rigidity and tremor at rest. Table 1.3 of Appendix 1 provides a detailed description of these new diagnostic criteria.

In order to characterize the course of the disease from a clinical point of view, the Hoehn and Yahr stages, established in 1967 and revised in 2004, are generally used to classify patients withmotor impairment (Goetz, Poewe, Rascol, Sampaio, Stebbins etal., 2004; see Appendix 2).

*Non-motor*presentation.

Vegetative, autonomous and sensory symptoms. PD involves many non-motor symptoms that impact the functioning of the individual. Pd patients complain, among other things, of peripheral autonomic nervous system symptoms, such as orthostatic hypotension,gastrointestinal pre-oblitera and constipation at the onset of disease, which are potentiated by L-DOPA medication (Chaudhuri and Schapira, 2009). Later, in the advanced stages, some patients may suffer from urinary and fecal incontinence (Chaudhuri and Schapira, 2009). In addition, symptoms of the sensory system are observed by patients, including olfactory (anosmia) and taste dysfunctions that are among the first observations made by patients even before the onset of motor symptoms (Chaudhuri and Schapira, 2009).

Psychological and behavioral symptoms. Psychological and behavioural symptoms (SPC) are also reported by patients and their relatives. On average, in about 40% of patients, apathy and depressive or anxiety symptoms concomitant with PD are found at one stage or another of the disease (Leentjens, Dujardin, Marsh, Martinez-Martin, Richard et al. , 2008; Williams et al., 2012). In a review of the literature, Weintraub and Burn (2011) estimated that between 5% and 20% of patients met the diagnostic criteria for major depression according to the DSM-IV at the time of diagnosis of PD, and that between 10% and 30% of other patients had clinically significant depressive symptoms. The most recent data on the subject point in the same direction:they show that de *novo* patients with PD are twice as likely (14% of the sample) to have clinically significant depressive symptoms than control subjects (7%) matched according to age andlevel ofeducation (Weintraub et al., 2015). Apathy was three times more common in de *novo* PD patients (17% versus 5% for controls). In the same sample, three times as many PD patients had clinically significant anxiety symptoms, compared to controls (24.6% versus 7%). However, it is unclear whether these symptoms are a neurological consequence of brain damage or whether they are a psychological reaction to diagnosis and the increasing difficulty of performing daily tasks (Pirogovsky, Schiehser, Obtera, Burke, Lessig et al., 2014).

Others SPC Include some visual hallucinations that occur often plus late in the evolution of PD (Aarsland et al., 2009), an increased risk of developing a holePulse control ble (Gallagher, O'Sullivan, Evans, Lees and Schrag, 2007), and sleep disturbances (Adler and Thorpy, 2005). Concerning the latter, studies estimate that up to 90% of patients with PD may have sleep problems (Weintraub and Burn, 2011). In particular, the REM sleep disorders would be frequently observed in prodromal phase pd, but others Symptoms, such as daytime fatigue, unionizationome restless legs or terrors nocturnes, have also been reporteds (Claassen, Josephs, Ahlskog, Silber, Tippmann-Peikert and Boeve, 2010).

It is important to note that SSC, by itself, may contribute to the cognitive deficits seen in PD patients. For example, an apathetic patient may have impaired speed of information processing, then this could contribute, with the motor impairment, at this timed tasks are less successful (Palavra, Naismith & Lewis, 2013). In addition, pfor a depressed patient, the necessary motivation and concentration to complete a cognitive task Can be difficults to be found (Leentjens et al., 2008). Although SSC are numerous and frequent, cognitive symptoms Remain among the most complaints recurrentes and also alter the quality of life of patients.

*Mild cognitive impairment (MCI) in PD*

Interest in mild cognitive impairment in PD is relatively recent, as it was often mentioned, until the early 1980s, that alterations noted on cognitive tests were caused by motor problems experienced by patients and that cases of dementia occurred only in the advanced stages of PD (Sollinger et al., 2010). On the other hand, it is now known that MCI in PD is not only considered an intermediate stage towards the development of Parkinson's dementia, but it can also be an integral part of the clinical picture of PD from the onset of the disease (Litvan, Goldman, Tröster, Schmand, Weintraub, Petersen et al. , 2012) . Indeed, subjective cognitive complaints and objective cognitive deficits are observable in newly diagnosed patients, even when they have very few motor impairments (Aarsland et al., 2011). It should be noted that according to the different studies, between 40% and 50% of patients would meet the criteria for MCI during the course of the disease, but in *de novo*patients, without medication, the most likely incidence was established at approximately 27% (between 19% and 36%; Aarsland et al., 2009). More recently, a study in *de novo* Parkinson's patients reported prevalences ranging from 3% to 22%, depending on the diagnostic criteria used and the rigour of neuropsychological assessment (Weintraub et al., 2015). For example, studies that use measures of overall cognitive functioning, such as the*Mini-Mental State Examination*(MMSE) or theMontreal Cognitive*Assessment*(MoCA), achieve a higher prevalence of patients with MCI, compared to studies that perform a more comprehensive cognitive assessment in using a standardized neuropsychological battery (Weintraub et al., 2015).

MCI is defined as a minor impairment in cognitive functioning, either in a single cognitive domain (single-domain TCL) or in multiple cognitive domains (multi-domain TCL)(Litvan et al., 2011; Petersen, 2004; 2012; Tröster, 2011). The Petersen decision tree for characterizing the TCL is found in Appendix 3. Recently, Litvan et al. (2012) established guidelines for the diagnosis of MCI in PD. Subsequently, the nomenclature used in the text to designate these patients will be 'MP-TCL patients'. Table 1 summarizes these criteria. MCI can be amnesic, during memory impairment, or non-amnesic, during impairment of any other cognitive domain (Litvan et al., 2012). Several studies have established that single-domain non-amnesic MCI, with impairment of executive functions, is more common in PD patients (between 13% and 62% depending on the studies; Aarsland et al., 2009; Matteau, Dupré, Langlois, Provencher, and Simard, 2012) and an important predictor of the development of dementia (Litvan et al., 2011). For multi-domain non-amnesic MCI, the prevalence is as high as 50%, depending on the characteristics of the patients included in the studies (Matteau et al., 2012). The proportion of AMNES-TYPE MP-TCL patients varies between 9% and 25%, whether single-domain or multi-domain (Aarsland et al., 2009; Matteau et al., 2012). Thus, the wide variability in prevalence between different studies shows how heterogeneous cognitive profiles in PD are (Tröster, 2011).

| Diagnostic criteria for MCI in the MP of Litvan et al., 2012 |
| --- |
| Diagnosis of PD brain *bank society* (Hughes et al., 1992) |
| Gradual decline in cognitive skills, as reported by the patient, caregiver and/or clinician, who performed one of twotypes of assessment. |
| Type 1 neuropsychological assessment  Impaired cognitive functioning as measured by a validated global cognition scale in patients with PD (e.g., MoCA; *Parkinson Disease Cognitive Rating Scale;* *Mattis Dementia Rating Scale*).  or;  Impairment of cognitive functioning, as measured by results between 1 and 2 standard deviations below the mean (standards according to age and/or level of education) obtained on at least two standardized tests when administering a brief neuropsychological battery including one test per cognitive domain or assessing less than five cognitive domains.  This assessment makes it possible to make a possible diagnosis, but one that is not as certain as for the type 2 assessment.  Neuropsychological assessment type 2  Objective cognitive decline, as measured by results between 1 and 2 standard deviations below the mean (standards by age and/or level of education) obtained on at least 2 tests in the same cognitive domain or in different cognitive domains among the following:  1) attention and working memory,  2) executive functions,  3) language functions,  4) memory (episodic),  5) visuo-spatial functions.  Two neuropsychological tests per domain (full neuropsychological assessment battery) must be performed to confirm the objective cognitive impairment of the participant. |
| Difficulties do not significantly interfere with daily activities, but minor difficulties are sometimes observed. |

*Table 1: Diagnostic criteria for MCI in a PD context according to Litvan et al. (2012).*

Although it is impossible to draw a complete picture of cognitive impairment in PD, some deficits have been observed and replicated by several studies. It should again be emphasized here that the cognitive profile of MP and MP-TCL patients is generally heterogeneous, so none ofthedisorders mentioned below are systematically found in all MP-TCL patients (Monchi,Hanganu, and Bellec,2016).

Language

It is possible to divide language into several sequential components, one of which is related to the perceptual aspect (hearing / vision), another to the interpretative aspect (comprehension / semantics) and a last to themotor aspect (planning / production)(Vertstichel and Cambier, 2005). Because of the motor impairment that eventually occurs during PD, patients may experience speech and joint problems, such as dysarthria and dysprosody (Poletti, Emre, and Bonuccelli, 2011a). In fact, some authors have drawn a parallel between patients with Broca's aphasia and some PD patients, as it would appear that language production is more difficult at the beginning of PD, but that understanding would remain intact (Bastiaan and Lenders, 2009). At the level of verbal production, it would be verbal fluency, especially lexical, that would be reduced in the MP-TCL (Poletti et al. , 2011a). At the interpretative level, the comprehension of more complex sentences also becomes more difficult during the more advanced stages of MP-TCL and the early stages of Parkinson's dementia, which could be related to the impairment of temporal regions that occur later, regions involved in the semantic aspect of language (Bastiaan and Lenders, 2009). Problems in the proper use of grammatical rules may also be present (Bastiaan and Lenders, 2009). A hypothesis was made that it was the sequence necessary to adequately produce understandable words and phrases that would be deficient and that would prevent language production in PD patients. The ability to plan a sequence of language would also be related to the activity of the basal ganglia,affected in PD (Bastiaan and Lenders, 2009).

Careful

According to the model of Van Zoomeren and Brouwer (1994), attention would be divided into several levels in terms of intensity and selectivity. In terms of intensity, the phasic alert makes it possible to react quickly to a non-specific stimulus while sustained attention allows you to maintain your attention for a long time. In terms of selectivity, selective attention makes it possible to ignore irrelevant stimuli while divided attention makes it possible to share attention between several stimuli. These levels are all overseen by a central system: supervisory attentional control, an element later added and inspired by Norman and Shallice's (1986)*Supervisory Attentional System*(SAS) model. This SAS system is flexible, develops strategies to achieve a goal and findssolutions in theevent of a new situation.

In some PD patients, significant fluctuations are noted in internal attention control as well as difficulties with tasks requiring selective, sustained and divided attention (Dirnberger and Jahanshahi, 2013; Dujardin, Tard, Duhamel, Delval, Moreau et al., 2013). For example, computerized tasks requiring reacting as quickly as possible to any stimulus, reacting only to one type of stimuli by ignoring others (Go paradigm – No Go) or alternating between two response patterns, are often used to measure attentional abilities (Dujardin et al., 2013). One hypothesis raised to explain these disorders would be that Parkinson's patients would have more difficulty mobilizing their attentional resources during a non-routine task, which would be consistent with the deficits in inhibition of automatic responses also observed (Dirnberger and Jahanshahi, 2013; Kudlicka,Clare and Hindle,2011). However, in addition to attentional control difficulties that are probably more related to working memory and executive control, attention problems generally do not appear to be at the forefront of cognitive complaints (Aarsland, 2016).

Working memory

With regard to working memory, Baddeley (2010) defines it in his model as a system that allows to temporarily maintain and manipulate information necessary for comprehension, learning and reasoning. In the latest version of the model, a suprasystem, the central administrator, would coordinate three subsystems: the phonological loop, the visuospatial notebook, and theepisodic buffer (Baddeley, 2010). The phonological loop allows to retain briefly (a few seconds) and manipulate verbal information and would be involved in the learning of vocabulary and language. The visuospatial notebook takes care of retaining visual information, forming mental images and manipulating them in space. Theepisodic buffer was later added to this model and ensures interaction with long-term memory. In other words, it allows information in long-term memory to resurface to consciousness and it also allows information to be transferred from working memory to long-term memory, regardless of the modality. In several MP-TCL patients, it would be the manipulation of information, and especially visuospatial information, that would be altered(Parnetti and Calabresi, 2006; Pagonabarraga and Kulievsky, 2012). In Baddeley's model, this would therefore reflect deficits at the level of the visuospatial notebook and the central administrator. For example, Parkinson's patients would be sensitive to tasks such as theWeschler Adult Intelligence Scale – IV (WAIS-IV) batteryback, Corsi blocks (Pagonabarraga and Kulievsky, 2012), and the dual-task paradigm in which a participant's performance when doing two tasks each in isolation is compared with their performance when they have to do two tasks at the same time (e.g., repeating sequences of numbers and solving a maze). on a sheet of paper)(Sala, Baddeley, Papagno and Spinnler,1995).

Episodic memory

According to Tulving's (1995) Serial, Parallel and Independent (SPI) model, memory separates into several components and information would be serialized, stored in parallel, and retrieved independently. In particular, Tulving distinguishes between episodic memory, related to our past experiences in relation to our self-awareness (autonoetic memory), and semantic memory, which will be described in the next section. Both types of memory can contain visual or verbal information. Three processes would be involved in episodic memory: encoding, consolidation and retrieval of information. Encoding is the process of associating new information with a known context. Rather, consolidation is related to the process of storing information. Retrieval is the process by which encoded and consolidated information is transferred from long-term memory to working memory in order to be used again. In some MP-TCL patients, specific episodic memory deficits have been reported. Encoding and consolidation processes would be preserved, although they are slower than average (Barker and Williams-Grey, 2014; Monchi et al., 2016; Poletti et al. , 2011a). Retrieval of information would be deficient, especially with respect to the free recall of information (Pagonabarraga and Kulievsky, 2012). Difficulties with tasks sensitive to these processes, such as the California Verbal*Learning Test* (CVLT) and the Visual*Retention Test,* are frequently reported in the literature in PD patients (Kehagia et al., 2010; Pagonabarraga and Kulievsky, 2012). Deficits are generally more pronounced in visual modality, but some patients have more difficulty in verbal modality (Pagonabarraga and Kulievsky, 2012).

Semantic memory

Still according to the SPI model of Tulving (1995), semantic memory would be complementary to episodic memory and would concern verbal or visual information not related to autobiographical memories, such as knowledge of the world, objects, events, all independent of the concept of self (anoetic) (Tulving, 1995). To measure it, it is possible to use tasks in which a participant must answer general knowledge questions or to classify objects in a semantic category; for example, is a butterfly an animal, a plant or an object? (Guidi, Paciaroni, Paolini, Scarpino, and Burn,2015). In MP-TCL patients, deficits in correctly classifying an object into a semantic category have been reported, although the ability to correctly name semantic categories is presumed (Guidi et al., 2015). It has been proposed that the executive aspects of semantic memory, such as the retrieval and manipulation of information in memory, are more impaired than encoding and storage processes, consistent with reported deficits in episodic memory (Guidi et al., 2015).

Procedural memory

Anderson's Adaptive*Control of Thought*(ACT) model divides information into two types: declarative, related to facts and knowledge expressible through language, or procedural, which deals with the memory of movements and routines (Anderson, 1982; Anderson and Schuss, 2000). Procedural learning would be done by following three steps. First, the cognitive phase would involve other memory processes, such as episodic memory and working memory. The person learns the steps necessary to complete the activity. Then, during the associative phase, by dint of practice and repetition of a task, the other memory processes would be less and less involved and finally, during the autonomous phase, thenew learning would become automatic,and therefore would become a routine (Anderson, 1982; Anderson and Schuss, 2000). According to some studies, procedural learning, especially at the level of the autonomous phase, is achieved in some PD patients without alteration to another cognitive domain,(Doyon, Gaudreau, Laforce, Castonguay, Bedard et al., 1997; Doyon and Laforce, 2001). For example, deficits have been reported in some PD patients for tasks such as "Serial*Reaction Time",*in which the participant isasked to correctly associate a motor sequence with a stimulus on a computer screen (Doyon et al., 1997; Doyon and Laforce, 2001). These alterations are explained, among other things, by damage to the striatum, which is directly involved in learning a routine (Doyon and Laforce, 2001).

Visuospatial functions

Boller's work in the 1980s was among the first to work on characterizing difficulties in visuospatial functions in PD. Visuospatial functions were then defined as the set of processes for estimating the relative position of a stimulus or object in space, integrating these objects into a mental image and mentally manipulating these objects according to spatial concepts (e.g. moving, turning, rotating or symmetrical,etc.). ) (Boller, Passafiume, Keefe, Rogers, Morrow et al., 1984). In neurobiology, two separate pathwayshave been identified as being involved invisuospatial functions:the*"what"*pathwayand the*"where"*pathway(Kolb and Winshaw, 2008). Both pathways start from the visual areas (striated and extra-striated cortex) and would relay information in parallel. The*"what"*pathway, also called ventral, correctly recognizes an object in the field of vision and would pass through the right median temporal lobe to get to the level of the inferior temporal cortex. The*"where"*pathway, also called the backbone, makes it possible to make a mental image of thelocation of an object in space and to coordinate movements according to this image. This pathway would rather pass through the right parietal lobe, but it would also end at the level of the inferior temporal cortex (Kolb and Winshaw, 2008). Several deficits in visuospatial functions have been observed and some of the previously reported deficits are related to the processing of visuospatial information(Parnetti and Calabresi, 2006; Pagonabarraga and Kulievsky, 2012). Notably, deficits in the perception and organization of space are observed, in additionto problems with mental rotation (Pagonabarraga and Kulievsky, 2012; Parnetti and Calabresi, 2006; Poletti, et al. , 2011a). Several tests used in PD patients have demonstrated their sensitivity to evaluate these processes. These tests include the Benton line orientation judgment test and the Hooper visual organization test (Boller et al., 1984). Visuospatial deficits are thought to be related to the subsequent development of visual hallucinations (Pagonabarraga and Kulievsky, 2012). Authors have noted that the dorsal caudate nucleus, reached early in the development of PD, is anatomically very close and connected to the right inferior parietal lobule, involved in spatial cognition (Kesner and Creem-Regehr, 2013). This suggests that, early in the development of PD, the*"where"*pathwould be reached, but this would not necessarily be the case for the "what" path.

Executive functions

Executive functions are high-level mental skills used in organizing, planning, integrating multiple sensory modalities, inhibiting automatic behaviors, and solving problems(Smith and Jonides, 1999). They would make it possible to mobilize other cognitive functions and coordinate them during actions directed towards a goal. Several theoretical models attempt to explain executive functions.

A first well-known model is that of Norman and Shallice (1986), who proposed a hierarchical model of action control, divided into three components. The first is the inventory of action patterns, which makes it easy to plan activities already learned and requires a minimum level of attention. This level of operation is similar to the design of anderson's procedural memory automation phase. The second component is the schema conflict handler, which selects the most appropriate schema for a situation when two or more schemas are enabled at the same time. The third is the*Supervisory Attentional System*(SAS), which handles new situations, which are not very routine, or when known patterns are not adapted. It would be the supervisory system that would require the most attentional resources and would take care of planning, decision-making, problem solving and inhibition, among others.

Second,according to Duncan's (1986) theory, frontal lesions, regardless of their origin, lead to general deficits in goal-oriented actions, which explains the wide variety of difficulties that injured patients can experience on a daily basis. Duncan (1986) describes these deficits as executive dysfunctions, that is, a problem of organizing thought and action. According to his theory, the sequences necessary to accomplish an objective would be fragmented or incomplete, resulting in omissions (of sequences essential to the task) or intrusions (of sequences not essential to the task).

It was noted that even in patients did not presentNt not clinically PD, but being genetically at risk, executive functions swould be altered (Hawkins, Jennings, Marek, Siderowf and Stern, 2010). In patients MP-TCL that have infringements of executive functions, problems inhibiting automatic responsessuchs than measured by the Stroop task, Would often observed (Kudlicka et al., 2011; Kehagia et al., 2010). Also, patients have deficits in Thealternating response patterns or at the level of the capacity for mental flexibility («*set shifting*»), as measured by the condition of alternation of verbal fluency tests. Lhas the capacity to problem solving, as well as the speed of decision-making would also be Deficit (Dirnberger and Jahanshahi, 2013; Kehagia et al., 2010; Kudlicka et al., 2011). For Parkinson's patients, goal-oriented actions are therefore more difficult to accomplish, from the planning stages to concrete actions. to achieve this (Dirnberger and Jahanshahi, 2013; Kehagia et al., 2010). Based on Norman and Shallice's model, some authors have proposed that systems requiring minimal attention would be achieved in PD (e.g. the directory of routine schemas and the conflict handler between schemas). Thus, even for routine tasks, PD patients should use more cognitive resources by using the supervising system (Dirnberger and Jahanshahi, 2013). Based on Duncan's model, some PD patients presentstripeNt executive dysfunction, in particular with regard to coordinating all necessary actionss the achievement of an objective, due to infringements striato-frontal.

Theory of mind

Theory of mind can be defined as the ability to understand and predict another person's behaviors by attributing to them mental states independent of their own(Bora, Walterfang and Velakoulis,2015; Poletti, Enrici, Bonuccelli, and Adenzato, 2011b; Premack and Woodruff,1978). Since perception and response to social stimuli require a complex interaction between emotion and cognition, several different cortical and limbic structures, such as the parietotemporal junction, the prefrontal cortex (dorsolateral and ventromedial) and the amygdala are involved in these processes (Freedman and Stuss, 2011). In order to measure theory of mind in adults, several standardized tests are used in research. These can be cognitive (related to the processing of thoughts), affective (related to the processing of emotions) or both. First, the "First*Order False Belief Test"*and the"Second*Order False Belief Test"*respectively test whether a participant is able to understand that another person may have a wrong view of the world and whether a participant is able to imagine the reflection that another person can do (Poletti et al., 2011b). Another task often used is that of "Reading*the Mind in the Eyes".* In this test, a participant is asked to guess what another person, shown in a photo, thinks and feels. It is therefore a more emotionally focused task (Poletti et al., 2011b). Finally, a final test often reported in studies is that of the "Fauxpas" which consists of telling 10 stories in which a person makes a subtle but important clumsiness, as well as 10 control stories, without missteps; the participant must correctly explain the clumsiness when present (Poletti et al., 2011b).

In Parkinson's patients, even at the onset of illness, social cognition and theory of mind are deficient (Dirnberger and Jahanshahi, 2013; Kudlicka et al., 2011; Poletti et al., 2011b). Several studies in PD patients who were given the Misstep task showed that the cognitive component was affected, but not the affective component, in the absence of other cognitive deficits(Kawamura and Koyama, 2007; Péron, Vicente, Leray, Drapier, Drapier et al., 2009; Roca, Torralva, Gleichgerrcht, Chade, Arévalo et al., 2010). A recent meta-analysis on the subject showed that compared to health controls, PD patients performed less well on the three tasks mentioned above, with a high effect size (all tasks combined, Cohen's *d* = 0.83). In particular, cognitive tasks such as firstand second order false belief*tests*hadthe highest effect sizes (Cohen's*d*  = 1.26; Bora et al., 2015). Moreover, the deficits were less marked at the beginning of the disease and worsened over time.

It has been hypothesized that damage to the frontal-triatal circuits, particularly the dorsolateral prefrontal loop, contributes to the difficulties of some PD patients in tests measuring theory of mind (Bora et al., 2015; Poletti et al., 2011b). A final interesting aspect that emerges from theory of mind studies is that executive functions as measured by the alternating condition of the verbal fluency test were a good predictor of theory of mind test scores (Bora et al., 2015). One study even showed that a cognitive training program, targeting memory and executive function, conducted in MP-TCLpatients, improved performance at a theory of mind task based on the same model as the Misstep task (Pena, Ibarretxe-Bilbao, García-Gorostiaga, Gomez-Beldarrain, Díez-Cirarda and Ojeda, 2014). Indeed, there are on the one hand several anatomical substrates common between executive functions and theory of mind and, on the other hand, these functions are both related to self-awareness, which is very important in the coordination of planned actions and sequences. Whether executive dysfunctions fully explain theory of mind deficits in PD patients is still a point of debate in the literature (Bora et al., 2015; Freedman and Stuss, 2011).

Summary of frequently used neuropsychological tests

Table 1.4 of Appendix 1 shows different tests that can be used and that are considered to be sensitive enough for the assessment of MCI in PM (although the list is not exhaustive).

Activities of daily living

Activities of daily living (ADLs) are divided into two types: basic ADLs, which are used primarily to meet the physiological needs of the individual, such as feeding or washing, and instrumental activities of daily living (AIVQ). Care Latest are defined as complex activities, necessary for the proper daily functioning. For example, they include tasks such as housekeeping, effective medication management, or telephone communication (Shulman, Pretzer-Aboff, Anderson, Stevenson, Vaughan, Gruber-Baldini and Weiner, 2006).

Although no major alteration of operation daily is found in the MP-TCL, the presence of a TCL may cause minor difficulties in the realization of AIVQ (Rosenthal et al., 2010) and thereby affect the quality of life of patients and their loved ones (Dirnberger and Jahanshahi, 2013; Lawson et al., 2014; 2016; Schrag, Jahanshahi & Quinn, 2000). In patients MP (stages II and III of Hoehn and Yahr), some of these activities become more difficult to accomplish, such as cleaning and manage medications (Schulman, Gruber-Baldini, Anderson, Vaughan, Reich, Fishman and Weiner, 2008). One study also reported that theAlteration ofhe executive functions, in particular the difficulty to plan, allowstait to significantly predict the difficulty of realizeder AIVQ in the PD (Cahn, Sullivan, Shear, Pfefferbaum, Heit and Silverberg, 1998). In addition, another study showed that patients PM tend to overestimate their functioning for four AIVQ tasks: medication management, food, clothing and money management, when their self-assessment is contrasted with Theobjective assessment Carried out by a clinician (Shulman et al., 2006).

The achievement of AIVQs, and therefore indirectly the autonomy of PD patients, seem to be related to executive functioning. All of these components would be relevant toa patient's assessment. In order to help PD patients remain independent for as long as possible, some pharmacological and symptomatic treatments are offered. They have demonstrated some effectiveness in treating certain symptoms for a few years. The different treatment options offered will be described in more detail in the next section.

*Pd treatments*

Antiparkinsonians

In order to control the motor symptoms of the disease, the main mechanism of action used today is to compensate for the loss of DA by pharmacological agents. One of the main antiparkinsonian agents used is L-DOPA (levodopa-benserazide; Prolopa®; levodopa-carbidopa; Sinemet®), a direct precursor to the DA which can easily cross the blood-brain barrier, unlike dopamine which cannot cross it (Jellinger, 2012; Paul and Bonuccelli, 2013).L-Dopa should be combined with a DOPA-Carbaoxylase inhibitor, such as Prolopa benseraside or Sinemet carbidopa, which decreases the conversion of L-Dopa to dopamine at the periphery and causes more L-Dopa to cross the blood-brain barrier... This co-medication is effective in reducing tremor symptomss and rigidity. (Connolly and Lang, 2014). However, the maximum efficiency period is on average only 5 years from diagnosis, then the effectiveness gradually decreases thereafter (Connolly and Lang, 2014; Paul and Bonuccelli, 2013). Periods of fluctuations in the effectiveness of antiparkinsonians eventually develop, called ON periods and OFF (Connolly and Lang, 2014). Other antiparkinsonian agents exhibit other mechanisms of action, such as the Agonists DA (bromocriptine, Parlodel®; pramipexole, Mirapex®; ropinirole, ReQuip®), the monoamine oxidase B inhibitors (MAOIs-B; selegiline, Eldepryal® or rasagiline, Azilect®) and inhibitors of Catechol-O-Methyltransferase (COMT; entacapone, Comtan®). COMT is an enzyme that degrades DA in synaptic space (Poletti and Bonuccelli, 2013). Its inhibition therefore allows AD to remain longer in the synaptic space.

However, the effects of these drugs on cognition are currently controversial (Robbins and Cools, 2014). Some studies have noted cognitive improvements in their patients, particularly in executive functions and working memory, while other studies have indicated the opposite (Liepelt-Scarfone, Gräber, Fruhmann Berger, Feseker, Baysal, Csoti et al., 2012; Poletti and Bonuccelli, 2013; Robbins and Cools, 2014). In fact, it would appear that certain cognitive functions, such as inhibition capacity, mental flexibility, divided attention, and working memory are sensitive to DA medication via the striatal-prefrontal circuits (Kehagia et al., 2010). Conversely, some visuospatial functions, such as mental rotation capacity and visuospatial memory, would not be affected by the depletion of AD, which would explain the differences found in the different studies (Kehagia et al., 2010). A 2013 meta-analysis on the subject also showed that at the beginning of the disease, the majority of patients benefited from antiparkinsonian medication cognitively, but that a great heterogeneity settled in the more advanced stages of the disease, and varied among other things according to the ON-OFF effects of the medication after a few years of administration (Poletti and Bonnucelli, 2013).

Nootropics

In addition to the DA disorders characteristic of PD, cholinergic disorders are also observed (Müller and Bohnen, 2013). In order to counter these symptoms, the treatment is the same as for AD: these are cholinesterase inhibitors (IChEs). Acetylcholinesterase (AChE) and butyrylcholinesterase (BChE) are enzymes usually used to break down ACh in the synaptic space. The goal of IChE is therefore to increase the amount of ACh available in the synaptic cleft (Rolinski, Fox, Maidment, and McShane, 2012).

A first IChE, donepezil (Aricept®), reverses AChE (Cheewakriengkrai and Gauthier, 2013). Various studies have recently published positive results regarding its effectiveness for the treatment of dementia in PD (Pagano et al., 2015; Rolinski et al., 2012; Wang et al., 2015). However, donepézil is not officially approved by Health Canada for the treatment of cognitive impairment in PD.

A second IChE, rivastigmine (Exelon®), is officially approved by Health Canada for the treatment of parkinsonian dementia, so it would only be effective for more disabling and disturbing cognitive symptoms. It is a non-competitive inhibitor of both AChE and BChE that is almost not biotransformed (Stahl, 2008).

Second, galantamine (Reminyl®) is a competitive and selective inhibitor of AChE, but it is also an allosteric modulator of nicotinic cholinergic receptors (Stahl, 2008). In PD with dementia, a few studies have looked at galantamine, but the results did not appear positive (Connolly and Lang, 2014; for a review).

The latest drug approved for the treatment of AD has a somewhat different mechanism of action. Memantine (Ebixa®) primarily targets voltage-dependent channels of glutamate N-methyl-D-aspartate receptors (NMDA). Memantine is indeed a non-competitive antagonist of these channels, which decreases the binding of glutamate to NMDA receptors and therefore, there would be a decreasing effect on the excitotoxicity of glutaminergic neurons (Stahl, 2008). Very few studies have been conducted in MP patients with memantine, none of which have been conducted in MP-TCL patients (Connolly and Lang, 2014).

Thus with respect to the treatment of MCI in PD, no drugs are currently approved by either Health Canada or the "*Food and Drug Administration*(FDA). Given this, many researchers in the field have noted that non-pharmacological approaches could improve the daily functioning of patients currently struggling with one TCL (Hindle, Petrelli, Clare, and Kalbe, 2013; París et al., 2011; Petrelli, Kaesberg, Barbe, Timmermann, Fink, Kessler and Kalbe, 2014; Petrelli, Kaesberg, Barbe, Timmermann, Rosen, Fink et al., 2015; Zimmermann, Gschwandtner, Benz, Hatz, Schindler, Taub, and Fuhr, 2014). Indeed, teaching new cognitive strategies to compensate for deficits could respond to main cognitive complaints that PD patients often express (Angelucci, Peppe, Carlesimo, Serafini, Zabberoni, Barban, et al., 2015; Costa, Peppe, Serafini, Zabberoni, Barban et al., 2014; Hindle et al., 2013; Milman, Atias, Weiss, Mirelman, and Hausdorff, 2014; Mohlman, Chazin, and Georgescu, 2011; Nombela, Bustillo, Castell, Sanchez, Medina, and Herrero, 2011; Sammer, Reuter, Hullmann, Kaps, and Vaitl, 2006; Reuter, Mehnert, Sammer, Oechsner, and Engelhardt, 2012). In recent years, non-pharmacological approaches have aroused the interest of many researchers and the number of publications on these approaches in PD has exploded since 2010. Despite the fact that research continues in the pharmaceutical industry, non-pharmacological avenues would be a good complement to cpatients who are often already taking several types of medications. Non-pharmacological approaches, and more particularly cognitive interventions, Would less risky in termss adverse effects and less Expensive for the health system.

*Cognitive interventions*

The first cognitive interventions were mainly offered to patients with AD (Grandmaison and Simard, 2003; Huckans et al., 2013; Huntley, Gould, Liu, Smith, and Howard, 2015),but more and more cognitive training programs are now being tested in PD. The rationale behind these interventions is relatively the same as for AD, i.e., they would increase the cognitive reserve of the individual (Hindle, Martyr and Clare, 2014; Poletti et al., 2011a) and improve brain plasticity (Poletti et al., 2011a). This may therefore help slow the decline of cognitive symptoms in PD (Hindle et al., 2013; Hindle, Martyr and Clare, 2014). The principle of cognitive reserve is defined as the ability to optimize a person's performance by effectively using brain connections (Stern, 2002). Brain plasticity would be a property that would allow the brain to compensate for structural deficits through other structures that are not damaged, which would normalize the performance of an individual with cognitive dysfunction (Dirnberger and Jahanshahi, 2013; Poletti et al., 2011a). There are currently three types of cognitive interventions: cognitive stimulation, cognitive training and cognitive rehabilitation. Also, a few rare studies have attempted to combine cognitive intervention with exercise, but the results have not shown a change on a global scale of cognition (Reuter et al., 2012).

Cognitive stimulation

Cognitive stimulation is an intervention conducted individually or in small groups, usually in the form of games or discussions. The goal is to encourage reflection and mental activation. These interventions are therefore not adapted to the specific deficits of the patient and the desired effect on cognition is rather global(Woods, Aguirre, Spector and Orrell,2012; Huntley et al., 2015). In PD, only one study looked at cognitive stimulation (Nombela et al., 2011). For six months, 10 health checks and 10 PD patients reporting subjective cognitive complaints had to fill out a small sudoku and meet with a psychologist once a week for correction. The group of PD patients was separated into two: five patients completed the program with sudoku and the other five did not perform any activity. The results showed that stroop test performance was improved in sudoku-trained PD patients and that brain activation measured by functional magnetic resonance imaging(fMRI) was lower in the experimental group compared with control Parkinson's patients, so the task required fewer resources in the trained group compared to the control group. The authors concluded that the proposed exercises optimized patients' cognitive resources (Nombela et al., 2011). However, the skills acquired by patients could not be transferred to other cognitive domains or AIVQs, and no measures were planned for quality of life, which are the main limitations of this study.

Cognitive rehabilitation

It is an individualized approach, centered on the needs of the patient. Cognitive rehabilitation can be used in patients with mild and moderate dementia (Thivierge, Simard, Jean, and Grandmaison, 2008). This type of intervention can be done in the patient's living environment and target concrete tasks, focused on AIVQs, but can also be administered in a hospital setting. Two types of laboratory-validated cognitive strategies or techniques are generally used: compensatory strategies and restorative strategies (Buschert, Bokde, and Hampel, 2010). The former aim to learn a new way of accomplishing a task, while the latter aim to restore cognitive deficit functioning using cognitive techniques developed in the laboratory (Buschert et al., 2010). There are few studies with a rigorous methodological design, but pilot studies with patients with AD (mild to moderate stages) show possible benefits (Clare et al., 2010; Thivierge et al., 2008; Thivierge et al., 2014). In PD, no studies have been carried out to date on this type ofintervention.

Cognitive training

Cognitive training aims to maintain or improve certain cognitive functions through the repeated practice of tasks most often on a computer, with cognitive techniques validated in the laboratory. Generally, training sessions are done in small groups. In PD patients, with no regard for cognitive status, the majority of cognitive intervention studies focus on cognitive training techniques, but the methodologies used are highly variable, which restricts the interpretation of the results (Angelucci et al., 2015; Edwards et al., 2013; Milman et al., 2014; Mohlman et al., 2011; París et al., 2011; Peña et al., 2014; Petrelli et al., 2014; Sammer et al., 2006; Zimmermann et al., 2014). A first meta-analysis on PD patients, regardless of their cognitive status(Calleo, Burrows, Levin, Marsh, Lai and York,2012), included four studies looking at cognitive training programs. On the other hand, given the small number of participants in the included studies (n varying between 14 and 33 depending on the study) and the variability of the different training programs, in terms of session frequency, duration of sessions and content of training programs, no quantitative analysis was performed. The authors cannot conclude that cognitive training programs are effective, but find that they are feasible and tolerable (Calleo et al., 2012).

A second meta-analysis of 2013 on the subject this time showed that studies on cognitive training with a rigorous experimental design showed positive results, but only one study with randomized design – control is not sufficient evidence to conclude that there are or no benefits on cognition (Hindle et al., 2013; París et al., 2011) . Moreover, no study has shown improvement on a measure of overall cognitive functioning (e.g.MMSE).

A recenter meta-analysis(Leung, Walton, Hallock, Lewis, Valenzuela and Lampit,2015) calculated an effect size for the results of the seven studies it included. Hedges' *g* was estimated to be *g* = 0.23, *p* = 0.037, reflecting a small but significant effect size. On the other hand, the cognitive status of the participants is not described, so they could be MP patients without TCL, MP-TCL or MP with dementia. The few cognitive training studies in PD will be described more precisely, as each was analyzed by one of the three meta-analyses mentioned above.

*PD patients without cognitive impairment, or with subjective cognitive complaints.* A few studies show interesting results in patients with PD without objective cognitive impairment, but in some cases complain of cognitive difficulties. First, the case series study of Mohlman et al. (2011) sought to validate the feasibility and tolerability of a computerized cognitive training program, the "Attention*Process Training",*which iscompleted in four 90-minute sessions over four weeks. They administered this program to 16 PD patients without cognitive impairment, but did not follow up after the immediate post-workout measurement. The results did not mention any significant cognitive improvement, but the tolerable criteria were generally positive in terms of fatigue experienced by patients, effort during training sessions, amusement felt, and perception of progress. These constructs were evaluated at the end of each week via a specific sheet included in the program material and which contained questions with a Likert-type scale for each construct. The conclusion of this study is therefore that this program is well tolerated and feasible with pd patients, but more studies are needed to confirm the improvement in cognition (Mohlman et al., 2011).

The study by Zimmerman et al. (2014) attempted to compare two training programs: a first group of patients received a computerized program specific to cognition (functions of attention, working memory and planning) and a second group received a program that was not specific to cognition, but which involved a motor component (i.e. playing the Nintendo Wii console). All patients participated in three supervised sessions of 40 minutes per week, for 4 weeks, but no follow-up assessment was performed after the immediate post-workout measurement. The results showed no significant difference between the groups at the end of training for any of the cognitive domains assessed, although there was an improvement in the Wii-trained group between pre- and post-intervention attention measurement*("Test of Attentional Performance").* The authors conclude that the program with the Wii is less expensive and would amuse patients more than cognitive training computer software, especially since the cognitive benefits would be similar between the two approaches.

Finally, the randomized-active control study of Pena et al. (2014) tested a "paper-pencil tasks" cognitive training program, adapted to memory, attention and executive functions, which lasted 12 weeks, at the rate of three sessions of 60 minutes per week. The authors separated 42 participants into two groups: 20 participants followed the program and 22 participants did group activities (reading, drawings, maps, etc.). The results show a significant intergroup improvement in favor of the experimental group for the speed of information processing, as measured by tmT – A, of verbal memory, measured by the*"Hopkins Verbal Learning Test"*and visual memory, as measured by the "BriefVisual Memory*Test".* ». In addition, significant improvements were noted in theory of mind, as measured by the (cognitive)-type Task of Happé. There were also improvements in functional deficits, as measured by the short version of the WorldHealth Organization*Disability Assessment Schedule*questionnaire. At the cognitive level, the same tests were usedin pre-intervention and post-intervention, so it could be that a practice effect contributed to the participants' improvement on these measures. Again, too, the authors did not conduct a follow-up assessment after immediate post-intervention measures with participants.

In summary, several different training programs have been evaluated in Parkinson's patients without cognitive impairment and the methodologies are also very different. The programs were well tolerated by participants; very few adverse effects have been reported. The latest study published to date on the subject is that of Petrelli et al. (2015). This is the study with the longest follow-up to date (one year after the end of training). The authors compared two training programs; one is structured and the other unstructured, with a control group (passive, waiting list type). Their sample consisted of MP patients without MCI: 24 participants in the structured training group (group 1), 24 participants in the unstructured training group (group 2) and 21 participants in the control group (group 3). Cognitive training consisted of 12 90-minute sessions spread over six weeks. For group 1, these were structured exercises targeting specific cognitive functions (memory, attention, executive functions, without further details from the authors), coupled with psychoeducation sessions on these functions. For group 2, the training consisted of small unstructured computerized exercises (e.g. mainly games), without psychoeducation. Their pre- and post-intervention efficacy measures were outcomes at global cognition scales (MMSE and Dem-Tec) as well as subsequent or non-development of LCI. The results show that even one year after the end of the cognitive training program, the risk of developing MCI was halved for participants in both trained groups. In terms of cognitive measurement results, the two training programs did not increase performance at MMSE, but only the performance of participants in the structured program did not decline. Conversely, that of participants in the other two groups declined over time. Thus, even a year after the end of training, patients still benefit from the gains they had made, especially in terms of preventing cognitive decline.

*PD patients, without specifying cognitive status.* Other studies included participants with cognitive impairment, but did not specify whether they had MCI or Parkinson's dementia. The threshold criterion for inclusion of participants in the studies was set at a minimum score of 24 or 27 on the MMSE. The first study published in this section is that of Sammer et al. (2006) (average MMSE score of 27.15). They recruited 26 PD patients who were hospitalized and divided them into two groups: 12 patients received cognitive training during their hospital stay while 14 other patients did not. The cognitive training consisted of 10 sessions of 30 minutes of activities such as puzzle tasks (from WAIS-IV) or exercises stimulating executive functions (e.g. repetition of thefollowing*"Behavioral Assessment Dysexecutive Syndrome"*(BADS) battery tasks: time estimation, "Zoo*Map Test* and key search). The results show that the trained group improved more than the control group on other tasks of the BADS battery than those mentioned above measuring the executive functions,in particular the task of the sixelements, although the control group also improved. No measurement of the transfer of learning in daily life has been made. Following immediate post-intervention action, participants were not followed (Sammer et al., 2006).

The study by Edwards et al. (2013) evaluated a computerized program focused on the speed of information processing, which totaled 20 hours spread over 12 weeks, which participants completed themselves without the supervision of a therapist, according to their schedule. The study included 74 participants divided into two groups: 32 participants in the trained group and 42 in the passive control group. The primary efficiency measure is the*"Useful Field of View"*task, a task estimating the speed of information processing. The results show a significant improvement in this measure between pre- and post-workout measurement, but there is no significant difference in overall cognitive functioning as measured by theSelf-reportedCognitive Self*Report*and depressive symptoms as measured by the Center for Epidemiological Studies Depressive*Scale*(CES-D). This suggests that the effects of training will not betransferable to daily functioning as assessed by the self-reported questionnaire (Edwards et al., 2013).

In another published study with quasi-experimental design (a single group of 18 PD patients with pre- and post-intervention measures), Milman et al. (2014) also evaluated a computer training program. The latter was adapted to the base for adult patients with attention deficit disorder (ADD). This program focuses on executive functions and is complemented by three 30-minute sessions per week for 12 weeks. Executive functions were measured with computerized versions of classic tasks such as the Stroop test and a "Go – No Go" paradigm. The results show a significant improvement in participants between pre- and post-intervention assessments of these tasks, but also an improvement in the speed of execution of computerized tasks. In addition, this study conducted a follow-up evaluation four weeks after the post-intervention measure, which showed a subsequent improvement in the tasks measured, compared to the results of the post-intervention measure. Participants had not continued the training program exercises between the end of the intervention and follow-up.

*MP-TCL patients.* More recent studies of cognitive training in PD use the criteria of Litvan et al. (2012) to include MP-TCL patients. Generally, no distinction was made between amnesic or non-amnesic TCLs: all subtypes of MCI were included. Obviously, this can directly influence the results following training, since several programs targeted specific cognitive functions. Thus, it is possible that some programs resulted in functions that were intact in participants, which could lead to a ceiling effect, without really helping the patient. First,Angelucci et al. (2015) were interested in the physiological effects of cognitive training in MP-TCL patients. In addition to measuring the effects on cognition via the ZooMap*Test,*the authors also measured levels of theBrain*Derived Neurotrophic Factor*(BDNF) in the blood, an indicator of neuronal growth. The research sample included 15 participants: seven in the trained group and eight in an "active control" group (exercises not related to executive functions, e.g. dictations). Cognitive training consisted of a series of exercises targeting alternating and problem-solving skills. For both groups, 45-minute meetings were held three times a week, for four weeks. Their results show a significant improvement in the performance of the ZooMap*Test*as well as an increase inblood BDNF levels for the experimental group compared to the control group, although no correlation between these two variables could be established. The authors still conclude that blood BDNF is a good biomarker of cognitive improvement following a training program (Angelucci et al., 2015). No follow-up data after immediate post-intervention evaluation were measured.

The study by Cerasa et al. (2014) attempted to test whether a cognitive training program made changes to fMRI measurements. Their sample consisted of 15 MP-TCL patients: eight participants in the trained group and seven participants in an active control group, who completed a computer program at home. To be included in the study, patients had to have at least one cognitive deficit in attention, information processing speed, or executive functions, but subtypes of MCI were not described in more detail. Cognitive training took the form of two one-hour meetings per week, lasting six weeks. These were computerized activities focused on attention and information processing, which corresponded to the cognitive profile of the participants and therefore targeted their needs. The activities of the active control group consisted of computer task sessions focused on visuomotor coordination and were of the same duration as the activities of the training program focused on attention and speed of information processing. Before and after training, a comprehensive neuropsychological assessment battery was completed in addition to a resting fMRI measurement. The results show that the training condition improved performance on the Empan location and WAIS-IV Codes subtests only for the experimental group. In addition, an increase in functional activity was observed in the superior parietal cortex and the dorsolateral prefrontal cortex in patients in the experimental group, associated according to the authors with attention and executive control, respectively. Their conclusion is therefore that a cognitive training program would promote brain plasticity (Cerasa et al., 2014).

Another published study (Costa et al., 2014) compared cognitive training focused on alternating skills (executive functions) with non-specific training focused on language skills. Thesampleconsisted of 17 MP-TCL patients: nine participants completed the cognitive program and eight completed the language program. The pre- and post-intervention neuropsychological measures were the TMT and the verbal fluency test, alternating condition, of the battery*"Delis and Kaplan Executive Function System"*(D-KEFS). The results show that the group trained on executive functions significantly improved its performance at these two measures following training, which was not the case for the control group trainedwith language exercises.

In the meta-analysis of Hindle et al. (2013) and that of Leung et al. (2015), only one study had superior methodological quality: the study by Paris et al. (2011). This is a randomized design study – double-blind control to test a cognitive training program. The latter is based on computerized tasks and paper-and-pencil tasks, at the rate of three sessions of 45 minutes per week, for four weeks. The study sample consisted of 28 participants separated into two groups: a trained group (n = 16) and an active control group (n = 12). Patients included in the study were evaluated with an exhaustive neuropsychological battery to characterize TCL, a posteriori (before the publication of the criteria of Litvan et al., 2012). The control group received group language therapy sessions of the same duration as the intervention in the experimental group. The majority of participants were PD patients with no significant cognitive impairment. However, eight participants in the trained group and six in the control group met the TCL criteria based on the results of thecomprehensive neuropsychological assessment. The results show a significant improvement following training in measures of attention (SEQUENCES of WAIS-III digits), speed of information processing (WAIS-III codes; Stroop words; TMT-A), memory (CVLT), visuo-spatial and visuo-constructive skills (Rey's complex figure), semantic fluidity (production of animal names) and executive functions (Tower of London; Stroop interference; TMT-B). It should be noted that the study does not mention the use of alternative versions of the tests, so it could be that a practice effect partly explains the results. In addition, there was no improvement in quality of life and achievement of AIVQs. No follow-up evaluation was made following the post-training measure.

Limitations of cognitive interventions

In summary, in PD patients, cognitive intervention studies appear promising, but several methodological limitations prevent the generalization of results (Hindle et al., 2013). First, none of the programs were particularly suitable for the PD population: for the most part, the programs were computerized and could be used with other issues, such as patients with ADHD or AD. Thus, training programs do not seem specific for patients whose cognitive profile is particularly heterogeneous. A program that takes into account and targets the different disorders of patients would probably be more effective. To this end, it is important to remember that studies including MP-TCL patients had, for the most part, not characterized the type of MCI in their participants, but programs specific to certain cognitive functions were nevertheless used;therefore, it is possible that intact functions were trained while deficit functions could have been ignored. . This could therefore partly explain the modest results that have been presented previously.

Also, only two studies performed a follow-up assessment of several weeks after the participants' post-intervention measurement: one study whose follow-up was approximately 4 weeks after the end of training (Milman et al., 2014) and the other, one year later (Petrelli et al., 2015). So there is very little data on long-term follow-up, although the results look promising at present. A follow-up evaluation of several weeks after the end of the intervention would make it possible to check whether the gains are maintained after the end of the training and whether cognitive decline can be prevented or slowed down.

In addition, the main purpose of these studies was mainly to improve the cognitive functions of patients as measured by so-called "laboratory" cognitive tests. It was therefore not shown that the different programs actually helped patients to improve the performance of daily tasks, such as AIVQs. In fact, none of the studies systematically assessed the achievement of AIVQ in participants.

Still related to neuropsychological tasks, few studies reported whether they used the same measures in pre- and post-intervention, which could cause a practice effect in participants, especially given the short time interval between pre- and post-intervention assessments (on average, four weeks). Incidentally, the literature reports that a practice effect may occur if the same tests are re-administered six months or less apart (McCaffrey et al., 1993). Very few cognitive training studies have reported the use of parallel versions of neuropsychological tests, which could have decreased a possible practice effect (Angelucci et al., 2014).

For other important measures to include, psychological and behavioural symptoms (SSC) may affect the effectiveness of the cognitive intervention. For example, difficulty completing tasks could lead to additional distress in some patients. It would therefore be important to document effects on SCPs, in the same way as side effects of pharmacological treatment. Conversely, completing a training program could improve CPS if the patient feels more efficient on a daily basis. With the currently available data, it is not possible to confirm the impact of cognitive training on CPS. In the studies presented above, most patients had very few depressive symptoms at baseline, since the presence of a psychiatric diagnosis was frequently an exclusion criterion. Thus, an improvement would be more difficult to find given a ceiling effect. A majority of studies included only one measure of depression, not a comprehensive assessment of SSC. That said, none of the studies reported deterioration in SPC,although depressive symptoms were more often assessed. Regarding SPC other than depression, very little data is detailed in the studies. For example, two studies assessed anxiety, but none of the programs improved these symptoms (Cerasa et al., 2014; Pena et al., 2014). In addition, no studies have measured caregiver burden, while PD patients often need help on a daily basis. With a computerized training program, little or not adapted to the cognitive difficulties of patients, it would be surprising to get a change for the patient or his caregiver. With a more tailored program, it would be more likely to see an improvement in the caregiver's sense of burden.

In terms of program content, a majority of studies focus on cognitive training programs, which for the most part lack transferability to activities of daily living. Indeed, only the activities performed during training seem to be improved, without the activities of everyday life being particularly documented. Although several programs targeted executive functions, very few details were given as to the precise executive functions trained, by whatmechanisms, with what cognitive techniques these functions were trained, and how the level of difficulty was adjusted according to the performance of each participant as the training evolved. Since executive functions are often impaired in MCI and are often the primary focus of patients' cognitive complaints (Sollinger et al., 2010; Tröster, 2011), it would be wise to specifically target an improvement in these functions to help patients function better in their daily lives(Dirnberger and Jahanshahi, 2013). A rigorous baseline assessment of patients in order to properly characterize the MCI is therefore important, in order to really adjust the program according to their needs. Training programs could thus be better adapted to MP-TCL patients.

Le Goal Management Training

Unlike the training programs mentioned above, *Goal Management Training* (GMT) is at the crossroads between cognitive training and cognitive rehabilitation. It is a program specifically targeting executive functions and tailored to the needs of the patient, although it can be accomplished in small groups (Levine, Robertson, Clare, Carter, Hong, Wilson et al. , 2000) . Indeed, throughout the program, the patient is led to become aware of his difficulties and to develop concrete solutions with the help of the intervener. One of its major advantages is that GMT is based on a well-known theoretical model of executive functions, Duncan's theory of disorganization of behavior following frontal lobe injury or dysfunction (Duncan, 1986; Robertson, 1996).

The purpose of the GMT is to help the patient become aware of what they are doing in the present moment in order to help them effectively perform AIVQ (Robertson, 1996; Levine et al., 2000). In its original form, the participant is taught five steps, such as a routine, to help structure their behaviours: 1) learn to stop to check what they are doing through mindfulness techniques; (2) formulate the main purpose of its current action; (3) dividing tasks into sub-objectives; 4) learn the sub-objectives and encode them; 5) compare the actions undertaken with the goals set and check whether there is agreement. The GMT is done in the form of individualized meetings or in small groups. The original 5-step program is divided into nine modules that take about 90 to 120 minutes each to administer. It is advised by the authors to do one module per week. After each module, participants must complete a few exercises at home, such as writing down in a chart the mistakes made and successes experienced during the past week. Other exercises, such as the body scanning technique, are directly inspired by the movement of mindfulness and allow the participant to center themselves in the present moment.

Several studies have shown that GMT is effective and can be applied to real-world situations experienced by patients (Levine et al., 2000; Levine, Stuss, Winocur, Binns, Fahy, Mandic, et al., 2007; Levine, Schweizer, O'Connor, Turner, Gillingham, Stuss et al., 2011; Schweizer, Levine, Rewilak, O'Connor, Turner, Alexander et al. , 2008; van Hooren, Valentijn and Bosma,2007). Several case studies have been conducted in patients withcerebral cranial trautisms(Levine et al., 2000), cerebellar lesions (Schweizer et al., 2008) or with *spina bifida* disease (Stubberud, Langenbahn, Levine, Stanghelle, and Schanke, 2013). All of these pathologies, such as PD, include deficits affecting the functioning of the frontal lobe, essentially the structure that underlies the performance of executive functions. A few randomised design control studies were also conducted and showed interesting results.

A randomized-control study was conducted in 2007 in 49 elderly patients who were healthy but had cognitive complaints. It is interesting to note that elderly patients, even without objective cognitive impairment, also have frontal lobe degeneration, although it is much less marked than in pathological populations (Levine et al., 2007). After the end of the GMT, patients had improved on several ecological tasks measuring executive functions, the*"Simulated Real Life*Tasks". In addition, their self-reported competence for these tasks, as measured by questionnaires, was also improved, showing that patients felt better equipped to manage their daily activities (Levine et al., 2007). Improvements continued three months after the end of the intervention.

These results were replicated in another sample of 69 elderly people with cognitive complaints (van Hoorren et al., 2007). The GMT group consisted of 38 participants, and the passive waitlist control group included 31. In this study, measures of effectiveness mainly included a self-reported questionnaire on perceived cognitive impairment, the "Dysexcutive (DEX)  *questionnaire",*the Depression and Anxiety subscales of the "SymptomChecklist –*90 items",*as well as performance on the Stroop test. The results show that participants who completed the GMT perceived that they had fewer cognitive problems and that they had fewer anxiety and depressive symptoms. On the other hand, there was no significant difference between the pre-GMT measurement and the post-GMT measurement on performance in the Stroop test. However, stroop measures inhibition abilities, and is therefore not an organizational task. Therefore, an improvement to this task would have been unlikely,since the GMTmainly targets the improvement of organizational and planningstrategies.

Another study was conducted in 19 patients with frontal lesions following a stroke. This time, two cognitive tasks were improved following the GMT: the*"Sustained Attention to Response Task",*a "Go – No Go" task, and the "Tower*Test",*described in the study as a complex visuospatial construction task, but frequently associated with a measure of executive functions (e.g. planning). However, no improvement was observed in the self-reported assessment. However, the authors note that their sample was very heterogeneous in terms of frontal impairment, explaining the wide variability of the results as assessed by the self-reported measures (Levine et al., 2011).

In summary, the GMT is well adapted and validated with different populations with executive deficits caused by frontal alterations. However, it seems important to note some limitations of the GMT. First, the program is available in English only and has not been officially translated for the Francophone population. Also, it is a busy program, as it includes many discussions and exercises that require great attention from the participant. This could therefore be heavy for patients with fatigability on a daily basis. Among other things, exercises that need to be completed at home may be perceived as too time-consuming and effort-intensive required by some patients. In addition, this program was not designed for people with motor difficulties such as those associated with PD. For example, many boards have to be filled out by hand and some exercises require handing out playing cards, which could cause additional motor fatigue. However, in some MP-TCL patients, executive deficits can be explained by alterations at the frontal level, regions interconnected closely to the basal ganglia, and whose functioning is severely affected from the onset of PD. An intervention like GMT may therefore be well suited to PD patients with executive problems and could eventually help them function better in activities of daily living.

**General objectives and assumptions of the project**

**The general objectives** of this project are to translate the GMT administration manual and materials into French, to adapt it to the Parkinson's population, then to verify its tolerability (fatigue, SPC, changes in medication or dosage and caregiver burden) and its effectiveness in PD patients with MCI. Two main steps will be used to achieve these objectives: (1) a multi-baseline case study for the first three objectives and (2) a randomized design-control study to validate the last. For the randomized control study, the effectiveness of the program will be evaluated on executive functions and AIVQs. Secondary objectives will be to assess SSC, quality of life of PD patients, overall cognition and caregiver burden. A final objective will be to explore the relationship between the functioning of theory of mind before the start of the GMT and the improvement of executive functions and AIVQs after the intervention.

**Thegeneral assumptions** are that GMT **1)** will improve executive functions as measured by self- and hetero-reported questionnaires as well as by an ecological task that will be described in the next sections. In addition, since the GMT is more concrete than a cognitive training program, two more exploratory objectives are to see if the GMT allows **2)**  to improve or maintain SPC as measured by the*Neuropsychiatric Inventory*(NPI), overall cognition as measured with the DementiaRating Scale –*2*(DRS-2), as well as the achievement of AIVQ and quality of life as measured by the "  *Parkinson Disease Questionnaire – 39 items"*(PDQ-39). With respect to caregiver burden, there could be  **3)**  either an improvement in burden or no change, as measured by the Zarit*Burden Interview – 12 items*(ZBI-12). Finally, the final hypothesis is that **4)**  the theory of mind capability of MP-TCL patients, as measured by the Faux Pas task at baseline assessment, will be a predictor of improvement in post-GMT executive functions.

**Study 1: Methodology**

*Objectives and assumptions*

The objectives of the first study are **to 1)** translate the administration material and content of the GMT, **2)** adapt it specifically to MP patients, **3)** verify whether GMT is well tolerated and safe for MP-TCL patients, and **4)** test whether the modifications are effective for the target population. The final objective will be **5)** to explore the relationship between theory of mind capacity and the success of the GMT.

The assumptions are therefore that **1)** the GMT will be well tolerated, so that participants will not describe themselves as too tired (according to a self-reported assessment conducted at the end of each session), that there will be no change in their medications (antiparkinsonians, antidepressants, or other psychotropic medication),that there will be a reduction or maintenance of SPC as reported by a caregiver, and an improvement or maintenance of the caregiver's sense of burden. **2)** The modified GMT will allow participants to improve their executive functions, the achievement of AIVQs, their quality of life as well as to improve or maintain their overall cognition. Finally,  **3)** theory of mind capability, as measured during the baseline assessment, will be a good predictor of the effectiveness of the GMT.

*Experimental design*

A multi-level baseline case study will be the quote used. The principle is that each participant must obtain stable results on baseline assessment measures *before* starting the intervention (Levine and Downey-Lamb, 2005). A first participant starts the intervention while a second participant continues the baseline assessments; then eventually, the second participant also receives the intervention. Thus, it is possible to better control for individual differences between participants and to improve the internal validity of the study by showing that a pre-post change in measures can likely be better explained by the intervention than by elements external to the intervention (Hawkins, Sanson-Fisher, Shakeshaft, D'Este, and Green, 2007; Levine and Downey-Lamb, 2005).

*Recruits*

Since a first study that took place in the laboratory focused on the cognitive profile of PD patients, the Ph.D. student will look at the records of the participants of this first study who signed a clause of the consent form in order to be contacted again for further research (approval number PEJ-703, Maxime Doiron). If participants are missing, the others will be recruited at the Neurological Clinic for Movement Disorders (Department of Neurological Sciences, CHU – Pavillon Enfant-Jésus) by Dr. Nicolas Dupré and Dr. Mélanie Langlois, neurologists. The latter, during clinical consultations, will identify individuals who may meet the inclusion criteria and offer them to be part of the research project, including by providing them with a fact sheet on the study. They will ask for their consent so that their contact information is transferred to the Ph.D candidate in accordance with the rules of confidentiality. She will then contact potential participants by phone to explain more specifically what the objectives of the study and the intervention are. If the person agrees to participate, a first meeting for the signing of the consent forms and the start of the diagnostic assessment will be scheduled.

*Participants*

Two participants will be recruited for the case study based on the following criteria.

Inclusion criteria

1. Diagnosis of PD *Brain Bank* (Hughes et al., 1992).
2. Diagnosis of MP-TCL, a multi-domain or non-amnesiac type with a single or multi-domain domain, but always with the presence of executive dysfunctions (according to a type 2 assessment by Litvan et al., 2012).
   - - Gradual decline in cognitive skills, as observed by the patient, caregiver and/or clinician.
     - Objective cognitive decline, as measured by scores between minus one and minus two standard deviations (but not below two standard deviations) from the mean (standards by age and/or level of education) to at least one test of executive function. There may be a performance between minus one and minus two standard deviations from the mean on other tests measuring executive functions and/or in another cognitive domain among: 1) attention and working memory, 2) language functions, 3) episodic or semantic memory, 4) visuospatial functions. Two neuropsychological tasks per domain will need to be performed to confirm the objective cognitive impairment of the participant (neuropsychological assessment criterion type II, Litvan et al., 2012).
     - The difficulties do not significantly interfere with basic daily activities, but the patient may experience some difficulties in the AIVQ in the present study.
     - Cases of mild Parkinson's dementia, with a MoCA score between 21 and 26 as established by the studies of Dalyrimple-Alford et al. (2010) and Robben et al. (2010), will be accepted in the event that there are not enough MP-TCL patients.
3. Presence of a caregiver ready to participate in activities with the patient and complete questionnaires.
4. Antiparkinsonian medication (L-DOPA or other) stable for at least 2 months.
5. Psychotropic and/or nootropic medication stable for at least 3 months.

Exclusion criteria

1. Diagnosis of Parkinson's dementia or a MoCA score below 21(Dalyrimple-Alford et al., 2010; Robben et al., 2010).
2. Diagnosis of another neurological disorder that can cause cognitive impairment.
3. History or presence of substance abuse.

*Procedure*

All meetings will take place at the participant's home, in the company of the caregiver. These meetings will take place when thepatient's medication is optimal in terms of controlling motor symptoms. In order to determine the ON period, patients and their relatives will be interviewed by the PhD student and meetings will be scheduled accordingly. At the first meeting, all the modalities of the study will be presented in detail and the information and consent forms will be signed by the participant and his/her relative. The PhD student will remain available to answer all questions from participants or their loved ones. Following the obtaining of consent, the diagnostic evaluation can begin. In order not to tire the participant, at least three diagnostic assessment meetings will be scheduled, each lasting a maximum of 60 to 75 minutes. Table 6.1 of Appendix 6 provides an estimate of the time required for each of the diagnostic battery measurements, with a maximum total duration of approximately three hours.

Once the diagnostic assessment has confirmed that the participant meets the inclusion criteria of the study, the administration of the baseline measures can begin. The participant's evaluation will be administered by the Ph.D candidate at pre-arranged meetings. As for the caregiver questionnaires, they will be administered by the student at another time, during a telephone appointment, for the sake of discretion. At least two basic level assessments will be made for the first participant, while at least three basic level assessments will be made for the second participant. This follows standard procedures for multi-level basic quotations (Levine and Downey-Lamb, 2005). In the event that the measures are not sufficiently stable after the number of pre-established meetings, additional baseline assessments will be added as required.

When the results of the baseline measurements are stable, the intervention can begin. It will also take place at the participant's home, once a week, for five weeks, in the presence of the caregiver. One module per week will be covered, and participants will have activities to complete between sessions. The duration of each session may vary (on average 60 minutes). In the event that the patient is too tired, the different sessions of the GMT can be separated into two shorter sessions if necessary. One week after the end of the GMT, post-intervention measures will be administered in the same way as for the assessment of the baseline level.

*Intervention*

The original GMT consists of nine modules administered at the rate of one and a half hours per week, and which aim to learn the steps of a strategy to remain aware of what the patient is doing, without turning away from an end goal (Levine et al., 2000; 2007). In particular, the patient learns the steps of a process in which he must take the time to STOP, FORMULATE his main goal and DIVIDE it into sub-tasks as many times as necessary. However, for practical reasons, some adjustments to the GMT will be made to make it more tolerable for PD patients with motor difficulties and high fatigability. For example, while the original GMT includes many playing card distribution exercises (rule alternation exercises), the modified GMT does not include these exercises. The same applies to some ofthe tables to be filled in by hand inthe participant's manual, the topics of which will instead be discussed in the form of a discussion. The original program has been somewhatcurtailed to be completed in five weeks instead of nine, and each session is a maximum duration of 60 to 75 minutes,instead of 90 to 120. However,the modified GMT will always need to allow patients to learn new strategies to overcome their deficits (Levine et al., 2000; 2007; 2011; Schweizer et al., 2008; Stubberud et al., 2013; van Hoorren et al., 2007).

*Measurements*

Several standardized and validated neuropsychological tasks will be performed for diagnostic assessment to verify inclusion criteria. For baseline and follow-up level measures, questionnaires will be used to check whether the GMT allows for an improvement in executive functions and other concepts assessed. There will also be a neuropsychological measure specific to executivefunctions, possessing a parallel form, and whose two versions (original and parallel) have beenadministered alternately, in order to minimize the effect of practice. Another student in the laboratory, Laïla El-Amrani, will do the post-GMT assessment, so that the final results remain double-blind. The use of self-reported questionnaires provides an overview of the deficits as perceived by the participant, while the questionnaires completed by the caregiver will be able to complete and validate the information obtained. Tables 6.1, 6.2 and 6.3 of Annex 6 provide an estimate of the time required for each of the diagnostic battery measurements and the GMT efficiency measures.

Battery of tests for diagnostic assessment

The battery of tests that will be used for the diagnostic assessment of participants is described in detail in Appendix 4. The tests were selected to meet the MP-TCL type II endpoints according to Litvan et al. (2012).

Questionnaires and tests to measure the tolerability, safety and effectiveness of the GMT

The primary measures chosen will be the tolerability, safety, SSC and caregiver burden measures. Secondary measures will include executive function, quality of life, AIVQ and overall cognition.

*Tolerability and security.* Attendance at the proposed sessions and activities, the level of fatigue experienced at each session (Likert scale of 0-10, see Appendix 5) and changes in medication or medication dosage will be used as measures of tolerability. In addition, the SSC and caregiver burden measures, which will be described in a future section, will also be used to measure the tolerability and safety of the GMT as part of the pilot study.

*Executive functions.* Executive functions will be assessed using self-reported measures by the participants and also by anobjective cognitive measurement.

The*"Dysexecutive* (DEX) *questionnaire"*(Wilson et al., 1996), included in the BADS, contains 20 questions. It is self-reported and concerns disorganizing behaviors that are rated on a scale of 0 to 4 (0 corresponding to "never" and 4 to "almost always") for a maximum possible negative score of 80 (the higher the score, the more problems the person experiences). It has good convergence validity and correlates positively with the total SCORE of the BADS. It is considered an ecological measure, well suited to measure executive deficits experienced by frontal patients (Wilson et al., 1996).

The questionnaire provided in the GMT toolkit (translated by the Ph.D. student) includes 34 questions on frequently made errors that must be rated on a scale of 0 to 10 (0 for "no problem" and 10 for a "very serious problem"). The maximum score is therefore 340 (the higher the score, the more problems the person experiences).

A MEASURE of the BADS has been chosen as an objective measure of executive functions, since it is available in an original version and a parallel version, which will make it possible to take pre-intervention and post-intervention measures avoiding as much as possible a practical effect. Indeed, the*"Zoo Map Test"*measures organizational and planning skills. The participant must establish the route he should follow in a zoo. It may or may not be guided by the speaker. The Zoo*Map Test*has good construct validity and correlation with conventional tasksmeasuring executive functions (Wilson et al., 1996).

*Global cognition.* The*Dementia Rating Scale 2*(DRS-2; Mattis, 2001) is a measure comprising an original form and an alternative form,so that it can beadministered twice to participants, before and after the GMT. Briefly, it is divided into five subscales: attention, initiation / perseverance, construction, concepts and memory; its maximum possible total score (positive) is 144. In addition, there are now Quebec standards for rating it (Lavoie et al., 2013). This tool is considered to have good psychometric properties and can effectively detect cognitive decline in MP-TCL patients and those with Parkinson's dementia (Matteau et al., 2011; 2012).

*Instrumental activities of daily life and quality of life.* The PDQ-39 is a questionnaire specific to the population with PD and in 39 items. It assesses eight dimensions related to quality of life: motor disability (10 items), ADLs (6 items), emotional well-being (6 items), stigma (4 items), emotional support (3 items), cognition (4 items), communication (3 items) and comfort (3 items) (Pirogovskyetal., 2014; Rosenthal et al., 2010). Each item is rated on a scale from "never" (0 points) to "always" (4 points) and all scores (for each scale and total score) are changed as a percentage. The higher the score, the worse the quality of life. This questionnaire is widely used because of its good construct validity, test-retest fidelity and good internal consistency (Peto et al., 1998).

*Executive functions of the participant, rated by a relative.* The DEX (helping version) as well as the questionnaire provided in the GMT kit (helping version containing 25 questions in the same modalities as the self-reported questionnaire,translated by the Ph.D student) will be administered to check if the changes made by the GMT are noticeable to the participant's loved one.

*Caregiver burden.* The ZBI-12 is a self-reported questionnaire measuring caregivers' sense of burden in 12 questions (Bédard et al., 2001; Hébert et al., 2007). The rating is done on a likert scale in five pointss ("never" corresponds to a score of 0 and "always" corresponds to a score of 4). The maximum total score is 48, which corresponds to a feeling of high burden. This is a widely used measure among caregivers, particularly because of good test-retest fidelity (Hébert et al., 2007).

*Cognitive and behavioral symptoms.* NPI-12 (helper and clinician versions) measures different symptoms such as delusions, hallucinations, agitation, dysphoria, anxiety, apathy, irritability, euphoria, disinhibition, motor difficulties, sleep difficulties and problematic eating behaviours (Cummings et al., 1994; De Meideiros et al., 2010). For each symptom, the frequency is rated on a scale of four and the severity is rated on a scale of three. The score for each symptom is the product of frequency and severity, making a maximum of 12 points per symptom. For the total score, the score for each symptom is summed, making a total maximum of 144 (Cummings et al., 1994; De Meideiros et al., 2010). Both versions of the NPI have been validated with the Parkinson's population and show good inter-judge concordance (for the total score: *r* = 0.93; Aarsland et al., 1999).

*Planned statistical analyses*

In order to measure the magnitude of the change between baseline and post-intervention assessment, an effect size measure will be used: the*Common Language Effect Size*(CLES), a measure comparable to Cohen's *d* (McGraw and Wong, 1992) for each of the primary and secondary measures. The CLES makes it possible to calculate an effect size specific to each participant by generating a Z score representing the difference between the baseline means (M_1)_and the post-intervention averages (M2,McGraw and Wong, 1992).

Z_C_ = (M_1_ – M_2_) / √ (Var_1_ – Var_2_)

*Where Var_1_*  *and Var_2_* are the *variances of the pre-*  *and post-intervention means.*

*Changes following Study 1 for the planning of Study 2*

The results of the analyses of study 1 will validate the n required for study 2 by completing a power analysis. The duration of each session has been shortened, but in the event that a duration of 60 minutes is poorly tolerated, the flexible format of the GMT will allow each session to be split into two shorter sessions, of 30 minutes each. In addition, some of the activities to be completed at home could be shortened. However, previous studies on GMT (Levine et al., 2000; 2007) had been conducted with patients with traumatic brain injury, populations whose cognitive fatigue can be highly variable. However, particular attention should be paid to the participants' motor fatigue in the case of this study.

**Study 2: Methodology**

*Objectives and assumptions*

The objectives of study 2 are to verify whether, compared to anactive control condition (alternative interventionprogram), the GMT improves **1)** executive functions as reported by the participant and his relative on the DEX, the questionnaires included in the GMT and the ecological neuropsychological test assessing executive functions between evaluations pre- and post-GMT. In addition, Study 2 will test whether the GMT will lead to:  **2)** an improvement or maintenance in the achievement of AIVQ and in the quality of life as reported bythe participantwith pdq-39 as well as in overall cognition as measured by DRS-2. In the same vein, the GMT could lead to:  **3)** changes in SSC as reported by the clinician and caregiver according to NPI-12; as well as **4)** in the caregiver's sense of burden as reported by ZBI-12.

The assumptions are that **1)** the GMT will once again be well tolerated, that executive functions will be improved after training. Also,  **2)** the achievement of AIVQ, quality of life, overall cognition, SPC and caregiver burden will either be maintained or improved for the GMT group following training.

*Experimental design*

A randomised design – single-blind control with active control group will be used for this study. Participants will be matched according to age, gender, education level and type of MCI, then randomly assigned to one of two groups (n = 5 per group, to be confirmed): group 1, experimental, will follow the GMT for five weeks, at a session of 60 to 75 minutes per week, and group 2 will follow a control program (active) , i.e. psychoeducation sessions on PD and cognitive functions that will be of the same frequency and duration as those of the experimental group.

*Recruitment*

The same method of recruitment as Study 1 will be used, unless there is a major change made during Study1.

*Participants*

This time, ten MP-TCL participants will be recruited (n = 5 per group; to be confirmed). The inclusion and exclusion criteria will be the same as for Study 1.

*Procedure*

The procedure for the initial information and consent meeting will be the same as for Study 1. For diagnostic assessment and baseline measures, the procedure will be the same as for Study 1, except for the number of baselineassessments (for Study 2, only one baseline assessment per participant). Once the administration of the basic level measures is completed, the intervention can begin. It will also take place at the participant's home, once a week, for five weeks, in the presence of the caregiver. One module per week will be covered, and participants will have activities to complete between sessions. The duration of each session may be variable (on average one hour). At the end of the GMT, immediate post-intervention measures will be administered in the same way as for the baseline assessment. To ensure a double blind, another Ph.D student in the laboratory, Laïla El-Amrani, will do the post-intervention and follow-up assessments without knowing which of the two groups each participant has been assigned. With respect to participants, the Ph.D student will explain at the time of signing the consent forms that two different training groups will be formed, but participants will not be informed of the program to which they have been assigned. A follow-up meeting, 12 weeks after the immediate post-intervention measurement, will be conducted.

*Intervention*

Unless changes not be brought to the GMT after the pilot study, the same program as for Study 1 will be Administered. With regard to the condition control active, these will be psychoeducation sessions on PD and cogniti functionsaffected in patients, at the rate of one session one hour per week, during five Weeks. The themes of psychoeducation will be diverse: pgeneral resentation of PD, some brain damage and some disorders associated with PD other qi.e. engines. The psychoeducation session will be 30 minutes, and the rest of the meeting will be devoted to training of full conscience (by ex. breathing exercises and body scan). Psychoeducation is a standard procedure in patients with cognitive impairment (Strauss, Sherman, & Spreen, 2006).

*Measurements*

The same measures as Study 1 will be used for the diagnostic evaluation and effectiveness of the GMT. This time, the primary measures chosen will be the efficiency and AIVQ measures on executive functions. Secondary measures will include measures of tolerability,quality of life, cognition, SSC, and caregiver burden. For the follow-up evaluation, which will take place 12 weeks after the immediate post-GMT evaluation, the same measures that have been administered at the baseline level will be used. Appendix 7 provides a timeline for locating the different measures assessed in Studies 1 and 2.

*Planned statistical analyses*

For each group, for each variable, descriptive statistics will be calculated (mean, standard deviation). Next, a general mixed linear model, to verify the existence of intra- and inter-group differences in pre- and post-intervention measures, will be applied to determine whether the GMT is superior to the psychoeducation/mindfulnessexercise program. The required n will be determined following a power analysis conducted from the results of Study 1. All scans will be conducted with the SPSS software package, version 21.0.

**Ethical considerations**

*Risks and potential side effects*

It may be that some patients are anxious during the neuropsychological assessment, because theywant to perform well. In addition, neuropsychological assessment sessions and GMT sessions may cause fatigue in participants. Since the GMT sessions will be held once a week for five weeks, participants may have scheduling conflicts.

The student tried to answer these possible problems, in particular by adding a question at the end of each module in theparticipant's notebook to find out about the participants' fatigue. If participants aretoo tired to continue a session, the flexible format allowsactivities tobe separated into two or three sessions, if necessary.

*Potential benefits*

Participants will have access to a cognitive training method that could improve their daily lives and promote their autonomy to some extent. They will learn more about their cognitive functioning and help advance knowledge about PD and the executive dysfunctions associated with it. In addition, in the event that a participant has an abnormal result in one of the measures, he or she may be redirected to the appropriate resources.

*Confidentiality*

All data will be confidential and will be encrypted so that the name and contact details of each participant do not appear on any report or computerized data. The data collected during the evaluation will be kept in a file kept under lock and key at the Geriatric Neuropsychology Laboratory of Université Laval for 10 years; they will be safely destroyed following this period. Only the encrypted code assigned to each participant will be used in the computerized database (for statistical analyses) and only this code will appear in the files related to statistical analyses. If the research is published in scientific journals and congresses, no participant will be identified. Only the Ph.D student and the laboratory director will have access to the code to identify participants from their file number. Only the attending physician, the Ph.D student and the director of the laboratory will have access to the nominative data of the participants. Participants may or may not sign a clause in the consent form regarding the further use of their denominalized data for other projects (secondary analyses on a database) of the laboratory. If they are interested, they will have access to publications about their data. Any information that allows them to be recognized directly or indirectly will be removed for the sake of confidentiality.

*Free and informed consent*

Participants are free to participate or not in the research and may withdraw their consent at any time. This procedure is the same for caregivers who agree to participate. Since this research is aimed at competent adult individuals, all scientific and ethical procedures will be explained to them in detail so that their consent is completely free and informed. In case of discomfort or distress, the doctoral student will take all necessary measures to best help the participant. If the participant's discomfort is beyond their competence, the participant and their loved one will be redirected to the appropriate resources. If the participant decides to withdraw consent, there will be no consequences with respect to access to and quality of services received in their health facility. By signing the consent form, participants do not waive any rights and researchers retain their legal and professional responsibilities towards them. However, no financial compensation will be given to participants.

**Potential benefits and conclusion**

Although some medications are effective in delaying the motor symptoms of PD, the effects on cognition in MP-TCL patients remain limited(Roy, Doiron, Talon-Croteau, Dupré and Simard, in preparation). With the aging of the population, it has been estimated that between 8.7 and 9.3 million people will develop PD by 2030 (Dorsey,Constantinescu, Thompson, Biglan, Holloway et al., 2007). Non-pharmacological avenues could be a good complement to medication, often safer and less costly to the health system. In addition, this type of intervention has the potential to concretely improve people's lives, by targeting the enhancement of AIVQs and thereby promoting patient autonomy.

**Schedule**

| **Year** | **Objective** | **Fact?** |
| --- | --- | --- |
| **Year 1 (2015-2016)**  Fall 2015  Winter 2016  Summer 2016 | - Translation of the GMT and questionnaires, and preparation of the multi-base x-level case study (adaptation of the program for Parkinson's patients). - Writing of the project seminar. | X  X |
| **Year 2 (2016-2017)**  Fall 2016  Winter 2017  Summer 2017 | - Presentation of the project seminar to the supervisorycommittee. - Application for ethics approval and preparation of the case study (if corrections to be made during ethics approval). - Multi-baseline case study with two patients, adaptation/modification of GMT, data collection and analysis. |  |
| **Year 3 (2017-2018)**  Fall 2017  Winter 2018  Summer 2018 | - Drafting and submission for publication of Article 1 on the case study. - Preparation of the randomized study – control, ethical approval to be renewed, recruitment, diagnostic evaluation of participants and start of GMT training of participants. - April 2018 at the latest:^1st^ evolutionary seminar. - EnGMT trail of participants. |  |
| **Year 4 (2018-2019)**  Fall 2018  Winter 2019  Summer 2019 | - Continuation and end of the randomized study – control, analysis of results, drafting and submission of article 2. - April 2019 at the latest:^2nd^ evolutionary seminar. - Writing of the general introduction and discussion of the thesis. - Presentation of the seminar authorizing the submission of the thesis and initial deposit of the thesis. |  |
| **Year 5 (2019-2020)**  Autumn 2019, winter2020 and summer2020 | - Boarding school. |  |

**References**

Aarsland D. (2016). Cognitive impairment in Parkinson's disease and dementia with Lewy bodies. *Parkinsonism and Related Disorders*, *22 Suppl 1*, S144–8.

doi:10.1016/j.parkreldis.2015.09.034

Aarsland D, Bronnick K, Alves G, Tysnes OB, Pedersen KF, Ehrt U, and Larsen JP. (2009). The spectrum of neuropsychiatric symptoms in patients with early untreated Parkinson's disease. *Journal of Neurology, Neurosurgery, and Psychiatry,* *80*(8), 928–930.

doi:10.1136/jnnp.2008.166959

Aarsland D, Bronnick K, and Fladby T. (2011). Mild cognitive impairment in Parkinson's disease. *Current Neurology and Neuroscience Reports,* *11*(4), 371–378.

doi:10.1007/s11910-011-0203-1

Aarsland D, Larsen JP, Lim NG, Janvin C Karlsen K, Tandberg E and Cummings JL. (1999). Range of neuropsychiatric disturbances in patients with Parkinson's disease. *Journal of Neurology Neurosurgery and psychiatry;*67, 492-496.

Adler, CH, and Thorpy, MJ. (2005). Sleep issues in Parkinson's disease. *Neurology*, 64(12 suppl 3), S12-S20.

Alcaro A, Huber R, Panksepp J. (2007). Behavioral functions of the mesolimbic dopaminergic system: an affective neuroethological perspective. *Brain Res Rev*. 56, 283-321.

Anderson JR. (1982). Acquisition of cognitive skill. *Psychological review*, 89(4), 369.

Anderson JR., and Schunn C. (2000). Implications of the ACT-R learning theory: No magic bullets. Advances in instructional psychology, *Educational design and cognitive science*,1-33.

Angelucci F, Peppe A, Carlesimo GA, Serafini F, Zabberoni S, Barban F, et al. (2015). A pilot study on the effect of cognitive training on BDNF serum levels in individuals with Parkinson's disease. *Frontiers Human Neuroscience*, *9*,130. doi:10.3389/fnhum.2015.00130

Baddeley A. (2010). Working memory. *Current Biology,*20(4), R136-R140.

Baddeley A, Sala SD, Papagno C, and Spinnler H. (1997). Dual-task performance in dysexecutive and nondysexecutive patients with a frontal lesion. *Neuropsychology*, Vol 11(2), 187-194.

doi: [http://dx.doi.org/10.1037/0894-4105.11.2.187](http://psycnet.apa.org/doi/10.1037/0894-4105.11.2.187)

BarkerRA, and Williams-Gray CH. (2015). Mild Cognitive Impairment and Parkinson's Disease-Something to Remember. *Journal of Parkinson's disease*, 4(4), 651-656.

Bastiaanse R and Lenders KL. (2009). Language and Parkinson's disease. *Cortex;*25, 912-914.

Bédard M, Molloy W, Squire L, Dubois S, Lever JA, and O'Donnell M. (2001). The Zarit Burden Interview : a new short version and screening version. *Gerontologist;* 41(5), 652-657.

Bell-McGinty S, PodellK, Franzen M, Baird AD, and Williams MJ. (2002). Standard measures of execu- tive function in predicting instrumental activities of daily living in older adults. *International Journal of Geriatric Psychiatry*, 17(9), 828-834.

Benedict, RHB, Schretlen D, Groninger L, Dobraski M, and Shpritz B. (1996). Revision of the Brief Visuospatial Memory Test: Studies of normal performance, reliability, and validity. *Psychological Assessment,* 8(2), Jun 1996, 145-153.

Benton AL (1992). *Benton Visual Retention Test – revised*  (5th ed.). San Antonio: The Psychological Corporation.

Benton AL (1994). *Contributions to neuropsychological assessment: A clinical manual*. Oxford University Press, USA.

Boller F, Passafiume D, Keefe NC, Rogers K, Morrow L, and Kim Y. (1984). Visual Oddities in Parkinson's disease: role of perceptual and motor factors. *Archives of Neurology;*41, 485-490.

Bott NT, Johnson ET, Schuff N, Galifianakis N, Subas T, Pollock J, et al. (2014). Sensitive measures of executive dysfunction in non-demented Parkinson's disease. *Parkinsonism and Related Disorders*, *20*(12), 1430–1433. doi:10.1016/j.parkreldis.2014.10.007

Bora E, WalterfangM, and Velakoulis D. (2015). Theory of mind in Parkinson's disease: A meta-analysis. *Behavioural Brain Research*, 292, 515-520.

Braak H, Del Tredici K, Rüb U, de Vos RAI, Jansen Steur ENH and Braak E. (2003). Staging of brain pathology related to sporadic Parkinson's disease. *Neurobiology of Aging,* *24(2),*197–211.

Bronnick K, Alves G, Aarsland D, Tysnes OB and Larsen JP. (2011). Verbal memory in drug naive, newly diagnosed Parkinson's disease: The retrieval hypothesis revisdited. *Neuropsychology,*25(1), 114-124.

Buschert V, Bokde ALW, and Hampel H. (2010). Cognitive intervention in Alzheimer's disease. *Nature,* *6*(9), 508–517.

doi:10.1038/nrneurol.2010.113

Buschke H. (1984). Cued recall in amnesia. Journal of Clinical Neuropsychology, 6(4), 433-440. doi: 10.1080/01688638408401 233

Cahn DA, Sullivan EV, Shear PK, Pfefferbaum A, Heit G, Silverberg G. (1998). Differential contributions of cognitive and motor component processes to physical and instrumental activities of daily living in Parkinson's disease. *Arch Clin Neuropsychol*, 13(7), 575-583.

Calleo J, Burrows C, Levin H, Marsh L, LaiE, and York MK. (2012). Cognitive rehabilitation for executive dysfunction in Parkinson's disease: application and current directions. *Parkinson's Disease*.

Cerasa A, Gioia MC, Salsone M, Donzuso G, Chiriaco C, Realmuto S, et al. (2014). Neurofunctional correlates of attention rehabilitation in Parkinson's disease: an explorative study. *Neurological*Sciences,  *35(8),*1173–1180.

doi:10.1007/s10072-014-1666-z

Chaudhuri KR, and Schapira AH. (2009). Non-motor symptoms of Parkinson's disease: dopaminergic pathophysiology and treatment. *Lancet Neurol*. 8, 464-74.

Cheewakriengkrai L, and Gauthier S. (2013). A 10-year perspective on donepezil. *Expert Opinion on Pharmacotherapy,* *14(3),*331–338.

doi:10.1517/14656566.2013.760543

Cicchetti F, Drouin-Ouellet J, and Gross RE. (2009). Environmental toxins and Parkinson's disease: what have we learned from pesticide-induced animal models? *Trends in Pharmacological Sciences*, *30*(9), 475–483. doi:10.1016/j.tips.2009.06.005

Choi J, and Twamley EW. (2013). Cognitive rehabilitation therapies for Alzheimer's disease: a review of methods to improve treatment engagement and self-efficacy. *Neuropsychology review,* 23(1),48-62.

Claassen DO, Josephs KA, Ahlskog JE, Silber MH, Tippmann-Peikert M, Boeve BF. (2010). REM sleep behavior disorder preceding other aspects of synuceinopathies by up to half a century. *Neurology;* 75:494–499.

Clare L, and Woods B. (2003). Cognitive rehabilitation and cognitive training for early‐stage Alzheimer's disease and vascular dementia. *The Cochrane Library*.

Connolly BS, and Lang AE. (2014). Pharmacological treatment of Parkinson disease: a review. *Journal of the American Medical Association*,311(16), 1670-1683.

Costa A, Peppe A, Serafini F, Zabberoni S, Barban F, Caltagirone C, and Carlesimo GA. (2014). Prospective memory performance of patients with Parkinson's disease depends on shifting aptitude: evidence from cognitive rehabilitation. *Journal of the International Neuropsychological*Society, *20(7),*717–726.

doi:10.1017/S1355617714000563

Cummings JL, Mega M, Gray K, Rosenberg-Thompson S, CarusiDA, and Gornbein J. (1994). The Neuropsychiatric Inventory comprehensive assessment of psychopathology in dementia. *Neurology,* 44(12),2308-2308.

Dalrymple-Alford, JC, MacAskill MR, Nakas CT, Livingston L, Graham C, Crucian GP, & Porter RJ. (2010). The MoCA well-suited screen for cognitive impairment in Parkinson disease. Neurology, 75(19), 1717-1725.

Delis DC, Kramer JH, KaplanE, and Ober BA. (2000). *California Verbal Learning Test – second edition. Adult version*. Manual. The Psychological Corporation. San Antonio: TX.

Delis D, KaplanE, and Kramer J. (2001). *Delis-Kaplan Executive Function System*. The Psychological Corporation, San Antonio, TX: Harcourt Brace and Company.

Del Tredici K, and Braak H. (2013). Dysfunction of the locus coeruleus-norepinephrine system and related circuitry in Parkinson's disease-related dementia. *Journal of Neurology, Neurosurgery, and Psychiatry,* *84(7),*774–783.

doi:10.1136/jnnp-2011-301817

De Medeiros K, Robert P, Gauthier S, Stella F, Politis A, Leoutsakos J, and Lyketsos C. (2010). The Neuropsychiatric Inventory-Clinician rating scale (NPI-C): reliability and validity of a revised assessment of neuropsychiatric symptoms in dementia. International Psychogeriatrics, 22(06), 984-994.

Dion M, Potvin O, Belleville S, Ferland G, Renaud M, Bherer L, . . . & Hudon, C. (2015). Normative data for the Rappel libre/Rappel indicé à 16 items (16-item Free and Cued Recall) in the elderly Quebec-French population. The Clinical Neuropsychologist, 28(Suppl.1), 1- 19. doi: 10.1080/13854046.2014.9 15058

Dirnberger G, and Jahanshahi M. (2013). Executive dysfunction in Parkinson's disease: a review. *Journal of Neuropsychology*, *7*(2), 193–224. doi:10.1111/jnp.12028

Doiron M, and Simard M. (2012). *Idiopathic Parkinson's disease, vascular risk factors and cognition: A critical review*.

Doiron M, Dupré N, Langlois M, Provencher P and Simard M. (In press). Smoking history is associated to cognitive impairment in Parkinson's disease, *Aging*  *et* Mental *Health*.

DOI: 10.1080/13607863.2015.1090393

Dorsey ER, Constantinescu R, Thompson JP, BiglanKM, Holloway RG, Kieburtz K, Marshall FJ, Ravina BM, Schifitto G, Siderowf A, and Tanner CM. (2007). Projected number of people with Parkinson disease in the most populous nations, 2005 through 2030. *Neurology*. 68, 384-6.

Doyon J, Gaudreau D, Laforce RJ, Castonguay M, Bedard PJ, Bedard F, and Bouchard JP. (1997). Role of the striatum, cerebellum, and frontal lobes in the learning of a visuomotor sequence. Brain and cognition, 34(2), 218-245.

Dujardin K, Tard C, Duhamel A, Delval A, Moreau C, Devos D, and Defebvre L. (2013). The pattern of attentional deficits in Parkinson's disease. *Parkinsonism and Related Disorders*, *19*(3), 300–305. doi:10.1016/j.parkreldis.2012.11.001

Duncan J. (1986). Disorganisation of behaviour after frontal lobe damage. *Cognitive Neuropsychology*. doi:10.1080/02643298608253360

Edwards JD, Hauser RA, O'Connor ML, Valdés EG, Zesiewicz TA, and Uc EY. (2013). Randomized trial of cognitive speed of processing training in Parkinson disease. *Neurology*, *81*(15), 1284–1290. doi:10.1212/WNL.0b013e3182a823ba

Fearnley JM, Lees AJ. (1991) . Ageing and Parkinson's disease: substantia nigra regional selectivity. *Brain.*114 (Pt 5), 2283-301.

Folstein MF, Folstein SE, and McHugh PR. (1975). "Mini-mental state": a practical method for grading the cognitive state of patients for the clinician. *J Psychiatric Res;* 12: 189-198.

Freedman M, and Stuss DT. (2011). Theory of Mind in Parkinson's disease. *Journal of the Neurological Sciences*, 310(1), 225-227.

Gallagher DA, O'Sullivan SS, Evans AH, LeesAJ, and Schrag A. (2007). Pathological gambling in Parkinson's disease: risk factors and differences from dopamine dysregulation. An analysis of published case series. *Movement Disorders,* 22(12), 1757-1763.

Gelb DJ, Oliver E, and Gilman S. (1999). Diagnostic criteria for Parkinson disease. *Archives of neurology*, 56(1), 33-39.

Gioia GA, Isquith PK, Guy SC and Kenworthy L. (2000). Test review behavior rating inventory of executive function. *Child Neuropsychology,* 6(3), 235-238.

Goetz CG, Poewe W, Rascol O, Sampaio C, Stebbins GT, Counsell C, Giladi N, Holloway RG, Moore CG, Wenning GK, Yahr MD, and Seidl L. (2004). Movement Disorder Society Task Force Report on the Hoehn and Yahr Staging Scale: Status and Recommendations: Movement Disorder Society Task Force on Rating Scales for Parkinson's Disease. *Movement Disorders*, 19(9), pp. 1020–1028.

Grandmaison É and Simard M. (2003). A critical review of memory stimulation programs in Alzheimer's disease. *The Journal of Neuropsychiatry and Clinical Neurosciences*, *15*(2), 130–144. doi:10.1176/jnp.15.2.130

Grober E, Buschke H, Crystal HA, Bang S, & Dresner R. (1988). Screening for dementia by memory testing. Neurology, 38(6), 900-903. doi: 10.1212/WNL.38.6.900

Guidi M, Paciaroni L, Paolini S, Scarpino O, and Burn DJ. (2015). Semantic profiles in mild cognitive impairment associated with Alzheimer's and Parkinson's diseases. *Functional Neurology*, 30(2), 113.

Hawkins K, Jennings D, Marek K, SiderowfA, and Stern M. (2010). Cognitive deficits associated with dopamine transporter loss in the pre-motor subjects in the PARS cohort. *Movement Disorders:*(25), S690-S691.

Hawkins NG, Sanson-Fisher RW, Shakeshaft A, D'Este C and Green LW. (2007). The multiple baseline design for evaluating population-based research. *American Journal of Preventive Medicine,* *33*(2), 162–168.

doi:10.1016/j.amepre.2007.03.020

Hérbert R, Bravo G, and Préville M. (2000). Reliability, validity, and reference values of the Zarit Burden Interview for assessing informal caregivers of community-dwelling older persons with dementia. *Canadian Journal on Aging*, 19, 494-507.

Hindle JV, Martyr A, and Clare L. (2014). Cognitive reserve in Parkinson's disease: a systematic review and meta-analysis. *Parkinsonism and related disorders*, 20(1), 1-7.

Hindle JV, Petrelli A, Clare L, and Kalbe E. (2013). Nonpharmacological enhancement of cognitive function in Parkinson's disease: a systematic review. *Movement Disorders*, *28*(8), 1034–1049. doi:10.1002/mds.25377

Hoehn MM, and Yahr MD. (1967). Parkinsonism: onset, progression and mortality. *Neurology;*17(5): 427-442.

Hooper HE. (1958). The Hooper Visual Organization Test: Manual. Los Angeles: Western Psychological Service.

Huckans, M., Hutson, L., Twamley, E., Jak, A., Kaye, J., and Storzbach, D. (2013). Efficacy of cognitive rehabilitation therapies for mild cognitive impairment (MCI) in older adults: working toward a theoretical model and evidence-based interventions. *Neuropsychology Review*, *23*(1), 63–80. doi:10.1007/s11065-013-9230-9

Hughes AJ, Daniel SE, Kilford L, and Lees AJ. (1992). Accuracy of clinical diagnosis of idiopathic Parkinson's disease: a clinico-pathological study of 100 cases. *Journal of Neurology, Neurosurgery, and Psychiatry,* *55(3),*181–184.

doi:10.1136/jnnp.55.3.181

Huntley JD, Gould RL, Liu K, Smith M, and Howard RJ. (2015). Do cognitive interventions improve general cognition in dementia? A meta-analysis and meta-regression. *BMJ Open*, *5*(4), e005247. doi:10.1136/bmjopen-2014-005247

Jellinger KA. (2006). The morphological basis of mental dysfunction in Parkinson's disease. *Journal of the Neurological Sciences,* *248*(1–2), 167–172.

doi:10.1016/j.jns.2006.05.002

Jellinger KA. (2012). Neuropathology of sporadic Parkinson's disease: evaluation and changes of concepts. *Movement Disorders,* *27(1),*8–30.

doi:10.1002/mds.23795

Jellinger KA, and Attems J. (2015). Challenges of multimorbidity of the aging brain: a critical update. *Journal of Neural Transmission,* *122(4),*505–521.

doi:10.1007/s00702-014-1288-x

Kalbe E, and Kessler J. (2015). Task force WANTED: many reasons to promote research on cognitive rehabilitation to prevent, delay, and treat cognitive dysfunctions in patients with Parkinson's disease. *Parkinsonism and Related Disorders,* *21*(2), 166–167.

doi:10.1016/j.parkreldis.2014.11.014

Kaplan E, GoodglassH, and Weintraub S. (2001). *Boston Naming Test*. Pro-edition.

Kawamura M, and Koyama S. (2007). Social cognitive impairment in Parkinson's disease. *Journal of Neurology*, 254(4), IV49-IV53.

Kehagia AA, Barker RA, and Robbins TW. (2010). Neuropsychological and clinical heterogeneity of cognitive impairment and dementia in patients with Parkinson's disease. *The Lancet Neurology,* *9*(12), 1200–1213.

doi:10.1016/S1474-4422(10)70212-X

Kesner RP and Creem-Regehr SH. (2013). Parietal contributions to spatial cognition.

Kessels R, van Zandvoort M, Postma A, Kappelle LJ, and haan EH. (2000). The Corsi Block-Tapping Task: Standardization and Normative Data, 252-258.

doi: 10.1207/S15324826AN0704_8

Kolb B, and Winshaw IQ. (2008). *Fundamentals of Human Neuropsychology (6th edition).* Worth Publisher. 818 pp.

Kudlicka A, Clare L, and Hindle JV. (2011). Executive functions in Parkinson's disease: Systematic review and meta-analysis. *Movement Disorders,*26(13), 2305-2315.

Kulisevsky J, Fernández de Bobadilla R, Pagonabarraga J, Martínez-Horta S, Campolongo A, García-Sánchez C, et al. (2013). Measuring functional impact of cognitive impairment: validation of the Parkinson's disease cognitive functional rating scale. *Parkinsonism and Related Disorders,* *19*(9), 812–817.

doi:10.1016/j.parkreldis.2013.05.007

Laforce R, and Doyon J. (2001). Distinct contribution of the striatum and cerebellum to motor learning. *Brain and cognition*, 45(2), 189-211.

Lavoie M, Callahan B, Belleville S, Simard M, Bier N, Gagnon L, Gagnon J-F, Blanchet S, Potvin O, Hudon C, Macoir J. (2013) Normative data for the Dementia Rating Scale in the French-Quebec Population. *The Clinical Neuropsychologist;* 27(7): 1150-1166.

Lawson RA, Yarnall AJ, Duncan GW, Breen DP, Khoo TK, Williams-Gray CH, ICICLE-PD study group. (2016). Cognitive decline and quality of life in incident Parkinson's disease: The role of attention. Parkinsonism and related disorders.

Lawson RA, Yarnall AJ, Duncan GW, Khoo TK, Breen DP, Barker RA, et al. (2014). Severity of mild cognitive impairment in early Parkinson's disease contributes to poorer quality of life. *Parkinsonism and Related Disorders,* *20*(10), 1071–1075.

doi:10.1016/j.parkreldis.2014.07.004

Lees AJ, Hardy J, and Revesz T. (2009). Parkinson's disease. *The Lancet*, *373*(9680), 2055–2066. doi:10.1016/S0140-6736(09)60492-X

Leentjens AF, Dujardin K, Marsh L, Martinez‐Martin P, Richard IH, Starkstein SE and Stebbins GT. (2008). Apathy and anhedonia rating scales in Parkinson's disease: critique and recommendations. Movement Disorders, 23(14), 2004-2014. Lesage, S., Brice, A., 2009. Parkinson's disease: from monogenic forms to genetic susceptibility factors. *Hum Mol Genet.* 18, R48-59.

Leung IH, Walton CC, Hallock H, Lewis SJ, Valenzuela M, and Lampit A. (2015). Cognitive training in Parkinson disease A systematic review and meta-analysis. *Neurology,*85(21), 1843-1851.

Levine B, and Downey-Lamb MM. (2005). *Design and evaluation of rehabilitation experiments. Neuropsychological interventions: clinical research and practice*. Eslinger PJ. New York: The Guilford Press.

Levine B, Robertson IH, Clare L, Carter G, Hong J, Wilson BA, et al. (2000). Rehabilitation of executive functioning: an experimental-clinical validation of goal management training. *Journal of the International Neuropsychological Society,* *6*(3), 299–312.

Levine B, Schweizer TA, O'Connor C, Turner G, Gillingham S, Stuss DT, et al. (2011). Rehabilitation of Executive Functioning in Patients with Frontal Lobe Brain Damage with Goal Management Training. *Frontiers Human Neuroscience*, *5*.

doi:10.3389/fnhum.2011.00009

Levine B, Stuss DT, Winocur G, Binns MA, Fahy L, Mandic M, et al. (2007). Cognitive rehabilitation in the elderly: effects on strategic behavior in relation to goal management. *Journal of the International Neuropsychological*Society, *13*(1), 143–152.

doi:10.1017/S1355617707070178

Liepelt-Scarfone I, Gräber S, Fruhmann Berger M, Feseker A, Baysal G, Csoti I, et al. (2012). Cognitive profiles in Parkinson's disease and their relation to dementia: a data-driven approach. *International Journal of Alzheimer's Disease*, *2012*, 910757.

doi:10.1155/2012/910757

Litvan I, Aarsland D, Adler CH, Goldman JG, Kulisevsky J, Mollenhauer B, et al. (2011). MDS Task Force on mild cognitive impairment in Parkinson's disease: critical review of PD-MCI. *Movement Disorders,* *26*(10), 1814–1824.

doi:10.1002/mds.23823

Litvan I, Goldman JG, Tröster AI, Schmand BA, Weintraub D, Petersen RC, et al. (2012). Diagnostic criteria for mild cognitive impairment in Parkinson's disease: Movement Disorder Society Task Force guidelines. *Movement Disorders,* *27(3),*349–356.

doi:10.1002/mds.24893

Matteau E, Dupré N, Langlois M, Provencher P, and Simard M. (2012). Clinical validity of the Mattis Dementia Rating Scale-2 in Parkinson disease with MCI and dementia. *Journal of Geriatric Psychiatry and Neurology,* *25(2),*100–106.

doi:10.1177/0891988712445086

Mattis S. (2001). Dementia Rating Scale-2. Lutz, Florida: Psychological Assessment Resources Inc.

McCaffrey RJ, Ortega A, and Haase RF. (1993). Effects of repeated neuropsychological assessments. *Archives of Clinical Neuropsychology*, 8(6), 519-524.

McGraw KO, and Wong SP. (1992). A Common Language Effect Size Statistic. *Psychological Bulletin*, 361–365.

Milman U, Atias H, Weiss A, Mirelman A, and Hausdorff JM. (2014). Can cognitive remediation improve mobility in patients with Parkinson's disease? Findings from a 12 week pilot study. *Journal of Parkinson's Disease,* *4*(1), 37–44.

doi:10.3233/JPD-130321

Mohlman J, Chazin D, and Georgescu B. (2011). Feasibility and acceptance of a nonpharmacological cognitive remediation intervention for patients with Parkinson disease. *Journal of Geriatric Psychiatry and Neurology,* *24(2),*91–97.

doi:10.1177/0891988711402350

Monchi O, Hanganu A, and Bellec P. (2016). Markers of cognitive decline in PD: the case for heterogeneity. *Parkinsonism and*  *Related Disorders*.

Müller MT, and Bohnen NI. (2013). Cholinergic dysfunction in Parkinson's disease. *Current Neurology and Neuroscience*Reports, *13(9),*377.

doi:10.1007/s11910-013-0377-9

Nasreddine ZS, Phillips NA, Bedirian V, Charbonneau S, Whitehead V, Collin I, et al. (2005). The Montreal Cognitive Assessment, MoCA: a brief screening tool for mild cognitive impairment. *J Am Geriatr Soc*, 53, 695-699.

Nombela C, Bustillo PJ, Castell PF, Sanchez L, Medina V, and Herrero MT. (2011). Cognitive rehabilitation in Parkinson's disease: evidence from neuroimaging. *Frontiers in Neurology*, *2*,82. doi:10.3389/fneur.2011.00082

Norman DA and Shallice T. (1986). Attention to action (pp. 1-18). Springer US.

Pagano G, Rengo G, Pasqualetti G, Femminella GD, Monzani F, Ferrara N, and Tagliati M. (2015). Cholinesterase inhibitors for Parkinson's disease: a systematic review and meta-analysis. *Journal of Neurology, Neurosurgery, and Psychiatry,* *86(7),*767–773.

doi:10.1136/jnnp-2014-308764

Pagonabarraga J, and Kulisevsky J. (2012). Cognitive impairment and dementia in Parkinson's disease. *Neurobiology of Disease,* *46(3),*590–596.

doi:10.1016/j.nbd.2012.03.029

Pagonabarraga J, Kulisevsky J, Llebaria G, García‐Sánchez C,Pascual‐Sedano B, and Gironell A. (2008). Parkinson's disease‐cognitiverating scale: A new cognitive scale specific for Parkinson's disease. *Movement Disorders*, 23(7), 998-1005.

Palavra NC, Naismith, SL, and Lewis, SJ. (2013). Mild cognitive impairment in Parkinson's disease: a review of current concepts. *Neurology Research International*.

Pan-Montojo F, and Reichmann H. (2014). Considerations on the role of environmental toxins in idiopathic Parkinson's disease pathophysiology. *Translational Neurodegeneration*, *3*(1), 10.

doi:10.1186/2047-9158-3-10

París AP, Saleta HG, de la Cruz Crespo Maraver M, Silvestre E, Freixa MG, Torrellas CP, et al. (2011). Blind randomized controlled study of the efficacy of cognitive training in Parkinson's disease. *Movement Disorders,* *26*(7), 1251–1258.

doi:10.1002/mds.23688

Parkinson J. (1817). An essay on the shaking palsy. Whittingham and Rowland, for Sherwood, Neely, and Jones. London.

Parnetti L, Calabresi P. (2006). Spatial cognition in Parkinson's disease and neuro-degenerative dementias. *Cogn Process;* 7: S77e8.

Peña J, Ibarretxe-Bilbao N, García-Gorostiaga I, Gomez-Beldarrain MA, Díez-Cirarda M, and Ojeda N. (2014). Improving functional disability and cognition in Parkinson disease: randomized controlled trial. *Neurology,* *83*(23), 2167–2174.

doi:10.1212/WNL.0000000000001043

Péron J, Vicente S, Leray E, Drapier S, Drapier D, Cohen R, and Vérin M. (2009). Are dopaminergic pathways involved in theory of mind? A study in Parkinson's disease. *Neuropsychologia,* *47(2),*406-414.

Petersen RC. (2004). Mild cognitive impairment as a diagnostic entity. *Journal of Internal Medicine*, 256(3), 183-194.

Petersen RC, and Morris JC. (2005). Mild cognitive impairment as a clinical entity and treatment target. *Archives of Neurology*, 62(7), 1160-1163.

Peto V, Jenkinson C, and Fitzpatrick R. (1998). PDQ-39: A review of the development, validation and application of a Parkinson's disease quality of life questionnaire and its associated measures. *Journal of Neurology,*245(Suppl. 1), S10–S14.

doi:10.1007/PL00007730

Petrelli A, Kaesberg S, Barbe MT, Timmermann L, Fink GR, Kessler J, and Kalbe E. (2014). Effects of cognitive training in Parkinson's disease: a randomized controlled trial. *Parkinsonism and Related Disorders,* *20*(11), 1196–1202.

doi:10.1016/j.parkreldis.2014.08.023

Petrelli A, Kaesberg S, Barbe MT, Timmermann L, Rosen JB, Fink GR, et al. (2015). Cognitive training in Parkinson's disease reduces cognitive decline in the long term. *European Journal of Neurology,* *22(4),*640–647.

doi:10.1111/ene.12621

Pirogovsky E, Schiehser DM, Obtera KM, Burke MM, Lessig SL, Song DD, et al. (2014). Instrumental activities of daily living are impaired in Parkinson's disease patients with mild cognitive impairment. *Neuropsychology,* *28(2),*229–237.

doi:10.1037/neu0000045

Poletti M, and Bonuccelli U. (2013). Acute and chronic cognitive effects of levodopa and dopamine agonists on patients with Parkinson's disease: a review. *Therapeutic Advances in Psychopharmacology*, *3*(2), 101–113.

doi:10.1177/2045125312470130

Poletti M, Emre M, and Bonuccelli U. (2011a). Mild cognitive impairment and cognitive reserve in Parkinson's disease. *Parkinsonism and Related Disorders,* *17*(8), 579–586.

doi:10.1016/j.parkreldis.2011.03.013

Poletti M, Enrici I, Bonuccelli U, Adenzato M. (2011b). Theory of Mind in Parkinson's disease. Behavioural Brain Research, 319, 342-350.

Politis, M., and Niccolini, F. (2015). Serotonin in Parkinson's disease. *Behavioural Brain*Research, *277,*136–145.

doi:10.1016/j.bbr.2014.07.037

Postuma RB, BergD, Stern M, Poewe W, Olanow CW, Oertel W, and Halliday G. (2015). MDS clinical diagnostic criteria for Parkinson's disease. *Movement Disorders,*30(12), 1591-1601.

Premack DG, and Woodruff G. (1978). Does the chimpanzee have a Theory of Mind? *Behavioural Brain Science;* 1: 515–26.

Reuter I, Mehnert S, Sammer G, Oechsner M, and Engelhardt M. (2012). Efficacy of a multimodal cognitive rehabilitation including psychomotor and endurance training in Parkinson's disease. *Journal of Aging Research*, *2012*, 235765.

doi:10.1155/2012/235765

Robben, S. H., Sleegers, M. J., Dautzenberg, P. L., van Bergen, F. S., ter Bruggen, J. P., & Rikkert, M. G. (2010). Pilot study of a three‐step diagnostic pathway for young and old patientswith Parkinson's disease dementia: screen, test and then diagnose. International Journal of Geriatric Psychiatry, 25(3), 258-265.

Robbins TW and Cools R. (2014). Cognitive deficits in Parkinson's disease: a cognitive neuroscience perspective. *Movement Disorders,*29(5), 597-607.

Robertson IH. (1996). *Goal Management Training:a clinical manual*. Cambridge, UK: PsyConsult.

Roca M, Torralva T, Gleichgerrcht E, Chade A, Arévalo GG, Gershanik O and Manes F. (2010). Impairments in social cognition in early medicated and unmedicated Parkinson disease. *Cognitive and Behavioral Neurology,* *23(3),*152-158.

Rolinski M, Fox C, Maidment I and McShane R. (2012). Cholinesterase inhibitors for dementia with Lewy bodies, Parkinson"s disease dementia and cognitive impairment in Parkinson"s disease. *The Cochrane Database of Systematic Reviews*, *3*, CD006504.

doi:10.1002/14651858.CD006504.pub2

Rosenthal E, Brennan L, Xie S, Hurtig H, Milber J, Weintraub D, et al. (2010). Association between cognition and function in patients with Parkinson disease with and without dementia. *Movement Disorders,* *25*(9), 1170–1176.

doi:10.1002/mds.23073

Roy M-A, Doiron M, Talon-Croteau J, Dupré N, and Simard M. (In preparation). Effects of Levodopa and other Dopaminergic Treatments on Cognition in Cognitively Intact and Mildly Impaired Parkinson's Disease Patients: A Systematic Review.

Sai Y, Zou Z, Peng K, and Dong Z. (2012). The Parkinson's disease-related genes act in mitochondrial homeostasis. *Neuroscience and Biobehavioral Reviews*, *36*(9), 2034–2043. doi:10.1016/j.neubiorev.2012.06.007

Sala SD, Baddeley A, Papagno C, and Spinnler H. (1995). Dual‐task paradigm: a means to examine the central executive. *Annals of the New York Academy of Sciences,*769(1), 161-172.

Salmon DP, Thal LJ, Butters N and Heindel WC. (1990). Longitudinal evaluation of dementia of the Alzheimer type A comparison of 3 standardized mental status examinations. *Neurology*, 40(8), 1225.

Sammer G, Reuter I, Hullmann K, Kaps M, and Vaitl D. (2006). Training of executive functions in Parkinson's disease. *Journal of the Neurological Sciences*, *248*(1-2), 115–119. doi:10.1016/j.jns.2006.05.028

Schapira AH, Jenner P. (2011). Etiology and pathogenesis of Parkinson's disease. *Movement Disorder.* 26, 1049-55.

Schmidt M. (1996). *Rey auditory verbal learning test: a handbook*. Los Angeles: Western Psychological Services.

Schrag A, Jahanshahi M, and Quinn N. (2000). What contributes to quality of life in patients with Parkinson's disease? *Journal of Neurology, Neurosurgery and Psychiatry,*69, 308–312.

Schweizer TA, Levine B, Rewilak D, O'Connor C, Turner G, Alexander MP, et al. (2008). Rehabilitation of executive functioning after focal damage to the cerebellum. *Neurorehabilitation and Neural Repair,* *22(1),*72–77.

doi:10.1177/1545968307305303

Sitzer DI, Twamley EW and Jeste DV. (2006). Cognitive training in Alzheimer's disease: a meta-analysis of the literature. *Acta Psychiatrica Scandinavica,* 114(2),75-90.

Shulman LM, Pretzer-Aboff I, Anderson KE, Stevenson R, Vaughan CG, Gruber-Baldini AL and Weiner WJ. (2006). Subjective report versus objective measurement of activities of daily living in Parkinson's disease. *Movement Disorders,*21(6), 794-799.

Shulman LM, Gruber-Baldini AL, Anderson KE, Vaughan CG, Reich SG, FishmanPS, and Weiner WJ. (2008). The evolution of disability in Parkinson disease. *Movement Disorders,*23(6), 790-796.

Smith EE, and Jonides J. (1999). Storage and executives processes in the frontal lobes. Science; 283 (5408): 1657-1661.

Sofi F, Cesari F, Abbate R, Gensini GF, and Casini A. (2008). Adherence to Mediterranean diet and health status: meta-analysis. *BMJ,* *337*(Sep11 2), a1344–a1344.

doi:10.1136/bmj.a1344

Sollinger AB, Goldstein FC, Lah JJ, Levey AI, and Factor SA. (2010). Mild cognitive impairment in Parkinson's disease: subtypes and motor characteristics. *Parkinsonism and Related Disorders,* *16*(3), 177–180.

doi:10.1016/j.parkreldis.2009.11.002

St-Hilaire A, Hudon C, Vallet G, Bherer L, Lussier M, Gagnon JF, Simard M, Gosselin N, Escudier F, Rouleau I and Macoir J. (In press). Normative Data for Phonemic and Semantic Verbal Fluency Test in the Adult French-Quebec Population and Validation Study in Alzheimer's Disease and Depression. *The Clinical Neuropsychologist:*30(1).

Stahl SM. (2008). *Stahl's essential psychopharmacology: neuroscientific basis and practical applications*. Third edition. Cambridge University Press, New York, 1117 pp.

Stern Y. (2002). What is cognitive reserve? Theory and research application of the reserve concept. *Journal of the International Neuropsychological Society,* 8(03), 448-460.

Stone VE, Baron-Cohen S and Knight RT. (1998). Frontal lobe contributions to theory of mind. *Journal of Cognitive*  *Neuroscience,* 10, 640-656.

Strauss E, Sherman EM and Spreen O. (2006). Report writing and feedback session. In *A compendium of Neuropsychological Tests: Administration, Norms, and Commentary*  (Third Edition). Oxford University Press; pp 86-97.

Stubberud J, Langenbahn D, Levine B, Stanghelle J, and Schanke AK. (2013). Goal management training of executive functions in patients with spina bifida: a randomized controlled trial. *Journal of the International Neuropsychological*Society, *19(6),*672–685.

doi:10.1017/S1355617713000209

Tanner CM. (1989). The role of environmental toxins in the etiology of Parkinson's disease. *Trends in Neurosciences,* *12*(2), 49–54.

doi:10.1016/0166-2236(89)90135-5

Thivierge S, Jean L, Vézina J and Simard M. (2014). A Randomized Cross-over Controlled Study on Cognitive Rehabilitation of Instrumental Activities of Daily Living in Alzheimer Disease. *Physical Comorbidity,* *22*(11), 1188–1199.

doi:10.1016/j.jagp.2013.03.008

Thivierge S, Simard M, Jean L, and Grandmaison É. (2008). Errorless learning and spaced retrieval techniques to relearn instrumental activities of daily livingin mild Alzheimer's disease: A case report study. *Neuropsychiatric Disease and Treatment,* *4*(5), 987–999.

Tröster AI. (2011). A précis of recent advances in the neuropsychology of mild cognitive impairment(s) in Parkinson's disease and a proposal of preliminary research criteria. *Journal of the International Neuropsychological*Society, *17(3),*393–406.

doi:10.1017/S1355617711000257

Tulving E. (1995). Organization of memory: Quo vadis. The Cognitive Neurosciences.

Van der Linden M, Coyette F, Poitrenaud J, Kalafat M, Calicis F, Wyns C, . . . Members of GREMEM. (2004). The 16-item free/recall test (RL/RI-16). In M. Van der Linden, S. Adam, A. Agniel, & Members of GRENEM (Eds.), The Evaluation of Memory Disorders: Presentation of Four Episodic Memory Tests with Their Calibration. (pp. 25-47). Marseille: Solal

van Hooren S, Valentijn S, and Bosma H. (2007). Effect of a structured course involving goal management training in older adults: A randomised controlled trial. *Patient Education*, *65*(2), 205–213. doi:10.1016/j.pec.2006.07.010

Van Zomeren, AH, and Brouwer WJ. (1994). Theories and concepts of attention. Clinical neuropsychology of attention (2), 7-38.

Vertstichel P and Cambier J. (2005). *Aphasias,*in Neuropsychology and Clinical Neurology of Behavior, 3rd edition. Presses de l'université de Montréal. 455-486

Weintraub D, Simuni T, Caspell-Garcia C, Coffey C, Lasch S, Siderowf A, et al. (2015). Cognitive performance and neuropsychiatric symptoms in early, untreated Parkinson's disease. *Movement Disorders,* *30*(7), 919–927.

doi:10.1002/mds.26170

Weintraub D and Burn DJ. (2011). Parkinson's disease: the quintessential neuropsychiatric disorder. *Movement Disorders*, 26(6), 1022-1031.

Wechsler D. (2008). Wechsler Adult Intelligence Scale-Fourth Edition. San Antonio, TX: Pearson.

Williams JR, Hirsch ES, Anderson K, Bush AL, Goldstein SR, Grill S, and Pontone, G. (2012). A comparison of nine scales to detect depression in Parkinson disease Which scale to use? *Neurology*, 78(13), 998-1006.

Williams-Gray CH, Foltynie T, Brayne CEG, Robbins TW, and Barker RA. (2007). Evolution of cognitive dysfunction in an incident Parkinson's disease cohort. *Brain,* *130* (7), 1787–1798.

doi:10.1093/brain/awm111

Wilson B, Cockburn J, Baddeley A, and Hiorns R. (1989). The development and validation of a test battery for detecting and monitoring everyday memory problems. *Journal of Clinical and Experimental Neuropsychology*, 11(6), 855-870.

Wilson BA, Alderman N, Burgess PW, Emslie H, Evans JJ. (1996). *The behavioural assessment of the dysexecutive syndrome*. Thames Valley Company; Bury St Edmunds.

Wirdefeldt K, Adami HO, Cole P, Trichopoulos D, Mandel J. (2011). Epidemiology and etiology of Parkinson's disease: a review of the evidence. *Eur J Epidemiol.* (26) Suppl 1, S1-58.

Woods B, Aguirre E, SpectorAE, and Orrell M. (2012). Cognitive stimulation to improve cognitive *functioning in people with dementia. Cochrane Database Syst Rev, 2.*

Zeighami, Y., Ulla, M., Iturria-Medina, Y., Dadar, M., Zhang, Y., Larcher, K.M. H., and Dagher, A. (2015). Network structure of brain atrophy in de novo Parkinson's disease.  *E-Life, 4, e08440.*

Zimmermann R, Gschwandtner U, Benz N, Hatz F, Schindler C, Taub E, and Fuhr P. (2014). Cognitive training in Parkinson disease: cognition-specific vs nonspecific computer training. *Neurology,* *82*(14), 1219–1226.

doi:10.1212/WNL.0000000000000287

Zimprich A, Biskup S, Leitner P, Lichtner P, Farrer M, Lincoln S, Kachergus J, Hulihan M, Uitti RJ, Calne DB, Stoessl AJ, Pfeiffer RF, Patenge N, Carbajal IC, Vieregge P, Asmus F, Muller-Myhsok B, Dickson DW, Meitinger T, Strom TM, Wszolek ZK, Gasser T. (2004). Mutations in LRRK2 cause autosomal-dominant parkinsonism with pleomorphic pathology. *Neuron*. 44, 601-7.

**ANNEX 1**

Table 1.1 - Diagnostic criteria for idiopathic PD according to the*United*  *Kingdom*  *Parkinson's Disease Society Brain Bank*(Hughes et al., 1992)

| 1. Diagnosis of Parkinson's syndrome |
| --- |
| Bradykinesia (slowness in voluntary movements with gradual reduction in the speed and range of repetitive movements).  At least one other symptom among:   - Muscle rigidity, - Tremors at rest of the order of 4-6 Hz, - Postural instability that cannot be explained by visual, vestibular, cerebellar or proprioceptive dysfunction. |
| 2. Exclusion criteria for idiopathic PD |
| - History of repeated infarction with atypical progression of parkinsonian symptoms, - History of repeated head trauma, - History of confirmed encephalitis, - Oculogyre crises, - Treatments with neuroleptics during the onset of symptoms, - More than one loved one with symptoms, - Spontaneous remission, - Symptoms only unilateral after 3 years, - Supra-nuclear paralysis, - Focal neurological signs, - Severe dysfunction of the autonomic nervous system at the beginning of the disease, - Severe dementia with impaired memory, language or praxies at the beginning of the disease, - Presence of Babinski's reflex, - Presence of a brain tumor or hydrocephalus marked according to neuroimaging results, - Negative response to L-DOPA (excluding malabsorption), - Exposure to MPTP. |
| 3. Other criteria supporting the diagnosis (at least 3 symptoms to make a well-defined diagnosis of idiopathic PD) |
| - Unilateral beginning, - Presence of tremors at rest, - Progressive development, - Persistent asymmetry affecting the same side as at the beginning of symptoms, - Excellent response to L-DOPA (70% to 100%), - Response to the L-DOPA for 5 years or more, - Clinical development spanning 10 years or more. |

Table 1.2 - Diagnostic criteria for idiopathic PD according to the*National Institute of Neurological Disorders and Stroke* (Gelb et al., 1999)

| Group A: Characteristics of PD |
| --- |
| - Tremors at rest, - Bradykinesia, - Rigidity - Onset of asymmetric symptoms. |
| Group B: Characteristics suggesting other possible diagnoses |
| When early in clinical symptoms:   - Dominant postural instability in the first three years, - Phenomenon of*"freezing"*during the first three years, - Hallucinations not related to medication, - Development of dementia before motor symptoms or during the first year, - Supra-nuclear paralysis, or slowing down of visual jerks, - Severe dysfunction of the autonomic nervous system at the beginning of the disease, - Another medical cause that may better explain the symptoms of parkinsonism, such as a brain injury or brain tumor. |
| Criteria for possible diagnosis of idiopathic PD |
| - At least two of the symptoms mentioned in A, at least one of which is bradykinesia or tremors, - None of the characteristics of group B are present at least 3 years after the onset of symptoms, - Good response to L-DOPA or a dopaminergic agonist. |

Table 1.3 - Diagnostic criteria for idiopathic PD by *The Movement Disorder Society*(Postuma et al., 2015)

| Features supporting MP diagnosis |
| --- |
| Mandatory criteria:Parkinsonism union   - Bradykinesia, - Rigidity - Tremors at rest.   Other features that support diagnosis   - Frank and clear response to dopaminergic therapy, - Presence of Dyskinesia induced by L-DOPA, - Tremor at rest of a limb according to a clinical evaluation, - Presence of anosmia (loss of olfaction) or cardiac denervation according to a scintigraphy. |
| Features excluding the diagnosis of PD |
| Characteristics automatically excluding the diagnosis of MP   - Severe dysfunction of the autonomic nervous system at the beginning of the disease, - Supra-nuclear paralysis, or slowing down of visual jerks, - Probable diagnosis of frontotemporal dementia or progressive primary aphasia in the first five years, - Syndrome of parkinsonism restricted to the lower limbs for at least the first three years, - Anti-dopaminergic medication that is consistent with the appearance of parkinsonism symptoms, - Lack of response to high doses of L-DOPA, even when motor symptoms are moderate or severe, - Loss of cortical sensation (progressive aphasia, agraphia, loss of use of a single limb, etc.), - Normal functioning of the presynaptic dopaminergic system, according to functional imaging, - Another medical cause that may better explain the symptoms of parkinsonism, such as a brain injury or brain tumor.   Features that may suggest a diagnosis other than that of MP   - Rapid progression of postural instability requiring the use of a wheelchair less than five years following diagnosis, - Complete absence of progression ofmotor symptoms for at least five years unless relatedto pharmacological treatment, - Severe nerve dysfunction affecting language production: severe dysarthria, dysphonia or dysphagia, less than five years after diagnosis, - Breathing dysfunction (especially inspiration), whether day or night, - Severe failure of the autonomic nervous system lessthan five years following diagnosis,for example:   - Orthostatic hypotension,   - Severe urinary retention, or severe urinary incontinence less than three years following diagnosis, - Recurrent falls due to serious balance problems less than three years after diagnosis, - Disproportionate contractions of the feet or hands less than 10 years followingdiagnosis, - Absence of non-motor symptoms frequently associated with PD less than five years after diagnosis, such as:   - Sleep problems (REM sleep disorder, insomnia, etc.),   - Mild dysfunction of the autonomic nervous system (constipation, daytime urinary emergencies, etc.)   - Anosmia   - Psychological and behavioral symptoms (depression, apathy, anxiety, etc.) - Other unexplained pyramidal symptoms, - Bilateral Parkinsonism, reported by the patient, a relative or the clinician. |
| Types of PD diagnostics |
| Clinically established PD:   - Absence of a characteristic automatically excluding the diagnosis of PD, - At least two criteria supporting the diagnosis of PD, - Lack of characteristic that may suggest a diagnosis other than that of PD.   Clinically probable PD:   - Absence of a characteristic automatically excluding the diagnosis of PD, - At least two criteria supporting the diagnosis of PD, - Presence of no more than two characteristics that may suggest a diagnosis other than that of PD, but there must be at most as many of these characteristics as there are characteristics supporting the diagnosis. So, if there are two characteristics that may suggest another diagnosis, there must be the presence of two characteristics supporting the diagnosis. |

Table 1.4 – Summary of Neuropsychological Tests Sensitive enough to assess MCI in PD

| Cognitive domain | Recommended neuropsychological tests |
| --- | --- |
| Attention and working memory | - Empan place, reverse and ascending order of wais-IV (Weschler, 2008). - Letter-number sequences of the WAIS-IV (Weschler, 2008). - D-KEFS Trail Making Test (TMT), conditions 1-3 (Delis, Kaplan and Kramer, 2001). - Stroop test, version of the D-KEFS (Delis, Kaplan and Kramer, 2001). |
| Executive functions | - Test of verbal fluency, particularly the condition of alternation, version of the D-KEFS (Delis, Kaplan and Kramer, 2001). - "Behavioral*Assesment of Dysexecutive Syndrome"*(BADS), a battery of several ecological tests (Wilson et al., 1996). |
| Language | - Denomination test under confrontation, such as the Boston Naming Test (Kaplan, Goodglass, & Weintraub, 1983). |
| Episodic memory | - List of words to learn with immediate and delayed recall, such as the*California Verbal Learning Test*(CVLT) (Delis, Kramer, Kaplan and Ober, 2000) or theRey*Auditory Verbal Learning Test*(RAVLT) (Schmidt, 1996). - Visual Episodic Memory:*"Brief Visuospatial Memory Test – Revised"*(Benedict et al., 1996). |
| Visuospatial functions | - Benton Line Orientation Judgment Test (Benton, 1992). - Hooper's visual organization test(Hooper, 1958). |
| Global cognition | - MoCA(Nasreddine et al., 2005)or MMSE (Folstein et al., 1975). - Mattis Dementia^Scale-2nd^ edition (Mattis, 2001). |

**ANNEX 2**

The clinical stages of PD of Hoehn and Yahr (1967) revised (Goetz et al., 2004)

| Stadium | Description |
| --- | --- |
| 1 | Unilateral motor symptoms only, often in the upper limbs |
| 1.5 | Unilateral and axial movements, the trunk is also taken by involuntary tremors |
| 2 | Bilateral problems of the movement, but without problems of balance |
| 2.5 | Bilateral problems of movement with some balance difficulties, but recovery in the push test |
| 3 | Mild or moderate bilateral difficulties and some postural instability, but physical independence preserved |
| 4 | More severe difficulties, but ability to stand and move with a walker or cane |
| 5 | Wheelchair travel and needed assistance with basic care and daily activities |

**ANNEX 3**

Petersen's (2004) decision tree to characterize TCL

**ANNEX 4**

**Battery of tests for diagnostic assessment**

*Attention and working memory.*

Baddeley's dual task (Baddeley et al., 1997) measures an individual's performance under two types of conditions: when performing a task alone, or when two tasks need to be performed at the same time. In this case, the participant must first perform a verbal empan task (e.g. repeat sequences of numbers), then a visual pursuit task (e.g. draw Xs in squares printed on a sheet in a certain order), and then he must do both tasks at the same time. The score for this task is the difference between the performance in the single condition and the performance in the double condition. The total score (U) is calculated as follows:

**U = ( 1 – (Pm + Pt/2) ) * 100**

*Where* ***Pm***  *is the difference between single and double condition performance for the memorization task; and*  ***Pt*** is the difference between single and double condition performance for the visual *pursuit task. So the higher the percentage score, the better the performance.*

The Trail Making Test or TMT (conditions 1 to 3) of the D-KEFS (Delis, Kaplan and Kramer, 2001) will also be used. For all three conditions, the sheets have many scattered numbers and letters. Condition 1 corresponds to a simple visual scan task (e.g. cross out the 3). Condition 2 is to link a sequence of numbers by a line (1, 2, 3, 4, etc.). Condition 3 consists of linking a sequence of letters by a line (A, B, C, D, etc.). The time required to complete each condition is the score on this test.

The Corsi Blocks (Kessels et al., 2000) measure the spatial empan. On a structure comprising cubes scattered on the surface, the experimenter points to a sequence of cubes. The participant must then correctly point to the sequence he has just seen, in the same order, and then in reverse order. The maximum number of correctly pointed cubes is the spatial empan score.

The WAIS-IV empan task (Weschler, 2008) measures the verbal empan using a sequence of numbers that the participant must repeat in reverse order after hearing it from the examiner. In this case, it is the maximum number of digits stated correctly in reverse order is the score of this test.

*Language functions.*

A verbal fluency test will be used. First, the first condition evaluated will be a recently standardized version for the Quebec population of lexical fluidity (e.g. naming as many words as possible starting with the letters T, N and P, one minute per letter) (St-Hilaire et al., 2016). Then, the second condition will be a condition of semantic fluidity. The standardized task for the Quebec population will be used (e.g. name as many animals as possible in one minute) (St-Hilaire et al., 2016).

The Subtest "Denomination of the Neuropsychological Assessment Battery" is used to measure the denomination(White and Stern, 2003). The experimenter shows images of a familiar object. The subject must correctly name the objects shown in the image. One point per correct answer is awarded when the participant correctly names the object.

*Episodic memory.*

In the"B*rief*  *Visuospatial*  *Memory* Test – *Revised"*(BVMT–R; Benton, 1992), 15 complex shapes are presented to the participant and the participant must then choose the drawing he has seen among other choices. The form of recognition was chosen because of the motor disabilities associated with PD. For the rating, the number of correct answers chosen is the variable of interest.

The Free Recall – 16-Item Indicative Recall test (RL/RI-16, Van der Linden, Coyette, Poitrenaud, Kalafat, Calicis, Wyns, et al., 2004) was chosen to assess episodic memory in verbal modality. This test is a French translation of the "Free*and Cued Seclective Reminding Test"*(Buschke, 1984; Grober, Buschke, Crystal, Bang, & Dresner, 1988). In particular, it makes it possible to differentiate the difficulties of encoding, recognition and semantic memory. First, a list of 16 words is taught to the participant. These words are written on boxes in groups of four. In order for the participant to identify each of the words, a semantic clue is provided. An immediate indicative reminder is made for each of the four boards. The participant has three tries per board to learn the words. Then, an interference task (i.e. count backwards for 20 seconds) is requested from the participant. Afterwards, the participant must immediately recall as many words as possible in two minutes, regardless of the order. For each word that has not been recalled, the semantic index is provided again to help the participant recall the word. In total, three trials are administered to recall as many words as possible. After that, a delay of 20 minutes is left, and then another free and indexed delayed recall attempt is made. Then, a recognition task isadministered, in which the initial 16 words, 16 distractors belonging to the same semantic categories as well as 16 other distractors that have no connection with the initial words are all shown. For the quotation, each word correctly recalled is worth a point, whether in free recall, indexed, or recognition. For the latter, it is also important to note the distractors named by the participant. Quebec standards are available for this test (Dion, Potvin, Belleville, Ferland, Renaud & al., 2015).

*Visuospatial functions.*

The Line Orientation Judgment test (Benton, 1994) is a test in which two lines with different angles are presented to the participant. Then 11 reference lines scattered in a semicircle on a sheet are shown. The participant must correctly couple each of the two s-lines to one of the 11 reference lines. In all, the test includes 30 items. The total score is the number of correct answers chosen by the participant.

The WAIS-IV Visual Puzzle subtest (Weschler, 2008) is a test in which a series of complex figures are shown to the participant. For each figure, the participant must choose three simple shapes from six that adequately complement the complex figure. To have a point, the participant must correctly choose the three shapes for each item (1 or 0 per item, for a possible maximum of 24).

*Executive functions.*

First, a subtest of the BADS was chosen (Wilson et al., 1996). For themodified task of the Six elements, the participant must complete six activities and must advance as much as possible in 10 minutes. The goal is therefore to observe its organizational and planning capacities. The score for this task is calculated based on the number of completed tasks, minus the number of tasks that contain an error. Thetime required and the number of errors correspond to a profile of points, between zero and four (four corresponding to a perfect score).

The last two conditions (4 and 5) of the TMT of the D-KEFS (Delis, Kaplan and Kramer, 2001) make it possible to assess alternating capacities and psychomotor speed respectively. Again, it is the time required to complete the task that is the variable of interest. Condition 4 measures the ability to alternance because the subject must connect in the letters in alphabetical order and the numbers in ascending order and alternating them (e.g. A, 1, B, 2, C, 3, etc.). The last condition makes it possible to control for motor deficits, because it is a question of drawing a line on a dotted line as quickly as possible. It will be possible to correct the times obtained during conditions 1 to 4 with the score of condition 5. The corrected condition 4 score (in seconds) will be the variable here:

***Corrected Score Condition 4 = Score Condition 4 – Score Condition 5***

*Theory of mind.* The Faux Pas task was chosen (Stone et al., 1998), since it has already been used in the literature in PD patients to show that there was a deficit in theory of mind (cognitive component) in these patients(Kawamura and Koyama, 2007; Péron et al., 2009; Roca et al., 2010). The procedure is to read 20 short stories to the participant and check if he correctly identifies the misstep that was committed for 10 of the stories. Eight questions per story are then asked to make sure they understand. The questions concern the detection of the misstep, the understanding of the misstep, the understanding of the intentions of the protagonists, the understanding of beliefs, the understanding of emotions as well as the general understanding of the story. For the quotation, one point per correct answer is given to the participant (8 questions times 20 stories: maximum possible total of 160 points). However, if the control questions are not successful, it is not possible to give the points to the participant. In addition, different ratios can be calculated and appear more relevant to performance analysis according to the test authors (Stone et al., 1998). These ratios are calculated as a percentage and are explained in detail in the test manual:

1 – Successful number of control questions, for stories with and without missteps.

2 – Correct missteps detection score.

3 – Score of understanding of missteps.

4 – Score of understanding of intentions.

5 – Belief understanding score.

6 – Emotion comprehension score.

*Global cognition*. The "Montreal*Cognitive Assesment"*(MoCA, Nasreddine et al., 2005) is a screening measure that includes 11 very brief sub-tests measuring visuospatial functions, executive functions, denomination, memory, attention, language, abstraction and time orientation. The correct answers, added together, make it possible to calculate a maximum possible and positive score of 30.

**ANNEX 5:**

Fatigue scale filled at the end of each session with participants

**ANNEX 6**

Estimated time required for each of the GMT diagnostic assessment and efficacy measures

Table 6.1 - Diagnostic Assessment

| Built measured | Name of tests / questionnaires | Time required (minutes) |
| --- | --- | --- |
| Attention and working memory (2) | 1 – Baddeley's double task  2 – TMT conditions 1 - 3  3 – Corsi Blocks  4 – Empan upside down from WAIS-IV | 15-20  5-10  5  5 |
| Language functions (2) | 1 – D-KEFS lexical (NPT) and semantic (animal) verbal fluence test  2 – Boston Naming Test | 5  15 |
| Memory (2) | 1 – Brief Visuospatial Memory Test – Revised (BVMT–R)  2 – Free Reminder – Reminder Indicative of 16 items | 10-15  20-25 |
| Visuospatial functions (2) | 1 – Judgment of orientation of the lines (Benton)  2 – WaiS-IV visual puzzle | 15  15 |
| Executive functions (3) | 1 – Trail Making Test, conditions 4 – 5  2 – Task of the six elements of the BADS | 5-10  10-15 |
| Global cognition | 1 – MoCA | 15 |
| Theory of mind | 1 – Misstep Task | 30-40 |

Maximum total time: 3 hours, spread over 3 sessions of 60 to 75 minutes each maximum.

Table 6.2 - Effectiveness of the GMT: Primary Measures

| Built measured | Measurements | Time required (minutes) |
| --- | --- | --- |
| Tolerability / security | 1 – Attendance at the proposed sessions and activities; changes in medication  2 – Evaluation of the participant's diary (if mention of fatigue) and evaluation of the fatigue felt by the patient on  a Likert scale before and after each session | 5  5 |
| Executive functions | 1 – Dysexcutive (DEX) questionnaire  2 – GMT questionnaires (participating version) included in the package  3 – BADS Zoo Map Test | 10-15  10  10-15 |

Maximum total time: 50 minutes.

Table 6.3 - Effectiveness of the GMT: Secondary Measures

| Built measured | Name of tests / questionnaires | Time required (minutes) |
| --- | --- | --- |
| Cognition | Dementia Rating Scale – II | 15-45 |
| AIVQ / Quality of life | PDQ-39 | 20-25 |
| Executive functions of the participant (PROCHE) | 1 – Dysexcutive (DEX) questionnaire, helping version  2 – GMT questionnaires, helping version | 10-15  10 |
| Caregiver Burden (PROCHE) | Zarit Bruden Interview - 12 | 10-15 |
| Participant's Cognitive and Behavioral Symptoms (NEAR) | Neuropsychiatric Inventory, helper and clinician versions | 15 |

Maximum total time: for the participant, 1h; for relatives, 55 minutes.

**ANNEX 7**

Table 7.1 - Test Planning for the Pilot Study: Participant 1 (two baseline measures)

Table 7.2 - Test Planning for the Pilot Study: Participant 2 (three baseline measures)

Table 7.3 - Test planning for the randomized study – control

**ANNEX 8**

**Adaptations of the GMT for MP-TCL patients,co-matching with the original GMT**

**ANNEX 9**

**Provisional plan for psychoeducation** sessions in the active control group

*** Each psychoeducation session lasts approximately 30 to 40 minutes, followed by a 20 to 30 minute mindfulness exercise session (breathing and body scanning exercises). Participants will not have to do exercises between sessions themselves, unless there is a personal initiative.*

Session 1: General presentation of the PD

- Dopaminergic system (alterations in)
- Neuroanatomy (affectedstructures)
- Motor symptoms, subtypes and symptom progression
- Types of treatments available tips and tricks to manage these symptoms (discussion with the loved one)

Session 2: Non-motor symptoms

- Sleep
  - REM sleep disorder
  - Insomnia / hypersomnia
- Disorders of the autonomic nervous system
  - Smell
  - Digestion
- Available treatments, tips and tricks to manage these symptoms (discussion with the patient and the relative)

Session 3: Non-motor symptoms (psychological and behavioral symptoms)

- Depression and anxiety
- Psychotic symptoms
- Available treatments, tips and tricks to manage these symptoms (discussion with the patient and the relative)

Session 4: Non-motor symptoms (cognition 1 – cognitive functions)

- Memory
- Careful
- Language
- Visuospatial functions
- Executive functions

Session 5: Non-motor symptoms (cognition 2 – cognition and MP)

- Profile of impairments in PD
- MP-TCL
- Parkinson's dementia
- Available treatments, tips and tricks to manage these symptoms (discussion with the loved one)
