# Supplementary material for: Goal management training and psychoeducation / mindfulness for treatment of executive dysfunction in Parkinson’s disease: A feasibility pilot trial
Source: PLoS One. 2022 Feb 18;17(2):e0263108. doi: 10.1371/journal.pone.0263108 (PMC8856541; doi:10.1371/journal.pone.0263108)
Supplement: S2 File — (ZIP) [file pone.0263108.s003.zip › Protocole CE╠üR 2018-05-30.pdf]

Ariane GIGUÈRE-RANCOURT

«*Goal Management Training*»: Étude sur l'efficacité d'un programme d'entraînement des fonctions exécutives chez des patients atteints de la maladie de Parkinson idiopathique

Projet de thèse présenté à

L'École de psychologie de l'Université Laval

Pour l'obtention du grade de

Doctorat en psychologie, recherche et intervention (neuropsychologie)

Philosophiæ doctor (Ph.D.)

Faculté des Sciences Sociales

Université Laval

30 mai 2018

Québec, Canada

© Ariane Giguère-Rancourt, 2018

Liste des abréviations

ACh Acétylcholine

AChE Acétylcholinestérase

ACT *Adaptative Control of Thought*

AIVQ Activités instrumentales de la vie quotidienne

AVQ Activités de la vie quotidienne

BADS *Behavioral Assessment of Dysexecutive Syndrome*

BChE Butyrilcholinestérase

BDNF *Brain Derived Neurotrophic Factor*

CLES *Common Language Effect Size*

COMT Cathécol-O-Méthylransférase

CVLT *California Verbal Learning Test*

DA Dopamine

D-KEFS *Delis and Kaplan Executive Function System*

DRS-2 *Dementia Rating Scale – 2*

FDA *Food and Drug Administration*

GMT *Goal Management Training*

IChE Inhibiteur de Cholinestérases

IMAO-B Inhibiteur de la Monoamine Oxydase B

IRMf Imagerie par Résonnance Magnétique Fonctionnelle

L-DOPA Lévodopa

LRRK2 *Leucine-Rich Repeat Kinase 2*

MA Maladie d'Alzheimer

MMSE *Mini Mental State Examination*

MoCA *Montréal Cognitive Assessment*

MP Maladie de Parkinson

MP-TCL Patients atteints de la maladie de Parkinson avec trouble cognitif léger

MPTP 1-méthyle-4-phényl-1,2,3,6-tétrahydropyridine

NINDS *National Institute of Neurological Disorders and Stroke*

NMDA N-Méthyl-D-Aspartate

NPI-12 *NeuroPsychiatric Inventory – 12 items*

PDQ-39 *Parkinson Disease Questionnaire – 39 items*

PINK-1 *PTEN-Induced Putative Kinase 1*

RL/RI-16 Rappel Libre – Rappel Indicé à 16 items

RAVLT *Rey Auditory Verbal Learning Test*

SAS *Supervisory Attentionnal System*

SPC Symptômes Psychologiques et Comportementaux

SPI Sériel Parallèle Indépendant

TCL Trouble Cognitif Léger

TDA Trouble Déficitaire de l'Attention

TMT *Trail Making Test*

UKPDBBS *United Kingdom Parkinson's Disease Brain Bank Society*

WAIS-IV *Wechsler Adult Intelligence Scale - IV*

ZBI-12 *Zarit Burden Interview – 12 items*

Problématique

La maladie de Parkinson (MP) est une atteinte neurodégénérative caractérisée principalement, au niveau moteur, par des tremblements au repos et une rigidité musculaire. Après la maladie d'Alzheimer (MA), il s'agit de la deuxième maladie neurodégénérative la plus prévalente chez la population des 65 ans et plus (Jellinger et Attems, 2015; Lees, Hardy, et Revesz, 2009; Williams-Gray, Foltynie, Brayne, Robbins, et Barker, 2007). Dans la population générale, le risque de développer la MP est estimé à 14 cas pour 100 000 personnes, mais chez les 65 ans et plus, ce risque augmente à 160 cas sur 100 000 personnes (Wirdefeldt, Adami, Cole, Trichopoulos et Mandel, 2011). Globalement, le risque de développer la MP au cours d'une vie est estimé à environ 1,3% chez les femmes et à 2% chez les hommes (Wirdefelt et al., 2011).

Outre les symptômes moteurs de la MP, de nombreux autres symptômes sont observés (Aarsland et al., 2009; Kalbe et Kessler, 2015; Weintraub et al., 2015), tels qu'un état dépressif, apathique, anxieux ainsi que des problèmes cognitifs, qui vont du trouble cognitif léger (TCL) jusqu'au trouble cognitif majeur, aussi appelé démence parkinsonienne. La proportion de patients atteints de la MP qui développeront un TCL varie entre 19 et 36% selon les études (Aarsland et al., 2009; Aarsland, Brønnick, et Fladby, 2011). Malheureusement, il n'existe actuellement aucun traitement curatif pour les patients MP: il n'y a que des traitements symptomatiques, qui retardent tout au plus la progression des symptômes moteurs (Lees et al., 2009). Les recherches actuelles se penchent donc sur des traitements curatifs et sur l'identification de gènes ou de facteurs de risque associés au développement de la MP (Jellinger et Attems, 2015). Malgré ces efforts constants qui sont faits en vue de modifier le cours de la maladie par des méthodes pharmacologiques, il existe aujourd'hui très peu d'alternatives pour les patients souffrant de MP présentant des troubles cognitifs (Jellinger, 2006; Pagonabarraga et Kulisevsky, 2012; Sollinger, Goldstein, Lah, Levey, et Factor, 2010). À ce jour, un seul médicament nootrope, la rivastigmine, a été approuvé par Santé Canada pour le traitement des troubles cognitifs dans la MP, et seulement pour les cas de démence. Étant donné que le développement de médicaments peut prendre plusieurs dizaines d'années, d'autres avenues thérapeutiques, non-pharmacologiques, pourraient s'avérer utiles afin d'aider les patients qui sont présentement aux prises avec un TCL. Le présent projet propose donc de tester

l'efficacité d'un programme d'entraînement cognitif qui serait adapté aux patients atteints de la MP et qui pourrait améliorer ou contribuer à maintenir leur autonomie au quotidien. Pour permettre une mise en contexte du projet, les prochaines sections présenteront d'abord les principales caractéristiques neuropathologiques, neurochimiques et cliniques de la MP et du TCL dans la MP. Ensuite, les études ayant déjà porté sur des interventions cognitives dans la MP seront présentées et critiquées. Globalement, des résultats prometteurs ressortent de ces études, mais il existe une grande variabilité dans les méthodologies employées. Il est donc difficile de tirer des conclusions claires de ces données. Également, peu de programmes sont réellement transférables dans le quotidien des patients. Afin de répondre à certaines de ces limites, le programme d'entraînement choisi, le «*Goal Management Training*» (GMT) sera abordé. Les objectifs des deux phases du projet, les hypothèses et les résultats attendus seront exposés. Ensuite, la méthodologie qui sera employée pour parvenir à réaliser les objectifs visés sera expliquée et les considérations éthiques seront aussi présentées. Enfin, il y aura une brève conclusion sur les retombées potentielles de ce projet.

Introduction

Présentation de la maladie de Parkinson (MP)

La MP a été décrite pour la première fois en 1817 par le neurologue James Parkinson comme une «paralysie agitante» (Parkinson, 1817). Par la suite, d'autres symptômes ont été décrits au cours du 19e siècle, tels que la rigidité, la rareté des mouvements, la micrographie et l'expression faciale figée, symptômes qui font maintenant partie des critères diagnostiques de la maladie (Hughes, Daniel, Kilford, et Lees, 1992). La MP est ainsi une maladie surtout connue pour ses symptômes moteurs, causée par d'importantes altérations du cerveau. Toutefois, plusieurs autres manifestations non-motrices sont aussi fréquemment retrouvées, elles aussi induites par plusieurs lésions et dysfonctions cérébrales (Dirnberger et Jahanshahi, 2013; Pagonabarraga et Kulisevsky, 2012).

<u>Bases neuropathologiques et neurochimiques</u>

Synucléinopathie. Il est généralement accepté que la MP est une protéinopathie, c'est-à-dire une maladie qui implique des agrégats de protéines anormales dans le cerveau (Jellinger, 2012; Lees et al., 2009). Plus précisément, la MP est une synucléinopathie : elle implique donc des agrégats anormaux

d'alpha-synucléine (α-synucléine), une protéine dont le rôle est encore mal compris. Il est quand même connu que l'α-synucléine serait impliquée dans le recyclage des neurotransmetteurs et dans la production de radicaux libres (Jellinger, 2012). Les agrégats d'α-synucléine forment des corps de Lewy, de petites inclusions cytoplasmiques d'environ 15 µm de diamètre, utilisés aujourd'hui comme marqueurs pathologiques pour le diagnostic post-mortem de la MP (Jellinger, 2012). Les corps de Lewy se lieraient aux mitochondries et en diminueraient l'activité, ce qui provoquerait éventuellement un stress oxydatif et une mort neuronale. En fait, les corps de Lewy appraîtraient bien avant le début des symptômes moteurs et se propageraient depuis le tronc cérébral vers les structures sous-corticales (substance noire, noyau dorsal du nerf vague, noyau basal de Meynert), pour atteindre le système limbique et le cortex frontal lors des stades plus avancés de la maladie (Jellinger, 2012; Lees et al., 2009). De plus, des corps de Lewy sont retrouvés au niveau de la moëlle épinière et de plusieurs nerfs du système nerveux périphérique autonome.

Afin de caractériser l'évolution de la maladie, Braak et ses collègues (Braak, Del Tredici, Rüb, de Vos, Jansen Steur et Braak, 2003) ont établi différents stades de propagation des corps de Lewy et ont pu les corréler avec les atteintes motrices et cognitives. Au stade 1, une atteinte de la medulla oblongata (noyau moteur et zone réticulaire interne) est observée. Au stade 2, les lésions et anomalies atteignent le noyau du raphé, le noyau réticulaire gigantocellulaire ainsi que le locus coeruleus et subcoeruleus. Toujours selon ce modèle, les stades 1 et 2 sont cliniquement asymptomatiques. Lors du stade 3, des atteintes de la substance noire *pars compacta* s'ajoutent aux lésions précédentes. Au stade 4, les lésions atteignent le mésocortex transentorhinal et l'allocortex. Au stade 5, les aires associatives sensorielles du néocortex et le néocortex préfrontal sont touchés. Finalement, au stade 6, ce sont les aires primaires et associatives du néocortex et les aires prémotrices qui sont atteintes. De manière générale, ces stades corrèlent avec la gravité des symptômes cliniques de la MP et les stades 5 et 6 sont corrélés avec le développement d'une démence (Braak et al., 2003).

En plus des corps de Lewy, les patients atteints de MP développent également des plaques séniles formées par l'agrégation du peptide amyloïde-bêta$_{40\text{-}42}$ (Jellinger, 2012) ainsi que des enchevêtrements neurofibrillaires, formés par l'hyperphosphorylation de la protéine tau (Jellinger, 2012). Toutefois, il

semblerait que ces dernières altérations soient plutôt une conséquence des agrégats d'α-synucléine, et non des précurseurs de la MP (Jellinger, 2012; pour une revue).

Dans la MP, le volume de la matière grise est réduit, notamment celui des ganglions de la base, du mésencéphale en général et des lobes temporaux médians (Zeighami, Ulla, Iturria-Medina, Dadar, Zhang, et al., 2015). Plus précisément, il y a une atrophie au niveau de l'amygdale, de l'hippocampe et des autres structures du système limbique (Kehagia, Barker et Robbins, 2010). Également, le volume cortical en général diminue au cours de la maladie (Kehagia et al., 2010).

Anomalies neurochimiques. La perte de neurotransmetteur la plus importante dans la neuropathologie de la MP est celle de la dopamine (DA) (Dirnberger et Jahanshahi, 2013; Kehagia et al., 2010). Au moment du diagnostic, la dégénérescence des neurones DA de la substance noire *pars compacta* aurait déjà diminué drastiquement de 60% à 70% les niveaux de DA sous-corticale (Fearnley et Lees, 1991). Cette perte de DA se produirait notamment dans les ganglions de la base, plus particulièrement au niveau du striatum (Dirnberger et Jahanshahi, 2013; Kehagia et al., 2010). Il semblerait que les neurones du tiers ventrolatéral de la substance noire *pars compacta* seraient les premiers à dégénérer et qu'ensuite, la propagation se ferait vers la partie dorsale (Fearnley et Lees, 1991; Kehagia et al., 2010). Ces structures sont d'ailleurs fortement impliquées dans le contrôle sensorimoteur (Dirnberger et Jahanshahi, 2013). La dégénérescence se propagerait d'abord au niveau de la voie DA nigro-striatale, et par la suite, les structures de la voie DA mésocorticolimbique seraient aussi atteintes (Alcaro et al., 2007), contribuant à certains symptômes cognitifs chez les patients MP (Aarsland et al., 2011; Jellinger, 2006).

Toutefois, d'autres atteintes neurochimiques sont observées chez les patients et permettent d'expliquer en partie les symptômes moteurs et non-moteurs. D'abord, les atteintes cholinergiques du noyau basal de Meynert sont aussi importantes et peuvent provoquer des déficits cognitifs, notamment au niveau de la mémoire, des fonctions visuospatiales et de l'attention (Kehagia et al., 2010; Müller et Bohnen, 2013). En effet, au cours de la maladie, les projections cholinergiques du noyau basal sont atteintes, telles que celles atteignant le cortex occipital, ce qui contribue, lors des stades plus avancés, aux problèmes visuospatiaux et aux hallucinations visuelles vécus par les patients (Müller et Bohnen, 2013). D'ailleurs, des corps de Lewy sont retrouvés près du noyau basal de Meynert. Toutefois, puisque

l'acétylcholine (ACh) est aussi impliquée au niveau du système nerveux autonome, il se pourrait que les déficits cholinergiques aient aussi un rôle à jouer dans les problèmes d'instabilité posturale et les chutes (Müller et Bohnen, 2013).

De plus, les atteintes du locus coeruleus ont des conséquences sur les projections de la noradrénaline. Cette structure projette vers l'ensemble du cortex cérébral, mais reçoit également des afférences du système limbique, ce qui fait qu'au long court, les neurones noradrénergiques de ces structures seront également atteints. Certains auteurs ont d'ailleurs noté que, via des projections au cortex préfrontal, le système noradrénergique serait impliqué dans la formation des comportements adaptatifs lors d'une situation nouvelle ou stressante. L'altération de ces projections aurait donc des impacts entre autres sur la capacité de planification et d'organisation chez les patients MP (Del Tredici et Braak, 2013).

Il semblerait également qu'il y ait une dégénérescence au niveau du système de la sérotonine. Même lors des premiers stades de la maladie, une perte de neurones sérotoninergiques est observée d'une part dans le noyau du raphé, et d'autre part dans le striatum (Politis et Niccolini, 2015). Cette dégénérescence serait impliquée dans plusieurs des symptômes non-moteurs, notamment les problèmes de sommeil, les symptômes dépressifs et anxieux, pouvant être présents dès le tout début de la maladie (Politis et Niccolini, 2015).

Enfin, des dysfonctions glutamatergiques sont observées, mais il s'agirait en fait d'une conséquence des déplétions de DA et d'ACh. Ces déficits adviendraient plus tard lors des stades plus avancés de la maladie, et lors de la démence parkinsonienne (Jellinger, 2012; Kehagia et al., 2010; Lees et al., 2009).

Toutes les atteintes neurochimiques auront au long cours des répercussions sur les structures anatomiques qui y sont reliées et contribueront aux altérations neuropathologiques mentionnées précédemment.

Facteurs de risque associés à la MP

Comme pour l'étiologie de la maladie d'Alzheimer (MA), celle de la MP est encore inconnue à ce jour. La principale hypothèse envisagée serait une combinaison entre une vulnérabilité génétique et des facteurs environnementaux qui moduleraient l'ampleur de la perte neuronale (Schapira et Jenner, 2011).

Vulnérabilité génétique. La susceptibilité génétique serait impliquée dans 10 à 15% des cas de MP et donc, la majorité des cas serait plutôt de nature idiopathique (Lesage et Brice, 2009). Des mutations des gènes «*Leucine-Rich Repeat Kinase 2*» (LRRK2) et α-synucléine seraient des facteurs directs dans la forme familiale autosomique dominante de la MP (Sai, Zou, Peng, et Dong, 2012; Zimprich, Biskup, Leitner, Lichtner, Farrer, et al., 2004). Aussi, des altérations des gènes *parkin* et «*PTEN-Induced Putative Kinase 1*» (PINK1), jouant un rôle dans l'intégrité des mitochondries et dans la protection contre le stress oxydatif, seraient également liées au développement de la MP (Sai et al., 2012). Il est à noter que d'autres polymorphismes ont aussi été identifiés, mais leur contribution n'est pas encore totalement éclaircie; c'est la raison pour laquelle ils ne seront pas abordés dans le présent document.

Facteurs environnementaux. Plusieurs toxines seraient associées au développement de la MP. La substance la plus connue en lien avec la MP est le 1-méthyl-4-phényl-1,2,3,6-tétrahydropyridine (MPTP), découverte par erreur au courant des années 1970 (Cicchetti, Drouin-Ouellet, et Gross, 2009). En effet, une injection provoque chez l'humain des symptômes irréversibles semblables à ceux de la MP et aussi explicables par une perte massive de DA (Cicchetti et al., 2009; Pan-Montojo et Reichmann, 2014; Tanner, 1989). Dans le même sens, il a été remarqué que le fait de vivre en milieu rural augmente le risque de développer la MP (Pan-Montojo et Reichmann, 2014; Tanner, 1989), notamment en raison de l'exposition à des pesticides et à des métaux lourds (Pan-Montojo et Reichmann, 2014).

Pour ce qui est spécifiquement des troubles cognitifs dans la MP, d'autres facteurs de risque commencent à être investigués. En effet, il a été suggéré que la présence de facteurs de risque vasculaires tels que l'hyperhomocystéinémie et le tabagisme pouvaient être associés au développement des troubles cognitifs dans la MP (Doiron, Dupré, Langlois, Provencher et Simard, sous presse; Doiron et Simard, 2012). Toutefois, l'importance de la présence des facteurs de risque vasculaires dans le développement des troubles cognitifs et de la démence dans la MP demeure un sujet controversé.

<u>Présentation clinique de la MP</u>

Présentation motrice. Au niveau clinique, la MP est principalement diagnostiquée par la présence, chez les patients, d'altérations motrices (Hughes et al., 1992; Lees et al., 2009). En effet, même si la perte neuronale commence bien des années avant le début des symptômes, la grande majorité des cas est diagnostiquée au moment de l'apparition des symptômes moteurs. Les symptômes moteurs se

résument principalement par des tremblements au repos, unilatéraux au début de la maladie, et / ou de la rigidité musculaire (Hughes et al., 1992). On dénote aussi une bradykinésie, c'est-à-dire une lenteur dans les mouvements, une instabilité posturale ainsi qu'une micrographie, c'est-à-dire une écriture de plus en plus petite (Hughes et al., 1992; Lees et al., 2009). Un dernier point important à mentionner est une réponse positive à la Lévodopa (L-DOPA), un médicament qui agit comme précurseur de la DA (Lees et al., 2009).

Pour le diagnostic, les critères mentionnés ci-haut sont ceux de la «*United Kingdom Parkinson's Disease Brain Bank Society*» (UKPDBBS) qui sont les plus utilisés actuellement dans le monde (voir l'annexe 1.1 pour une description) (Hughes et al., 1992). D'autres critères diagnostiques ont aussi été développés par le «*National Institute of Neurological Disorders and Stroke*» aux États-Unis (NINDS; voir le tableau 1.2 de l'annexe 1) (Gelb, Oliver, et Gilman, 1999). Globalement, les critères américains sont semblables aux critères de la UKPDBBS, bien que certaines différences existent. Tandis que la bradykinésie est un critère obligatoire de la UKPDBBS, elle n'est pas nécessaire selon les critères de la NINDS (Doiron et Simard, 2012; pour une revue). De plus, parmi les critères supportant le diagnostic de MP probable, les critères du NINDS indiquent que les tremblements ou la rigidité doivent affecter plus gravement le côté du début de la maladie, alors que la UKPDBBS mentionne qu'obligatoirement, le début des symptômes doit être unilatéral (Hughes et al., 1992). Récemment, la «*Movement Disorder Society*» a révisé les critères diagnostiques de la MP (Postuma, Berg, Stern, Poewe, Olanow et al., 2015). Cette fois-ci, une vision en termes de symptômes positifs (supportant le diagnostic) et négatifs (suggérant d'autres explications) a été adoptée, et les manifestations obligatoires sont la bradykinésie, la rigidité et le tremblement au repos. Le tableau 1.3 de l'annexe 1 fournit une description détaillée de ces nouveaux critères diagnostiques.

Afin de caractériser l'évolution de la maladie d'un point de vue clinique, les stades de Hoehn et Yahr, établis en 1967 et révisés en 2004, sont généralement utilisés et permettent de classifier les patients selon leurs atteintes motrices (Goetz, Poewe, Rascol, Sampaio, Stebbins et al., 2004; voir l'annexe 2).

Présentation non-motrice.

<u>Symptômes végétatifs, autonomes et sensoriels.</u> La MP implique de nombreux symptômes non-moteurs qui ont un impact sur le fonctionnement de l'individu. Les patients atteints de la MP se plaignent

entre autres de symptômes du système nerveux autonome périphérique, tels que l’hypotension orthostatique, les problèmes gastro-intestinaux et la constipation en début de maladie, qui sont potentialisés par la médication L-DOPA (Chaudhuri et Schapira, 2009). Plus tard, lors des stades avancés, certains patients peuvent souffrir d’incontinence urinaire et fécale (Chaudhuri et Schapira, 2009). Par ailleurs, des symptômes du système sensoriel sont observés par les patients, notamment des dysfonctions olfactives (anosmie) et gustatives qui font partie des premières observations faites par les patients avant même l’apparition des symptômes moteurs (Chaudhuri et Schapira, 2009).

Symptômes psychologiques et comportementaux. Des symptômes psychologiques et comportementaux (SPC) sont aussi dénotés par les patients et leurs proches. En moyenne, chez environ 40% des patients, de l’apathie et des symptômes dépressifs ou anxieux concomitants à la MP sont retrouvés à un stade ou un autre de la maladie (Leentjens, Dujardin, Marsh, Martinez-Martin, Richard et al., 2008; Williams et al., 2012). Dans une recension des écrits, Weintraub et Burn (2011) ont estimé qu’entre 5% et 20% des patients remplissaient les critères diagnostiques de la dépression majeure selon le DSM-IV au moment du diagnostic de la MP, et qu’entre 10% et 30% des autres patients présentaient des symptômes dépressifs cliniquement significatifs. Les données les plus récentes sur le sujet vont dans le même sens: elles montrent que les patients *de novo* atteints de la MP sont deux fois plus nombreux (14% de l’échantillon) à présenter des symptômes dépressifs cliniquement significatifs que les sujets contrôles (7%) appariés selon l’âge et le niveau d’éducation (Weintraub et al., 2015). L’apathie était trois fois plus fréquente chez les patients *de novo* atteints de la MP (17% contre 5% pour les contrôles). Dans ce même échantillon, trois fois plus de patients atteints de la MP présentaient des symptômes anxieux cliniquement significatifs, comparé aux contrôles (24,6% contre 7%). Toutefois, il est difficile de savoir si ces symptômes sont une conséquence neurologique découlant des atteintes cérébrales ou bien s’il s’agit d’une réaction psychologique suite au diagnostic et à la difficulté croissante d’accomplir des tâches quotidiennes (Pirogovsky, Schiehser, Obtera, Burke, Lessig et al., 2014).

D’autres SPC incluent des hallucinations visuelles qui se présentent souvent plus tard dans l’évolution de la MP (Aarsland et al., 2009), un risque accru de développer un trouble de contrôle des impulsions (Gallagher, O'Sullivan, Evans, Lees et Schrag, 2007), ainsi que des troubles du sommeil (Adler et Thorpy, 2005). Concernant ces derniers, des études estiment que jusqu’à 90% des patients

atteints de la MP éprouveraient des problèmes de sommeil (Weintraub et Burn, 2011). En particulier, les troubles du sommeil paradoxal seraient fréquemment observés en phase prodromique de la MP, mais d’autres symptômes, comme la fatigue diurne, le syndrome des jambes sans-repos ou les terreurs nocturnes, ont aussi été rapportés (Claassen, Josephs, Ahlskog, Silber, Tippmann-Peikert et Boeve, 2010).

Il est important de noter que les SPC, en eux-mêmes, peuvent contribuer aux déficits cognitifs observés chez les patients atteints de la MP. Par exemple, un patient apathique peut présenter une altération de la vitesse de traitement de l’information, alors cela pourrait contribuer, avec les atteintes motrices, à ce que des tâches chronométrées soient moins bien réussies (Palavra, Naismith et Lewis, 2013). De plus, pour un patient déprimé, la motivation et la concentration nécessaires pour compléter une tâche cognitive peuvent être difficiles à trouver (Leentjens et al., 2008). Bien que les SPC soient nombreux et fréquents, les symptômes cognitifs demeurent parmi les plaintes les plus récurrentes et altèrent eux-aussi la qualité de vie des patients.

Le trouble cognitif léger (TCL) dans la MP

L’intérêt pour les troubles cognitifs légers dans la MP est relativement récent, car il était souvent mentionné, jusqu’au début des années 1980, que les altérations notées sur les tests cognitifs étaient causées par les problèmes moteurs rencontrés par les patients et que les cas de démence se présentaient uniquement lors des stades avancés de la MP (Sollinger et al., 2010). Par contre, il est maintenant connu que le TCL dans le cadre de la MP est non seulement considéré comme un stade intermédiaire vers le développement d’une démence parkinsonienne, mais il peut également faire partie intégrante du portrait clinique de la MP dès le début de la maladie (Litvan, Goldman, Tröster, Schmand, Weintraub, Petersen et al., 2012). En effet, des plaintes cognitives subjectives et des déficits cognitifs objectifs sont observables chez des patients nouvellement diagnostiqués, même lorsqu’ils présentent très peu d’altérations motrices (Aarsland et al., 2011). Il est à noter que selon les différentes études, entre 40% et 50% des patients rencontreraient les critères du TCL au cours de la maladie, mais chez les patients *de novo*, sans médication, l’incidence la plus probable a été établie à approximativement 27% (entre 19% et 36%; Aarsland et al., 2009). Plus récemment, une étude chez des patients parkinsoniens *de novo* a rapporté des prévalences qui varient entre 3% et 22%, dépendamment des critères

diagnostiques utilisés et de la rigueur de l'évaluation neuropsychologique (Weintraub et al., 2015). Par exemple, les études qui utilisent des mesures de fonctionnement cognitif global, comme le «*Mini-Mental State Examination*» (MMSE) ou le «*Montréal Cognitive Assessment*» (MoCA), obtiennent une prévalence plus élevée de patients atteints de TCL, par comparaison aux études effectuant une évaluation cognitive plus exhaustive en utilisant une batterie neuropsychologique standardisée (Weintraub et al., 2015).

Le TCL est défini comme une altération mineure du fonctionnement cognitif, soit dans un seul domaine cognitif (TCL à domaine simple) ou dans plusieurs domaines cognitifs (TCL à domaines multiples) (Litvan et al., 2011; Petersen, 2004; 2012; Tröster, 2011). L'arbre décisionnel de Petersen pour caractériser le TCL est retrouvé en annexe 3. Récemment, Litvan et ses collaborateurs (2012) ont établi des lignes directrices pour le diagnostic du TCL dans la MP. Subséquemment, la nomenclature utilisée dans le texte pour désigner ces patients sera «patients MP-TCL». Le tableau 1 résume ces critères. Le TCL peut être amnésique, lors d'atteintes à la mémoire, ou non-amnésique, lors d'atteintes à tout autre domaine cognitif (Litvan et al., 2012). Plusieurs études ont établi que les TCL non-amnésiques à domaine simple, avec atteintes aux fonctions exécutives, étaient plus fréquents chez les patients MP (entre 13% et 62% selon les études; Aarsland et al., 2009; Matteau, Dupré, Langlois, Provencher, et Simard, 2012) et un important prédicteur du développement d'une démence (Litvan et al., 2011). Pour ce qui est du TCL non-amnésique à domaines multiples, la prévalence atteint au maximum 50%, dépendamment des caractéristiques des patients inclus dans les études (Matteau et al., 2012). La proportion de patients MP-TCL de type amnésique varie entre 9% et 25%, que ce soit à domaine simple ou à domaines multiples (Aarsland et al., 2009; Matteau et al., 2012). Ainsi, la grande variabilité des prévalences entre les différentes études montre à quel point les profils cognitifs dans la MP sont hétérogènes (Tröster, 2011).

**Critères diagnostiques du TCL dans la MP de Litvan et al., 2012**
Diagnostic de la MP selon la *UK PD Brain Bank Society* (Hughes et al., 1992)
Déclin graduel des habiletés cognitives, tel que rapporté par le patient, son proche aidant et/ou par le clinicien, qui a procédé à l'un des deux types d'évaluation suivants.
**Évaluation neuropsychologique de type 1**<br>Altération du fonctionnement cognitif tel que mesuré par une échelle de cognition globale validée chez les patients atteints de la MP (par ex. MoCA; *Parkinson Disease Cognitive Rating Scale*; *Mattis Dementia Rating Scale*).<br>Ou;<br>Altération du fonctionnement cognitif, tel que mesuré par des résultats entre 1 et 2 écarts-types en-deça de la moyenne (normes selon l'âge et/ou le niveau d'éducation) obtenus sur au moins deux tests standardisés lors de l'administration d'une batterie neuropsychologique brève incluant un test par domaine cognitif ou évaluant moins de cinq domaines cognitifs.<br>Cette évaluation permet de poser un diagnostic possible, mais qui n'est pas aussi certain que pour l'évaluation de type 2.

**Évaluation neuropsychologique de type 2**<br>Déclin cognitif objectif, tel que mesuré par des résultats entre 1 et 2 écarts-types en-deça de la moyenne (normes selon l'âge et/ou le niveau d'éducation) obtenus sur au moins 2 tests dans un même domaine cognitif ou dans des domaines cognitifs différents parmi les suivants :<br>1) attention et mémoire de travail,<br>2) fonctions exécutives,<br>3) fonctions langagières,<br>4) mémoire (épisodique),<br>5) fonctions visuo-spatiales.<br>Deux tests neuropsychologiques par domaine (batterie d'évaluation neuropsychologique complète) doivent être effectués pour confirmer l'altération cognitive objective du participant.
Les difficultés n'interfèrent pas significativement avec les activités quotidiennes, mais des difficultés mineures sont parfois observées.

Tableau 1 : Critères diagnostiques du TCL dans un contexte de MP selon Litvan et al. (2012).

Bien qu'il soit impossible de dresser un portrait exhaustif des atteintes cognitives dans la MP, certains déficits ont été observés et répliqués par plusieurs études. Il faut de nouveau souligner ici que le profil cognitif des patients MP et MP-TCL est généralement hétérogène, donc aucune des atteintes mentionnées ci-dessous n'est systématiquement retrouvée chez l'ensemble des patients MP-TCL (Monchi, Hanganu, et Bellec, 2016).

Langage

Il est possible de diviser le langage en plusieurs composantes séquentielles, dont une est reliée à l'aspect perceptif (audition / vision), une autre à l'aspect interprétatif (compréhension / sémantique) et une dernière à l'aspect moteur (planification / production) (Vertstichel et Cambier, 2005). De par les atteintes motrices qui surviennent éventuellement au cours la MP, les patients éprouveraient des problèmes liés à la parole et à l'articulation, comme de la dysarthrie et de la dysprosodie (Poletti, Emre, et Bonuccelli, 2011a). En fait, certains auteurs ont émis un parallèle entre les patients présentant une aphasie de Broca et certains patients MP, car il semblerait qu'une production du langage soit plus difficile en début de MP, mais que la compréhension resterait intacte (Bastiaan et Lenders, 2009). Au niveau de la production verbale, ce serait la fluidité verbale, surtout lexicale, qui serait réduite dans le MP-TCL (Poletti et al., 2011a). Au niveau interprétatif, la compréhension de phrases plus complexes devient également plus difficile lors des stades plus avancés du MP-TCL et les premiers stades de la démence parkinsonienne, ce qui pourrait être relié aux atteintes des régions temporales qui surviennent plus tard, régions impliquées dans l'aspect sémantique du langage (Bastiaan et Lenders, 2009). Des problèmes dans l'utilisation adéquate des règles grammaticales pourraient aussi être présents (Bastiaan et Lenders, 2009). Une hypothèse a été émise selon laquelle ce serait la séquence nécessaire pour produire

adéquatement des mots et des phrases compréhensibles qui serait déficitaire et qui empêcherait la production du langage chez les patients MP. La capacité de planifier une séquence de langage serait par ailleurs reliée à l'activité des ganglions de la base, atteints dans la MP (Bastiaan et Lenders, 2009).

Attention

Selon le modèle de Van Zoomeren et Brouwer (1994), l'attention se diviserait en plusieurs niveaux en termes d'intensité et de sélectivité. En termes d'intensité, l'alerte phasique permet de réagir rapidement face à un stimulus non-spécifique tandis que l'attention soutenue permet de maintenir son attention durant une longue période. En termes de sélectivité, l'attention sélective permet d'ignorer les stimuli non-pertinents tandis que l'attention divisée permet à l'inverse de partager l'attention entre plusieurs stimuli. Ces niveaux sont tous supervisés par un système central : le contrôle attentionnel superviseur, un élément ajouté ultérieurement et inspiré du modèle «*Supervisory Attentional System*» (SAS) de Norman et Shallice (1986). Ce système SAS est flexible, élabore des stratégies pour atteindre un objectif et trouve des solutions en cas de situation nouvelle.

Chez certains patients MP, des fluctuations importantes sont dénotées dans le contrôle interne de l'attention ainsi que des difficultés pour des tâches demandant de l'attention sélective, soutenue et divisée (Dirnberger et Jahanshahi, 2013; Dujardin, Tard, Duhamel, Delval, Moreau et al., 2013). Par exemple, des tâches informatisées demandant de réagir le plus rapidement possible face à un stimulus quelconque, de ne réagir qu'à un type de stimuli en ignorant les autres (paradigme de Go – No Go) ou d'alterner entre deux patrons de réponses, sont souvent utilisées pour mesurer les capacités attentionnelles (Dujardin et al., 2013). Une hypothèse soulevée pour expliquer ces atteintes serait que les patients parkinsoniens auraient plus de difficultés à mobiliser leurs ressources attentionnelles lors d'une tâche non-routinière, ce qui concorderait avec les déficits d'inhibition des réponses automatiques également observés (Dirnberger et Jahanshahi, 2013; Kudlicka, Clare et Hindle, 2011). Toutefois, outre les difficultés de contrôle attentionnel relevant probablement plus de la mémoire de travail et du contrôle exécutif, les problèmes d'attention ne semblent généralement pas au premier plan dans les plaintes cognitives (Aarsland, 2016).

Mémoire de travail

En ce qui concerne la mémoire de travail, Baddeley (2010) la définit dans son modèle comme un système qui permet de maintenir et de manipuler temporairement des informations nécessaires à la compréhension, à l'apprentissage et au raisonnement. Dans la dernière version du modèle, un supra-système, l'administrateur central, coordonnerait trois sous-systèmes : la boucle phonologique, le calepin visuospatial, et le «*buffer*» épisodique (Baddeley, 2010). La boucle phonologique permet de retenir brièvement (quelques secondes) et de manipuler l'information verbale et serait impliquée dans l'apprentissage du vocabulaire et du langage. Le calepin visuospatial s'occupe quant à lui de retenir l'information visuelle, de former des images mentales et de les manipuler dans l'espace. Le «*buffer*» épisodique a été rajouté ultérieurement dans ce modèle et assure une interaction avec la mémoire à long terme. Autrement dit, il permet aux informations en mémoire à long terme de ressurgir à la conscience et il permet également de transférer l'information depuis la mémoire de travail jusqu'en mémoire à long terme, peu importe la modalité. Chez plusieurs patients MP-TCL, ce serait la manipulation d'information, et surtout d'information visuospatiale, qui serait altérée (Parnetti et Calabresi, 2006; Pagonabarraga et Kulievsky, 2012). Dans le modèle de Baddeley, cela traduirait donc des déficits aux niveaux du calepin visuospatial et de l'administrateur central. Par exemple, les patients parkinsoniens seraient sensibles aux tâches comme l'empan envers de la batterie «*Weschler Adult Intelligence Scale – IV*» (WAIS-IV), les blocs de Corsi (Pagonabarraga et Kulievsky, 2012) et le paradigme de la double tâche dans laquelle on compare la performance d'un participant lorsqu'il fait deux tâches chacune isolément avec sa performance lorsqu'il doit faire deux tâches en même temps (ex. répéter des séquences de chiffres et résoudre un labyrinthe sur une feuille de papier) (Sala, Baddeley, Papagno et Spinnler, 1995).

Mémoire épisodique

Selon le modèle Sériel, Parallèle et Indépendant (SPI) de Tulving (1995), la mémoire se sépare en plusieurs composantes et l'information serait encodée de façon sérielle, stockée de façon parallèle et récupérée de façon indépendante. Tulving fait notamment la distinction entre la mémoire épisodique, reliée à nos expériences passées en lien avec notre conscience de soi (mémoire autonoétique), et la mémoire sémantique, qui sera décrite dans la prochaine section. Ces deux types de mémoire peuvent contenir de l'information visuelle ou verbale. Trois processus seraient impliqués dans la mémoire

épisodique : l'encodage, la consolidation et la récupération d'information. L'encodage est le processus qui permet d'associer une nouvelle information à un contexte connu. La consolidation est plutôt reliée au processus de stockage de l'information. La récupération est le processus par lequel l'information encodée et consolidée est transférée depuis la mémoire à long terme jusqu'en mémoire de travail afin d'être utilisée à nouveau. Chez certains patients MP-TCL, des déficits spécifiques de la mémoire épisodique ont été rapportés. Les processus d'encodage et de consolidation seraient préservés, bien qu'ils soient plus lents que la moyenne (Barker et Williams-Grey, 2014; Monchi et al., 2016; Poletti et al., 2011a). La récupération de l'information serait déficitaire, surtout pour ce qui est du rappel libre de l'information (Pagonabarraga et Kulievsky, 2012). Des difficultés à des tâches sensibles à ces processus, comme le «*California Verbal Learning Test*» (CVLT, modalité verbale) et le «*Visual Retention Test*» (modalité visuelle), sont rapportées fréquemment dans la littérature chez les patients MP (Kehagia et al., 2010; Pagonabarraga et Kulievsky, 2012). Les déficits seraient généralement plus marqués en modalité visuelle, mais certains patients présentent plus de difficulté en modalité verbale (Pagonabarraga et Kulievsky, 2012).

<u>Mémoire sémantique</u>

Toujours selon le modèle SPI de Tulving (1995), la mémoire sémantique serait complémentaire à la mémoire épisodique et concernerait les informations verbales ou visuelles non-liées aux souvenirs autobiographiques, tel que la connaissance du monde, des objets, des évènements, tous indépendants du concept de soi (anoétique) (Tulving, 1995). Pour la mesurer, il est possible d'utiliser des tâches dans lesquelles un participant doit répondre à des questions de connaissances générales ou de classer des objets dans une catégorie sémantique; par exemple, un papillon est-il un animal, un végétal ou un objet? (Guidi, Paciaroni, Paolini, Scarpino, et Burn, 2015). Chez les patients MP-TCL, des déficits pour classer correctement un objet dans une catégorie sémantique ont été rapportés, bien que la capacité à nommer correctement les catégories sémantiques soit préservée (Guidi et al., 2015). Il a été proposé que les aspects exécutifs de la mémoire sémantique, comme la récupération et la manipulation de l'information en mémoire, étaient plus altérés que les processus d'encodage et de stockage, ce qui concorde avec les déficits rapportés en mémoire épisodique (Guidi et al., 2015).

Mémoire procédurale

Le modèle de l'«*Adaptative Control of Thought*» (ACT) de Anderson divise les informations en deux types : déclarative, liée aux faits et connaissances exprimables par le biais du langage, ou procédurale, qui porte sur la mémoire des mouvements et des routines (Anderson, 1982; Anderson et Schuss, 2000). L'apprentissage procédural se ferait en suivant trois étapes. D'abord, la phase cognitive impliquerait d'autres processus mnésiques, tels que la mémoire épisodique et la mémoire de travail. La personne apprend les étapes nécessaires à l'accomplissement de l'activité. Ensuite, durant la phase associative, à force de pratique et de répétition d'une tâche, les autres processus mnésiques s'impliqueraient de moins en moins et enfin, lors de la phase autonome, le nouvel apprentissage deviendrait automatique, et donc deviendrait une routine (Anderson, 1982; Anderson et Schuss, 2000). Selon certaines études, l'apprentissage procédural, surtout au niveau de la phase autonome, serait atteint chez certains patients MP sans altération à un autre domaine cognitif, (Doyon, Gaudreau, Laforce, Castonguay, Bedard et al., 1997; Doyon et Laforce, 2001). Par exemple, des déficits ont été rapportés chez certains patients MP pour des tâches comme la «*Serial Reaction Time*», dans laquelle on demande au participant d'associer correctement une séquence motrice à un stimulus sur un écran d'ordinateur (Doyon et al., 1997; Doyon et Laforce, 2001). Ces altérations s'expliqueraient entre autres par les atteintes du striatum, directement impliqué dans l'apprentissage d'une routine (Doyon et Laforce, 2001).

Fonctions visuospatiales

Les travaux de Boller, dans les années 1980, ont été parmi les premiers à travailler sur la caractérisation des difficultés au niveau des fonctions visuospatiales dans la MP. Les fonctions visuospatiales ont alors été définies comme étant l'ensemble des processus permettant d'estimer la position relative d'un stimulus ou d'un objet dans l'espace, d'intégrer ces objets dans une image mentale et de manipuler mentalement ces objets selon des concepts spatiaux (e.g. déplacer, retourner, faire une rotation ou une symétrie, etc.) (Boller, Passafiume, Keefe, Rogers, Morrow et al., 1984). En neurobiologie, deux voies distinctes ont été identifiées comme étant impliquées dans les fonctions visuospatiales: la voie du «*what*» et la voie du «*where*» (Kolb et Winshaw, 2008). Les deux voies partent des aires visuelles (cortex strié et extra-strié) et relaieraient l'information en parallèle. La voie du «*what*», aussi appelée ventrale, permet de reconnaître correctement un objet dans le champ de vision et

passerait par le lobe temporal médian droit pour se rendre au niveau du cortex temporal inférieur. La voie du «*where*», aussi appelée dorsale, permet de faire une image mentale de la localisation d'un objet dans l'espace et de coordonner les mouvements en fonction de cette image. Cette voie passerait plutôt par le lobe pariétal droit, mais elle se terminerait également au niveau du cortex temporal inférieur (Kolb et Winshaw, 2008). Plusieurs déficits des fonctions visuo-spatiales ont été observés et certains des déficits rapportés précédemment sont liés au traitement de l'information visuo-spatiale (Parnetti et Calabresi, 2006; Pagonabarraga et Kulievsky, 2012). Notamment, des déficits dans la perception et l'organisation de l'espace sont observés, en plus des problèmes de rotation mentale (Pagonabarraga et Kulievsky, 2012; Parnetti et Calabresi, 2006; Poletti, et al., 2011a). Plusieurs tests utilisés chez les patients MP ont démontré leur sensibilité pour évaluer ces processus. Parmi ces tests, on retrouve le test de jugement d'orientation des lignes de Benton et le test d'organisation visuelle de Hooper (Boller et al., 1984). Les déficits visuospatiaux seraient liés au développement subséquent d'hallucinations visuelles (Pagonabarraga et Kulievsky, 2012). Des auteurs ont noté que le noyau caudé dorsal, atteint tôt dans le développement de la MP, est anatomiquement très proche et connecté au lobule pariétal inférieur droit, impliqué dans la cognition spatiale (Kesner et Creem-Regehr, 2013). Cela laisse donc entendre que, tôt dans le développement de la MP, la voie du «*where*» serait atteinte, mais ce ne serait pas nécessairement le cas pour la voie du «*what*».

Fonctions exécutives

Les fonctions exécutives sont des habiletés mentales de haut niveau servant à l'organisation, à la planification, à l'intégration de plusieurs modalités sensorielles, à l'inhibition de comportements automatiques et à la résolution de problèmes (Smith et Jonides, 1999). Elles permettraient de mobiliser les autres fonctions cognitives et de les coordonner lors d'actions dirigées vers un but. Plusieurs modèles théoriques tentent d'expliquer les fonctions exécutives.

Un premier modèle bien connu est celui de Norman et Shallice (1986), qui ont proposé un modèle hiérarchique du contrôle de l'action, divisé en trois composantes. La première est le répertoire des schémas d'action, qui permet facilement de planifier les activités déjà apprises et qui demande un niveau d'attention minimal. Ce niveau de fonctionnement se rapproche de la conception de la phase d'automatisation de la mémoire procédurale de Anderson. La deuxième composante est le gestionnaire

de conflits entre les schémas, qui sélectionne le schéma le plus approprié à une situation lorsque deux schémas ou plus sont activés en même temps. La troisième est le système superviseur («*Supervisory Attentional System*» ou SAS), qui gère les situations nouvelles, peu routinières, ou lorsque les schémas connus ne sont pas adaptés. Ce serait le système superviseur qui demanderait le plus de ressources attentionnelles et qui s'occuperait de la planification, de la prise de décision, de la résolution de problèmes et de l'inhibition, entre autres.

Deuxièmement, selon la théorie de Duncan (1986), des lésions frontales, peu importe leur origine, entraînent des déficits généraux dans les actions dirigées vers un but, ce qui explique la grande variété de difficultés que les patients lésés peuvent vivre au quotidien. Duncan (1986) décrit ces déficits comme étant des dysfonctions exécutives, c'est-à-dire un problème d'organisation de la pensée et de l'action. Toujours selon sa théorie, les séquences nécessaires pour accomplir un objectif seraient fragmentées ou incomplètes, ce qui entraîne des omissions (de séquences essentielles à la tâche) ou des intrusions (de séquences non-essentielles à la tâche).

Il a été noté que même chez des patients ne présentant pas cliniquement la MP, mais étant génétiquement à risque, les fonctions exécutives seraient altérées (Hawkins, Jennings, Marek, Siderowf et Stern, 2010). Chez les patients MP-TCL qui présentent des atteintes aux fonctions exécutives, des problèmes d'inhibition des réponses automatiques, tels que mesurés par la tâche de Stroop, seraient souvent observés (Kudlicka et al., 2011; Kehagia et al., 2010). Aussi, les patients présentent des déficits dans l'alternance des patrons de réponse ou au niveau de la capacité de flexibilité mentale («*set shifting*»), tel que mesuré par la condition d'alternance des tests de fluidité verbale. La capacité de résolution de problèmes, ainsi que la vitesse de prise de décision seraient aussi déficitaires (Dirnberger et Jahanshahi, 2013; Kehagia et al., 2010; Kudlicka et al., 2011). Pour les patients parkinsoniens, les actions dirigées vers des buts sont donc plus difficiles à accomplir, depuis les étapes de planification jusqu'aux actions concrètes pour y parvenir (Dirnberger et Jahanshahi, 2013; Kehagia et al., 2010). En se basant sur le modèle de Norman et Shallice, certains auteurs ont proposé que les systèmes demandant une attention minimale seraient atteints dans la MP (e.g. le répertoire de schémas routiniers et le gestionnaire de conflits entre les schémas). Ainsi, même pour les tâches routinières, les patients MP devraient utiliser plus de ressources cognitives en faisant appel au système superviseur (Dirnberger et

Jahanshahi, 2013). En se basant sur le modèle de Duncan, certains patients MP présenteraient des dysfonctions exécutives, notamment pour ce qui est de coordonner toutes les actions nécessaires à l'accomplissement d'un objectif, en raison des atteintes striato-frontales.

Théorie de l'esprit

La théorie de l'esprit peut-être définie comme la capacité à comprendre et à prédire les comportements d'une autre personne en lui attribuant des états mentaux indépendants des siens (Bora, Walterfang et Velakoulis, 2015; Poletti, Enrici, Bonuccelli, et Adenzato, 2011b; Premack et Woodruff, 1978). Puisque la perception et la réaction aux stimuli sociaux demandent une interaction complexe entre émotion et cognition, plusieurs structures différentes, corticales et limbiques, telles que la jonction pariéto-temporale, le cortex préfrontal (dorsolatéral et ventromédian) et l'amygdale seraient impliqués dans ces processus (Freedman et Stuss, 2011). Afin de mesurer la théorie de l'esprit chez l'adulte, plusieurs tests standardisés sont utilisés en recherche. Ceux-ci peuvent être cognitifs (en lien avec le traitement des pensées), affectifs (en lien avec le traitement des émotions) ou les deux. D'abord, le «*First Order False Belief Test*» et le «*Second Order False Belief Test*» permettent respectivement de vérifier si un participant est capable de comprendre qu'une autre personne peut avoir une vision erronée du monde et si un participant est capable d'imaginer la réflexion qu'une autre personne peut faire (Poletti et al., 2011b). Une autre tâche souvent utilisée est celle du «*Reading the Mind in the Eyes*». Dans ce test, on demande à un participant de deviner ce qu'une autre personne, montrée sur une photo, pense et ressent. Il s'agit donc d'une tâche plus centrée sur les émotions (Poletti et al., 2011b). Enfin, un dernier test souvent rapporté dans les études est celui du «Faux pas» qui consiste à raconter 10 histoires dans lesquelles une personne fait une maladresse subtile, mais importante, ainsi que 10 histoires contrôles, sans faux pas; le participant doit expliquer correctement la maladresse lorsque présente (Poletti et al., 2011b).

Chez les patients parkinsoniens, même en début de maladie, la cognition sociale et la théorie de l'esprit seraient déficitaires (Dirnberger et Jahanshahi, 2013; Kudlicka et al., 2011; Poletti et al., 2011b). Plusieurs études menées chez des patients MP à qui la tâche de Faux pas a été administrée ont montré que la composante cognitive était atteinte, mais pas la composante affective, en l'absence de d'autres déficits cognitifs (Kawamura et Koyama, 2007; Péron, Vicente, Leray, Drapier, Drapier et al., 2009; Roca,

Torralva, Gleichgerrcht, Chade, Arévalo et al., 2010). Une récente méta-analyse sur le sujet a montré que comparés à des contrôles en santé, les patients MP réussissaient moins bien les trois tâches mentionnées ci-haut, avec une taille d'effet élevée (toutes tâches confondues, *d* de Cohen = 0.83). Plus particulièrement, les tâches cognitives comme les «*First and Second Order False Belief Tests*» obtenaient les tailles d'effet les plus élevées (*d* de Cohen = 1.26; Bora et al., 2015). D'ailleurs, les déficits étaient moins marqués en début de maladie et empiraient avec le temps.

Une hypothèse a été émise selon laquelle les atteintes des circuits frontaux-striataux, particulièrement la boucle préfrontale dorsolatérale, contribueraient aux difficultés de certains patients MP lors des tests mesurant la théorie de l'esprit (Bora et al., 2015; Poletti et al., 2011b). Un dernier aspect intéressant qui ressort des études sur la théorie de l'esprit est que les fonctions exécutives telles que mesurées par la condition d'alternance du test de fluidité verbale étaient un bon prédicteur des résultats aux tests de théorie de l'esprit (Bora et al., 2015). Une étude a même montré qu'un programme d'entraînement cognitif, ciblant la mémoire et les fonctions exécutives, mené chez des patients MP-TCL, améliorait la performance à une tâche de théorie de l'esprit basée sur le même modèle que la tâche du Faux Pas (Pena, Ibarretxe-Bilbao, García-Gorostiaga, Gomez-Beldarrain, Díez-Cirarda et Ojeda, 2014). En effet, il existe d'une part plusieurs substrats anatomiques communs entre les fonctions exécutives et la théorie de l'esprit et, d'autre part, ces fonctions sont toutes les deux reliées à la conscience de soi, très importante dans la coordination d'actions et de séquences planifiées. Quant à savoir si les dysfonctions exécutives n'expliqueraient pas entièrement les déficits de la théorie de l'esprit chez les patients MP, c'est un point qui est encore matière à débat dans la littérature (Bora et al., 2015; Freedman et Stuss, 2011).

<u>Sommaire des tests neuropsychologiques fréquemment utilisés</u>

Le tableau 1.4 de l'annexe 1 montre différents tests pouvant être utilisés et qui sont jugés comme étant assez sensibles pour l'évaluation du TCL dans la MP (la liste n'est toutefois pas exhaustive).

<u>Activités de la vie quotidienne</u>

Les activités de la vie quotidienne (AVQ) sont séparées en deux types : les AVQ de base, servant à répondre principalement aux besoins physiologiques de l'individu, tels que se nourrir ou se laver, ainsi que les activités instrumentales de la vie quotidienne (AIVQ). Ces dernières sont définies comme étant

des activités complexes, nécessaires au bon fonctionnement quotidien. Elles incluent par exemple des tâches comme l'entretien ménager, la gestion efficace des médicaments ou la communication par téléphone (Shulman, Pretzer-Aboff, Anderson, Stevenson, Vaughan, Gruber-Baldini et Weiner, 2006).

Bien qu'aucune altération majeure du fonctionnement quotidien ne soit retrouvée dans le MP-TCL, la présence d'un TCL peut engendrer des difficultés mineures dans la réalisation des AIVQ (Rosenthal et al., 2010) et affecter par le fait même la qualité de vie des patients et de leurs proches (Dirnberger et Jahanshahi, 2013; Lawson et al., 2014; 2016; Schrag, Jahanshahi et Quinn, 2000). Chez des patients MP (stades II et III de Hoehn et Yahr), certaines de ces activités deviennent plus difficiles à accomplir, tel que faire le ménage et gérer les médicaments (Schulman, Gruber-Baldini, Anderson, Vaughan, Reich, Fishman et Weiner, 2008). Une étude a aussi rapporté que l'altération des fonctions exécutives, plus particulièrement la difficulté de planifier, permettait de prédire significativement la difficulté de réaliser des AIVQ dans la MP (Cahn, Sullivan, Shear, Pfefferbaum, Heit et Silverberg, 1998). De plus, une autre étude a montré que les patients MP auraient tendance à surestimer leur fonctionnement pour quatre tâches des AIVQ, soit la gestion des médicaments, l'alimentation, l'habillement et la gestion de l'argent, lorsque leur auto-évaluation est contrastée avec l'évaluation objective réalisée par un clinicien (Shulman et al., 2006).

L'accomplissement des AIVQ, et donc indirectement l'autonomie des patients MP, semblent reliés au fonctionnement exécutif. Toutes ces composantes seraient pertinentes lors de l'évaluation d'un patient. Afin d'aider le plus longtemps possible les patients atteints de MP à demeurer autonomes, quelques traitements pharmacologiques et symptomatiques sont offerts. Ils ont démontrés une certaine efficacité pour traiter certains symptômes pendant quelques années. Les différentes options de traitements offerts seront décrites plus en détails dans la prochaine section.

Traitements de la MP

Les antiparkinsoniens

Afin de contrôler les symptômes moteurs de la maladie, le principal mécanisme d'action utilisé aujourd'hui consiste à compenser la perte de DA par des agents pharmacologiques. L'un des principaux agents antiparkinsoniens utilisé est la L-DOPA (lévodopa-bensérazide; Prolopa®; lévodopa-carbidopa; Sinemet®), un précurseur direct de la DA qui peut franchir aisément la barrière hémato-encéphalique,

contrairement à la dopamine qui ne peut la franchir (Jellinger, 2012; Poletti et Bonuccelli, 2013).La L-Dopa doit être associée avec un inhibiteur de la DOPA-Carbaoxylase, comme le benseraside du Prolopa ou la carbidopa du Sinemet qui permet de diminuer la conversion de la L-Dopa en dopamine en périphérie et faire en sorte que plus de L-Dopa traverse la barrière hémato-encéphalique..Cette co-médicamentation est efficace pour diminuer les symptômes de tremblements et de rigidité. (Connolly et Lang, 2014). Toutefois, la période d'efficacité maximale n'est en moyenne que de 5 ans à partir du diagnostic, puis l'efficacité diminue graduellement par la suite (Connolly et Lang, 2014; Poletti et Bonuccelli, 2013). Des périodes de fluctuations dans l'efficacité des antiparkinsoniens se développent éventuellement, appelées périodes ON et OFF (Connolly et Lang, 2014). D'autres agents antiparkinsoniens présentent d'autres mécanismes d'action, tels que les agonistes DA (bromocriptine, Parlodel®; pramipexole, Mirapex®; ropinirole, ReQuip®), les inhibiteurs de la monoamine oxydase B (IMAO-B; sélégiline, Eldepryal® ou rasagiline, Azilect®) et les inhibiteurs de la Catéchol-O-Méthyltransférase (COMT; entacapone, Comtan®). La COMT est un enzyme qui dégrade la DA dans l'espace synaptique (Poletti et Bonuccelli, 2013). Son inhibition permet donc à la DA de demeurer plus longtemps dans l'espace synaptique.

Toutefois, les effets de ces médicaments sur la cognition sont pour l'instant controversés (Robbins et Cools, 2014). Certaines études ont noté des améliorations cognitives chez leurs patients, notamment au niveau des fonctions exécutives et de la mémoire de travail, alors que d'autres études ont plutôt dénoté le contraire (Liepelt-Scarfone, Gräber, Fruhmann Berger, Feseker, Baysal, Csoti et al., 2012; Poletti et Bonuccelli, 2013; Robbins et Cools, 2014). En fait, il semblerait que certaines fonctions cognitives, telles que la capacité d'inhibition, la flexibilité mentale, l'attention divisée et la mémoire de travail soient sensibles à la médication DA via les circuits striataux-préfrontaux (Kehagia et al., 2010). À l'inverse, certaines fonctions visuo-spatiales, comme la capacité de rotation mentale et la mémoire visuo-spatiale ne seraient pas affectées par la déplétion de la DA, ce qui expliquerait les différences retrouvées dans les différentes études (Kehagia et al., 2010). Une méta-analyse de 2013 sur le sujet a aussi montré qu'en début de maladie, la majorité des patients bénéficiait de la médication antiparkinsonienne sur le plan cognitif, mais qu'une grande hétérogénéité s'installait dans les stades plus avancés de la maladie, et

variait entre autres selon les effets ON-OFF de la médication après quelques années d'administration (Poletti et Bonnucelli, 2013).

Les nootropes

En plus des atteintes DA caractéristiques de la MP, des atteintes cholinergiques sont également observées (Müller et Bohnen, 2013). Afin de contrer ces symptômes, le traitement est le même que pour la MA : il s'agit d'inhibiteurs de cholinestérases (IChE). L'acétylcholinestérase (AChE) et la butyrylcholinestérase (BChE) sont des enzymes servant habituellement à dégrader l'ACh dans l'espace synaptique. L'objectif des IChE est donc d'augmenter la quantité d'ACh disponible dans la fente synaptique (Rolinski, Fox, Maidment, et McShane, 2012).

Un premier IChE, le donepezil (Aricept®), inhibe l'AChE de manière réversible (Cheewakriengkrai et Gauthier, 2013). Différentes études ont récemment publié des résultats positifs quant à son efficacité pour le traitement de la démence dans la MP (Pagano et al., 2015; Rolinski et al., 2012; Wang et al., 2015). Toutefois, le donepézil n'est pas approuvé officiellement par Santé Canada pour le traitement des troubles cognitifs dans la MP.

Un deuxième IChE, la rivastigmine (Exelon®), est approuvé officiellement par Santé Canada pour le traitement de la démence parkinsonienne, donc il ne serait efficace que pour les symptômes cognitifs plus handicapants et dérangeants. Il s'agit d'un inhibiteur non-compétitif à la fois de l'AChE et de la BChE qui n'est presque pas biotransformé (Stahl, 2008).

Ensuite, la galantamine (Reminyl®) est un inhibiteur compétitif et sélectif de l'AChE, mais il est également un modulateur allostérique des récepteurs cholinergiques nicotiniques (Stahl, 2008). Dans la MP avec démence, quelques études ont porté sur la galantamine, mais les résultats ne semblaient pas positifs (Connolly et Lang, 2014; pour une revue).

Le dernier médicament approuvé pour le traitement de la MA a un mécanisme d'action quelque peu différent. La mémantine (Ebixa®) cible principalement les canaux voltage-dépendants des récepteurs N-méthyl-D-aspartate (NMDA) du glutamate. La mémantine est en effet un antagoniste non-compétitif de ces canaux, ce qui diminue la fixation du glutamate sur les récepteurs NMDA et donc, il y aurait un effet amenuisant sur l'excitotoxicité des neurones glutaminergiques (Stahl, 2008). Très peu d'études ont été

menées chez des patients MP avec la mémantine, dont aucune chez les patients MP-TCL (Connolly et Lang, 2014).

Ainsi, pour ce qui est du traitement du TCL dans la MP, aucun médicament n'est actuellement approuvé ni par Santé Canada, ni par la «*Food and Drug Administration*» (FDA) américaine. Étant donné cet état de fait, de nombreux chercheurs dans le domaine ont noté que des approches non-pharmacologiques pourraient améliorer le fonctionnement quotidien des patients actuellement aux prises avec un TCL (Hindle, Petrelli, Clare, et Kalbe, 2013; París et al., 2011; Petrelli, Kaesberg, Barbe, Timmermann, Fink, Kessler et Kalbe, 2014; Petrelli, Kaesberg, Barbe, Timmermann, Rosen, Fink et al., 2015; Zimmermann, Gschwandtner, Benz, Hatz, Schindler, Taub, et Fuhr, 2014). En effet, enseigner de nouvelles stratégies cognitives pour compenser les déficits pourrait répondre aux principales plaintes cognitives que les patients atteints de la MP expriment souvent (Angelucci, Peppe, Carlesimo, Serafini, Zabberoni, Barban, et al., 2015; Costa, Peppe, Serafini, Zabberoni, Barban et al., 2014; Hindle et al., 2013; Milman, Atias, Weiss, Mirelman, et Hausdorff, 2014; Mohlman, Chazin, et Georgescu, 2011; Nombela, Bustillo, Castell, Sanchez, Medina, et Herrero, 2011; Sammer, Reuter, Hullmann, Kaps, et Vaitl, 2006; Reuter, Mehnert, Sammer, Oechsner, et Engelhardt, 2012). Au cours des dernières années, les approches non-pharmacologiques ont éveillé l'intérêt de nombreux chercheurs et le nombre de publications portant sur ces approches dans la MP a explosé depuis 2010. En dépit du fait que les recherches se poursuivent dans l'industrie pharmaceutique, les avenues non-pharmacologiques constitueraient un bon complément pour ces patients qui prennent souvent déjà plusieurs types de médicaments. Les approches non-pharmacologiques, et plus particulièrement les interventions cognitives, seraient moins risquées en termes d'effets adverses et moins coûteuses pour le système de santé.

Les interventions cognitives

Les premières interventions cognitives étaient surtout offertes aux patients atteints de la MA (Grandmaison et Simard, 2003; Huckans et al., 2013; Huntley, Gould, Liu, Smith, et Howard, 2015), mais de plus en plus de programmes d'entraînement cognitif sont maintenant testés dans la MP. Le rationnel sous-tendant ces interventions est relativement le même que pour la MA, c'est-à-dire qu'elles permettraient d'augmenter la réserve cognitive de l'individu (Hindle, Martyr et Clare, 2014; Poletti et al.,

2011a) et d’améliorer la plasticité cérébrale (Poletti et al., 2011a). Cela pourrait donc contribuer à ralentir le déclin des symptômes cognitifs dans la MP (Hindle et al., 2013; Hindle, Martyr et Clare, 2014). Le principe de réserve cognitive est défini comme étant la capacité à optimiser la performance d’une personne en utilisant efficacement les connexions du cerveau (Stern, 2002). La plasticité cérébrale serait une propriété qui permettrait au cerveau de compenser des déficits structurels grâce à d’autres structures qui ne sont pas endommagées, ce qui normaliserait la performance d’un individu présentant un dysfonctionnement cognitif (Dirnberger et Jahanshahi, 2013; Poletti et al., 2011a). Il existe actuellement trois types d’interventions cognitives, soit la stimulation cognitive, l’entraînement cognitif et la réadaptation cognitive. Également, quelques rares études ont tenté de combiner une intervention cognitive avec de l’exercice physique, mais les résultats n’ont pas montré de changement sur une échelle globale de cognition (Reuter et al., 2012).

Stimulation cognitive

La stimulation cognitive est une intervention menée individuellement ou en petits groupes, qui se fait généralement sous forme de jeux ou de discussions. Le but est d’encourager la réflexion et l’activation mentale. Ces interventions ne sont donc pas adaptées aux déficits spécifiques du patient et l’effet recherché sur la cognition est plutôt global (Woods, Aguirre, Spector et Orrell, 2012; Huntley et al., 2015). Dans la MP, une seule étude a porté sur la stimulation cognitive (Nombela et al., 2011). Durant six mois, 10 contrôles en santé et 10 patients atteints de la MP et rapportant des plaintes cognitives subjectives devaient remplir un petit sudoku et rencontrer un psychologue une fois par semaine pour la correction. Le groupe des patients MP était séparé en deux : cinq patients ont complété le programme avec le sudoku et les cinq autres n’ont accompli aucune activité. Les résultats ont montré que la performance au test de Stroop était améliorée chez les patients MP entraînés au sudoku et que l’activation cérébrale mesurée par imagerie par résonnance magnétique fonctionnelle (IRMf) était moins élevée dans le groupe expérimental comparé avec des patients parkinsoniens contrôles, donc que la tâche demandait moins de ressources chez le groupe entraîné comparé au groupe contrôle. Les auteurs ont conclu que les exercices proposés permettaient d’optimiser les ressources cognitives des patients (Nombela et al., 2011). Cependant, les aptitudes acquises par les patients ne pouvaient être transférées à d’autres

domaines cognitifs ou aux AIVQ, et aucune mesure n'était prévue pour la qualité de vie, ce qui constitue les principales limites de cette étude.

Réadaptation cognitive

Il s'agit d'une approche individualisée, centrée sur les besoins du patient. La réadaptation cognitive peut s'adresser autant à des patients avec une démence légère, que modérée (Thivierge, Simard, Jean, et Grandmaison, 2008). Ce type d'intervention peut se faire dans le milieu de vie du patient et cibler des tâches concrètes, axées sur les AIVQ, mais peut aussi être administré en milieu hospitalier. Deux types de stratégies ou de techniques cognitives validées en laboratoire sont généralement employées : les stratégies compensatoires et les stratégies restauratrices (Buschert, Bokde, et Hampel, 2010). Les premières visent à apprendre une nouvelle manière d'accomplir une tâche, alors que les deuxièmes ont pour but de rétablir le fonctionnement cognitif déficitaire à l'aide de techniques cognitives développées en laboratoire (Buschert et al., 2010). Il existe peu d'études avec un devis méthodologique rigoureux, mais les études pilotes avec des patients atteints de la MA (stades légers à modérés) montrent des bénéfices possibles (Clare et al., 2010; Thivierge et al., 2008; Thivierge et al., 2014). Dans la MP, aucune étude n'a porté à date sur ce type d'intervention.

Entraînement cognitif

L'entraînement cognitif a pour but de maintenir ou d'améliorer certaines fonctions cognitives par le biais de la pratique répétée de tâches le plus souvent sur ordinateur, avec des techniques cognitives validées en laboratoire. Généralement, les sessions d'entraînement sont effectuées en petits groupes. Chez des patients MP, sans regard pour le statut cognitif, la majorité des études d'intervention cognitive porte sur des techniques d'entraînement cognitif, mais les méthodologies employées sont très variables, ce qui restreint l'interprétation des résultats (Angelucci et al., 2015; Edwards et al., 2013; Milman et al., 2014; Mohlman et al., 2011; París et al., 2011; Peña et al., 2014; Petrelli et al., 2014; Sammer et al., 2006; Zimmermann et al., 2014). Une première méta-analyse sur des patients MP, indépendamment de leur statut cognitif (Calleo, Burrows, Levin, Marsh, Lai et York, 2012), a inclus quatre études s'intéressant à des programmes d'entraînement cognitif. Par contre, étant donné le peu de participants dans les études incluses (n variant entre 14 et 33 selon les études) et la variabilité des différents programmes

d'entraînement, en terme de fréquence des sessions, de durée des sessions et de contenu des programmes d'entraînement, aucune analyse quantitative n'a été réalisée. Les auteurs ne peuvent pas conclure que les programmes d'entraînement cognitif sont efficaces, mais constatent qu'ils sont faisables et tolérables (Calleo et al., 2012).

Une deuxième méta-analyse de 2013 sur le sujet a cette fois-ci montré que les études sur l'entraînement cognitif avec un devis expérimental rigoureux présentaient des résultats positifs, mais une seule étude avec devis randomisé – contrôle n'est pas une donnée probante suffisante pour conclure à la présence ou l'absence de bénéfices sur la cognition (Hindle et al., 2013; París et al., 2011). D'ailleurs, aucune étude n'a montré d'amélioration sur une mesure de fonctionnement cognitif global (e.g. MMSE).

Une dernière méta-analyse plus récente (Leung, Walton, Hallock, Lewis, Valenzuela et Lampit, 2015) a calculé une taille d'effet pour les résultats des sept études qu'elle a incluses. Le *g* de Hedges a été estimé à $g = 0.23$, $p = 0.037$, ce qui traduit une taille d'effet petite, mais significative. Par contre, le statut cognitif des participants n'est pas décrit, il pouvait donc s'agir de patients MP sans TCL, MP-TCL ou MP atteints de démence. Les quelques études d'entraînement cognitif dans la MP seront décrites plus précisément, car chacune a été analysée par l'une des trois méta-analyses mentionnées ci-haut.

Patients MP sans trouble cognitif, ou avec plaintes cognitives subjectives. Quelques études montrent des résultats intéressants chez des patients atteints de la MP sans trouble cognitif objectif, mais qui, dans certains cas, se plaignent de difficultés sur le plan cognitif. D'abord, l'étude de série de cas de Mohlman et ses collaborateurs (2011) cherchait à valider la faisabilité et la tolérabilité d'un programme d'entraînement cognitif informatique, l'«*Attention Process Training*», qui est complété en quatre séances de 90 minutes sur quatre semaines. Ils ont administré ce programme à 16 patients MP sans trouble cognitif, mais n'ont pas effectué de suivi après la mesure post-entraînement immédiate. Les résultats ne mentionnent pas d'amélioration significative au plan cognitif, mais les critères de tolérabilité étaient généralement positifs aux niveaux de la fatigue ressentie par les patients, de l'effort à fournir pendant les sessions d'entraînement, de l'amusement ressenti et de la perception de progrès. Ces construits étaient évalués à la fin de chaque semaine via une feuille spécifique comprise dans le matériel du programme et qui contenait des questions avec une échelle de type Likert pour chaque construit. La conclusion de cette

étude est donc que ce programme est bien toléré et faisable avec des patients atteints de la MP, mais il faudrait davantage d'études pour confirmer l'amélioration de la cognition (Mohlman et al., 2011).

L'étude de Zimmerman et ses collaborateurs (2014) a tenté quant à elle de comparer deux programmes d'entraînement : un premier groupe de patients a reçu un programme informatisé spécifique à la cognition (fonctions d'attention, de mémoire de travail et de planification) et un deuxième groupe a reçu programme non spécifique à la cognition, mais qui impliquait une composante motrice (i.e. jouer à la console Nintendo Wii). Tous les patients ont participé à trois séances supervisées de 40 minutes par semaine, durant 4 semaines, mais aucune évaluation de suivi n'a été effectuée après la mesure post-entraînement immédiate. Les résultats n'ont montré aucune différence significative entre les groupes à la fin de l'entraînement pour aucun des domaines cognitifs évalués, bien qu'il y ait eu une amélioration chez le groupe entraîné avec la Wii entre la mesure d'attention pré et post-intervention («*Test of Attentionnal Performance*»). Les auteurs concluent que le programme avec la Wii est moins dispendieux et amuserait plus les patients qu'un logiciel informatique d'entraînement cognitif, d'autant plus que les bénéfices au plan cognitif seraient semblables entre les deux approches.

Enfin, l'étude randomisée – contrôle actif de Pena et ses collaborateurs (2014) a testé un programme d'entraînement cognitif de type «tâches papier – crayon», adapté à la mémoire, à l'attention et aux fonctions exécutives, qui s'étendait sur une durée de 12 semaines, à raison de trois séances de 60 minutes par semaine. Les auteurs ont séparé 42 participants en deux groupes : 20 participants ont suivi le programme et 22 participants ont fait des activités en groupe (lecture, dessins, cartes, etc.). Les résultats montrent une amélioration significative inter-groupe en faveur du groupe expérimental pour la vitesse de traitement de l'information, telle que mesurée par le TMT – A, de la mémoire verbale, mesurée par le «*Hopkins Verbal Learning Test*» et de la mémoire visuelle, telle que mesurée par le «*Brief Visual Memory Test*». De plus, des améliorations significatives ont été remarquées pour ce qui est de la théorie de l'esprit, mesurée par la tâche de Happé (de type cognitive). Il y avait également des améliorations dans les déficits fonctionnels, mesurés par la version courte du questionnaire «*World Health Organization Disability Assessment Schedule*». Au niveau cognitif, ce sont les mêmes tests qui ont été utilisés en pré-intervention et en post-intervention, donc il se pourrait qu'un effet de pratique ait contribué à l'amélioration

des participants sur ces mesures. Encore une fois, aussi, les auteurs n'ont pas effectué d'évaluation de suivi après les mesures post-intervention immédiates avec les participants.

En résumé, plusieurs programmes d'entraînement différents ont été évalués chez des patients parkinsoniens sans trouble cognitif et les méthodologies sont également très différentes. Les programmes ont été bien tolérés par les participants; très peu d'effets adverses ont été rapportés. La dernière étude parue à ce jour sur le sujet est celle de Petrelli et al. (2015). Il s'agit de l'étude avec le plus long suivi à ce jour (un an après la fin de l'entraînement). Les auteurs ont comparé deux programmes d'entraînement; l'un est structuré et l'autre non-structuré, avec un groupe contrôle (passif, de type liste d'attente). Leur échantillon était composé de patients MP sans TCL : 24 participants dans le groupe avec entraînement structuré (groupe 1), 24 participants dans le groupe avec entraînement non structuré (groupe 2) et 21 participants dans le groupe contrôle (groupe 3). L'entraînement cognitif consistait en 12 séances de 90 minutes réparties sur six semaines. Pour le groupe 1, il s'agissait d'exercices structurés ciblant des fonctions cognitives précises (mémoire, attention, fonctions exécutives, sans plus de détails de la part des auteurs), couplés à des séances de psychoéducation sur ces fonctions. Pour le groupe 2, l'entraînement consistait en de petits exercices informatisés non structurés (e.g. surtout des jeux), sans psychoéducation. Leurs mesures d'efficacité pré- et post-intervention étaient les résultats à des échelles de cognition globale (MMSE et Dem-Tec) ainsi que le développement subséquent ou non d'un TCL. Les résultats montrent que, même un an après la fin du programme d'entraînement cognitif, le risque de développer un TCL était réduit de moitié pour les participants des deux groupes entraînés. Pour ce qui est des résultats aux mesures cognitives, les deux programmes d'entraînement ne permettaient pas d'augmenter la performance au MMSE, mais seule la performance des participants du programme structuré ne déclinait pas. À l'inverse, celle des participants des deux autres groupes déclinait avec le temps. Ainsi, même un an après la fin de l'entraînement, les patients bénéficient encore des acquis qu'ils avaient faits, surtout en terme de prévention du déclin cognitif.

Patients MP, sans précision sur le statut cognitif. D'autres études ont inclus des participants atteints sur le plan cognitif, mais sans préciser s'ils présentaient un TCL ou une démence parkinsonienne. Le seuil critère pour l'inclusion des participants dans les études était établi à un score minimum de 24 ou de 27 au MMSE. La première étude parue dans cette section est celle de Sammer et ses collaborateurs

(2006) (score moyen au MMSE de 27,15). Ils ont recruté 26 patients atteints de la MP qui étaient hospitalisés et les ont divisés en deux groupes : 12 patients ont reçu un entraînement cognitif au cours de leur séjour à l'hôpital tandis que 14 autres patients n'en ont pas reçu. L'entraînement cognitif consistait en 10 séances de 30 minutes d'activités telles que des tâches de Casse-tête (de la WAIS-IV) ou des exercices stimulant les fonctions exécutives (e.g. répétition des tâches de la batterie «*Behavioral Assessment Dysexecutive Syndrome*» (BADS) suivantes : estimation du temps, «*Zoo Map Test*» et recherche de clés). Les résultats montrent que le groupe entraîné s'améliorait plus que le groupe contrôle à d'autres tâches de la batterie BADS que celles mentionnées ci-haut mesurant les fonctions exécutives, notamment la tâche des six éléments, même si le groupe contrôle s'améliorait aussi. Aucune mesure du transfert des acquis dans la vie quotidienne n'a été faite. Suite à la mesure post-intervention immédiate, les participants n'ont pas été suivis (Sammer et al., 2006).

L'étude d'Edwards et ses collaborateurs (2013) a évalué un programme informatisé axé sur la vitesse de traitement de l'information, qui totalisait une durée de 20 heures étalées sur 12 semaines, que les participants complétaient eux-mêmes sans supervision d'un thérapeute, selon leur horaire. L'étude a inclus 74 participants répartis en deux groupes : 32 participants dans le groupe entraîné et 42 dans le groupe contrôle passif. La mesure d'efficacité primaire est la tâche «*Useful Field of View*», une tâche estimant la vitesse de traitement de l'information. Les résultats montrent une amélioration significative à cette mesure entre la mesure pré et la mesure post-entraînement, mais il n'y a aucune différence significative pour ce qui est du fonctionnement cognitif général tel que mesuré par le «*Cognitive Self Report*», un questionnaire auto-rapporté, et des symptômes dépressifs tels que mesurés par la «*Center for Epidemiological Studies Depressive Scale*» (CES-D). Cela laisse entendre que les effets de l'entraînement ne seraient pas transférables au fonctionnement quotidien tel qu'il peut être évalué par le questionnaire auto-rapporté (Edwards et al., 2013).

Dans une autre étude publiée avec devis quasi expérimental (un seul groupe de 18 patients MP avec mesures pré- et post-intervention), Milman et ses collaborateurs (2014) ont aussi évalué un programme d'entraînement informatique. Ce dernier était adapté à la base pour des patients adultes avec trouble déficitaire de l'attention (TDA). Ce programme est axé sur les fonctions exécutives et il se complète à raison de trois séances de 30 minutes par semaine durant 12 semaines. Les fonctions exécutives ont été

mesurées avec des versions informatisées de tâches classiques telles que le test de Stroop et un paradigme «Go – No Go». Les résultats montrent une amélioration significative des participants entre les évaluations pré- et post-intervention à ces tâches, mais aussi une amélioration de la vitesse d'exécution des tâches informatisées. Par ailleurs, cette étude a effectué une évaluation de suivi quatre semaines après la mesure post-intervention, qui a montré une amélioration subséquente des tâches mesurées, comparé aux résultats de la mesure post-intervention. Les participants n'avaient pas poursuivi les exercices du programme d'entraînement entre la fin de l'intervention et le suivi.

Patients MP-TCL. Les études plus récentes sur l'entraînement cognitif dans la MP utilisent les critères de Litvan et al. (2012) pour inclure des patients MP-TCL. Généralement, aucune distinction n'a été faite entre les TCL amnésiques ou non amnésiques: tous les sous-types de TCL étaient inclus. Évidemment, cela peut directement influencer les résultats suite à l'entraînement, puisque plusieurs programmes ciblaient des fonctions cognitives spécifiques. Ainsi, il est possible que certains programmes entraînaient des fonctions qui étaient intactes chez des participants, ce qui pourrait amener un effet de plafond, sans vraiment aider le patient. D'abord, Angelucci et ses collaborateurs (2015) se sont intéressé aux effets physiologiques d'un entraînement cognitif chez des patients MP-TCL. En plus de mesurer les effets sur la cognition via le «*Zoo Map Test*», les auteurs ont aussi mesuré les taux du «*Brain Derived Neurotrophic Factor*» (BDNF) dans le sang, un indicateur de croissance neuronale. L'échantillon de recherche comprenait 15 participants : sept dans le groupe entraîné et huit dans un groupe «contrôle actif» (exercices non liés aux fonctions exécutives, par exemple des dictées). L'entraînement cognitif consistait en une série d'exercices ciblant les habiletés d'alternance et de résolution de problème. Pour les deux groupes, des rencontres de 45 minutes ont eu lieu trois fois par semaine, durant quatre semaines. Leurs résultats montrent une amélioration significative à la performance du «*Zoo Map Test*» ainsi qu'une augmentation des taux de BDNF sanguins pour le groupe expérimental comparé au groupe contrôle, bien qu'aucune corrélation entre ces deux variables n'ait pu être établie. Les auteurs concluent quand même que le BDNF sanguin constituerait un bon biomarqueur de l'amélioration cognitive suite à un programme d'entraînement (Angelucci et al., 2015). Aucune donnée de suivi après l'évaluation post-intervention immédiate n'a été mesurée.

L'étude de Cerasa et ses collaborateurs (2014) a tenté de vérifier si un programme d'entraînement cognitif apportait des changements sur des mesures d'IRMf. Leur échantillon était composé de 15 patients MP-TCL : huit participants dans le groupe entraîné et sept participants dans un groupe contrôle actif, qui complétaient un programme informatique à la maison. Pour être inclus dans l'étude, les patients devaient présenter au moins un déficit cognitif en attention, en vitesse de traitement de l'information ou en fonctions exécutives, mais les sous-types de TCL n'ont pas été décrits plus en détails. L'entraînement cognitif se faisait sous forme de deux rencontres d'une heure par semaine, durant six semaines. Il s'agissait d'activités informatisées axées sur l'attention et le traitement de l'information, ce qui correspondait au profil cognitif des participants et donc, ces activités ciblaient leurs besoins. Les activités du groupe contrôle actif consistaient en des séances de tâches informatiques axées sur la coordination visuo-motrice et étaient de la même durée que les activités du programme d'entraînement ciblé sur l'attention et la vitesse de traitement de l'information. Avant et après l'entraînement, une batterie d'évaluation neuropsychologique complète a été complétée en plus d'une mesure d'IRMf en condition de repos. Les résultats montrent que la condition d'entraînement améliorait la performance aux sous-tests Empan endroit et Codes de la WAIS-IV seulement pour le groupe expérimental. De plus, une hausse d'activité fonctionnelle a été observée au niveau du cortex pariétal supérieur et du cortex préfrontal dorsolatéral chez les patients du groupe expérimental, associés selon les auteurs à l'attention et au contrôle exécutif, respectivement. Leur conclusion est donc qu'un programme d'entraînement cognitif favoriserait la plasticité cérébrale (Cerasa et al., 2014).

Une autre étude publiée (Costa et al., 2014) a comparé un entraînement cognitif axé sur les habiletés d'alternance (fonctions exécutives) avec un entraînement non spécifique axé sur les habiletés langagières. L'échantillon était composé de 17 patients MP-TCL : neuf participants complétaient le programme cognitif et huit complétaient le programme langagier. Les mesures neuropsychologiques pré et post-intervention étaient le TMT et le test de fluidité verbale, condition d'alternance, de la batterie «*Delis et Kaplan Executive Function System*» (D-KEFS). Les résultats montrent que le groupe entraîné sur les fonctions exécutives améliorait significativement sa performance à ces deux mesures suite à l'entraînement, ce qui n'était pas le cas du groupe contrôle entraîné avec des exercices de langage.

Dans la méta-analyse de Hindle et al. (2013) et celle de Leung et al. (2015), une seule étude avait une qualité méthodologique supérieure : l'étude de Paris et al. (2011). Il s'agit d'une étude avec devis randomisé – contrôle en double-aveugle ayant pour but de tester un programme d'entraînement cognitif. Ce dernier est basé sur des tâches informatisées et des tâches papier-crayon, à raison de trois séances de 45 minutes par semaine, pendant quatre semaines. L'échantillon de l'étude comprenait 28 participants séparés en deux groupes : un groupe entraîné (n = 16) et un groupe contrôle actif (n = 12). Les patients inclus dans l'étude ont été évalués avec une batterie neuropsychologique exhaustive permettant de caractériser le TCL, a posteriori (avant la publication des critères de Litvan et al., 2012). Le groupe contrôle a suivi des séances de thérapie du langage en groupe de la même durée que l'intervention du groupe expérimental. Les participants étaient majoritairement des patients atteints de la MP sans déficit cognitif significatif. Toutefois, huit participants dans le groupe entraîné et six dans le groupe contrôle remplissaient les critères du TCL selon les résultats de l'évaluation neuropsychologique complète. Les résultats montrent une amélioration significative suite à l'entraînement aux mesures d'attention (séquences de chiffres de la WAIS-III), de vitesse de traitement de l'information (Codes de la WAIS-III; Stroop mots; TMT-A), de mémoire (CVLT), d'habiletés visuo-spatiales et visuo-constructives (figure complexe de Rey), de fluidité sémantique (production de noms d'animaux) et de fonctions exécutives (Tour de Londres; Stroop interférence; TMT-B). Il est à noter que l'étude ne mentionne pas l'utilisation de versions alternatives des tests, il se pourrait donc qu'un effet de pratique explique en partie les résultats. De plus, aucune amélioration n'a été notée pour ce qui est de la qualité de vie et de l'accomplissement des AIVQ. Aucune évaluation de suivi n'a été faite suite à la mesure post-entraînement.

<u>Limites des interventions cognitives</u>

En résumé, chez des patients MP, les études d'intervention cognitive semblent prometteuses, mais plusieurs limites méthodologiques empêchent la généralisation des résultats (Hindle et al., 2013). D'abord, aucun des programmes n'était particulièrement adapté pour la population atteinte de MP : pour la plupart, les programmes étaient informatisés et pouvaient être employés avec d'autres problématiques, comme les patients atteints de TDAH ou de la MA. Ainsi, les programmes d'entraînement ne semblent pas spécifiques pour des patients dont le profil cognitif est particulièrement hétérogène. Un programme qui prend en compte et cible les différentes atteintes des patients serait probablement plus efficace. À cet

effet, il est important de rappeler que les études incluant des patients MP-TCL n'avaient, pour la plupart, pas caractérisé le type de TCL chez leurs participants, mais des programmes spécifiques à certaines fonctions cognitives étaient pourtant utilisés; par conséquent, il est possible que des fonctions intactes aient été entraînées alors que des fonctions déficitaires ont pu être ignorées. Cela pourrait donc expliquer en partie les résultats modestes qui ont été présentés précédemment.

Également, seulement deux études ont réalisé une évaluation de suivi de plusieurs semaines après la mesure post-intervention des participants : une étude dont le suivi était d'environ 4 semaines après la fin de l'entraînement (Milman et al., 2014) et l'autre, d'une année après (Petrelli et al., 2015). Il n'y a donc que très peu de données sur un suivi à long terme, bien que les résultats semblent prometteurs à l'heure actuelle. Une évaluation de suivi de plusieurs semaines après la fin de l'intervention permettrait de vérifier si les acquis se maintiennent après la fin de l'entraînement et si un déclin cognitif peut être prévenu ou ralenti.

De plus, le but principal de ces études était surtout d'améliorer les fonctions cognitives des patients telles que mesurées par des tests cognitifs dits de «laboratoire». Il n'était donc pas montré que les différents programmes aidaient réellement les patients à améliorer la réalisation des tâches quotidiennes, comme les AIVQ. En fait, aucune des études n'a évalué systématiquement la réalisation des AIVQ chez les participants.

Toujours en lien avec les tâches neuropsychologiques, peu d'études ont mentionné si elles utilisaient les mêmes mesures en pré- et post-intervention, ce qui pourrait occasionner un effet de pratique chez les participants, surtout étant donné le court intervalle de temps écoulé entre les évaluations pré- et post-intervention (en moyenne, quatre semaines). La littérature rapporte incidemment qu'un effet de pratique peut survenir si les mêmes tests sont ré-administrés à six mois et moins d'intervalle (McCaffrey et al., 1993). Très peu d'études d'entraînement cognitif ont rapporté l'utilisation de versions parallèles des tests neuropsychologiques, ce qui aurait pu diminuer un éventuel effet de pratique (Angelucci et al., 2014).

Concernant les autres mesures importantes à inclure, les symptômes psychologiques et comportementaux (SPC) peuvent avoir une incidence sur l'efficacité de l'intervention cognitive. Par exemple, la difficulté à accomplir des tâches pourrait générer de la détresse supplémentaire chez certains patients. Il serait donc important de documenter des effets sur les SPC, de la même manière que

le sont les effets secondaires d'un traitement pharmacologique. À l'inverse, l'accomplissement d'un programme d'entraînement pourrait améliorer les SPC si le patient se sent plus efficace au quotidien. Avec les données actuellement disponibles, il n'est pas possible de confirmer l'impact d'un entraînement cognitif sur les SPC. Dans les études présentées ci-haut, la plupart des patients ne présentaient que très peu de symptômes dépressifs au départ, puisque la présence d'un diagnostic psychiatrique était fréquemment un critère d'exclusion. Ainsi, une amélioration serait plus difficile à trouver étant donné un effet plafond. Une majorité des études n'incluaient qu'une mesure de dépression, et non une évaluation exhaustive des SPC. Ceci dit, aucune des études n'a rapporté de détérioration au niveau des SPC, même si les symptômes dépressifs étaient plus souvent évalués. Concernant les SPC autres que la dépression, justement, très peu de données sont détaillées dans les études. Par exemple, deux études ont évalué l'anxiété, mais aucun des programmes n'a permis d'améliorer ces symptômes (Cerasa et al., 2014; Pena et al., 2014). De plus, aucune étude n'a mesuré le fardeau de l'aidant naturel, alors que les patients MP ont souvent besoin d'une aide au quotidien. Avec un programme d'entraînement informatisé, peu ou pas adapté aux difficultés cognitives des patients, il serait surprenant d'obtenir un changement pour le patient ou son aidant. Avec un programme mieux adapté, il serait plus probable d'observer une amélioration du sentiment de fardeau de l'aidant.

En ce qui concerne le contenu des programmes, une majorité des études porte sur des programmes d'entraînement cognitif, qui manquent de transférabilité sur les activités de la vie quotidienne pour la plupart. En effet, seules les activités accomplies lors de l'entraînement semblent améliorées, sans que les activités du quotidien ne soient particulièrement documentées. Bien que plusieurs programmes aient ciblé les fonctions exécutives, très peu de détails étaient donnés quant aux fonctions exécutives précisément entraînées, par quels mécanismes, avec quelles techniques cognitives ces fonctions étaient entraînées, et comment le niveau de difficulté était ajusté en fonction des performances de chaque participant au fur et à mesure que l'entraînement évoluait. Étant donné que les fonctions exécutives sont souvent atteintes dans le TCL et qu'elles forment souvent l'objet principal des plaintes cognitives des patients (Sollinger et al., 2010; Tröster, 2011), il serait judicieux de cibler particulièrement une amélioration de ces fonctions pour aider les patients à mieux fonctionner dans leur quotidien (Dirnberger et Jahanshahi, 2013). Une évaluation de base rigoureuse faite auprès des patients afin de bien

caractériser le TCL prend donc toute son importance, afin de vraiment ajuster le programme en fonction de leurs besoins. Des programmes d'entraînement pourraient être ainsi mieux adaptés aux patients MP-TCL.

Le Goal Management Training

Contrairement aux programmes d'entraînement mentionnés ci-haut, le *Goal Management Training* (GMT) est à la croisée entre l'entraînement cognitif et la réadaptation cognitive. Il s'agit d'un programme ciblant spécifiquement les fonctions exécutives et adapté aux besoins du patient, bien qu'il puisse être accompli en petits groupes (Levine, Robertson, Clare, Carter, Hong, Wilson et al., 2000). En effet, tout au long du programme, le patient est amené à prendre conscience de ses difficultés et à mettre au point des solutions concrètes avec l'aide de l'intervenant. Un de ses avantages majeurs est que le GMT est basé sur un modèle théorique bien connu des fonctions exécutives, la théorie de Duncan sur la désorganisation du comportement suite à une lésion ou un dysfonctionnement du lobe frontal (Duncan, 1986; Robertson, 1996).

Le but du GMT est d'aider le patient à prendre conscience de ce qu'il fait dans le moment présent afin de l'aider à accomplir efficacement les AIVQ (Robertson, 1996; Levine et al., 2000). Dans sa forme originale, cinq étapes sont apprises au participant, comme une routine, pour l'aider à structurer ses comportements : 1) apprendre à s'arrêter pour vérifier ce qu'il est en train de faire grâce à des techniques de pleine conscience; 2) formuler le but principal de son action en cours; 3) diviser les tâches en sous-objectifs; 4) apprendre les sous-objectifs et les encoder; 5) comparer les actions entreprises avec les buts fixés et vérifier s'il y a concordance. Le GMT se fait sous forme de rencontres individualisées ou en petits groupes. Le programme original de 5 étapes est divisé en neuf modules qui prennent environ 90 à 120 minutes chacun à administrer. Il est conseillé par les auteurs de faire un module par semaine. Après chaque module, les participants doivent compléter quelques exercices à la maison, tel que d'écrire dans un tableau les erreurs commises et les réussites vécues au cours de la dernière semaine. D'autres exercices, tel que la technique du balayage corporel, sont directement inspirés du mouvement de la pleine conscience et permettent au participant de se centrer dans le moment présent.

Plusieurs études ont montré que le GMT était efficace et pouvait être appliqué à des situations concrètes vécues par les patients (Levine et al., 2000; Levine, Stuss, Winocur, Binns, Fahy, Mandic, et

al., 2007; Levine, Schweizer, O'Connor, Turner, Gillingham, Stuss et al., 2011; Schweizer, Levine, Rewilak, O'Connor, Turner, Alexander et al., 2008; van Hooren, Valentijn et Bosma, 2007). Plusieurs études de cas ont été menées chez des patients avec traumatismes crâniaux cérébraux (Levine et al., 2000), des lésions cérébelleuses (Schweizer et al., 2008) ou avec la maladie du *spina bifida* (Stubberud, Langenbahn, Levine, Stanghelle, et Schanke, 2013). Toutes ces pathologies, comme la MP, incluent des déficits affectant le fonctionnement du lobe frontal, essentiellement la structure qui sous-tend la réalisation des fonctions exécutives. Quelques études avec devis randomisé – contrôle ont aussi été menées et ont montré des résultats intéressants.

Une étude à devis randomisé – contrôle a été menée en 2007 auprès de 49 patients âgés sains, mais qui avaient des plaintes cognitives. Il est intéressant de noter que les patients âgés, même sans atteintes cognitives objectives, présentent eux aussi une dégénérescence du lobe frontal, bien qu'elle soit beaucoup moins marquée que chez des populations pathologiques (Levine et al., 2007). Après la fin du GMT, les patients s'étaient améliorés sur plusieurs tâches écologiques mesurant les fonctions exécutives, les «*Simulated Real Life Tasks*». De plus, leur compétence auto-rapportée pour ces tâches, mesurées par des questionnaires, était également améliorée, ce qui montre que les patients se sentaient mieux outillés pour gérer leurs activités quotidiennes (Levine et al., 2007). Les améliorations se sont maintenues trois mois après la fin de l'intervention.

Ces résultats ont été répliqués dans un autre échantillon de 69 personnes âgées ayant des plaintes cognitives (van Hoorren et al., 2007). Le groupe GMT comprenait 38 participants, et le groupe contrôle passif de type liste d'attente en comprenait 31. Dans cette étude, les mesures d'efficacité incluaient principalement un questionnaire auto-rapporté sur les troubles cognitifs perçus, le «*Dysexecutive* (DEX) *questionnaire*», les sous-échelles Dépression et Anxiété de la «*Symptom Checklist – 90 items*», ainsi que la performance au test de Stroop. Les résultats montrent que les participants ayant complété le GMT percevaient qu'ils avaient moins de problèmes cognitifs et qu'ils présentaient moins de symptômes anxieux et dépressifs. Par contre, il n'y avait aucune différence significative entre la mesure pré-GMT et la mesure post-GMT sur les performances au test de Stroop. Toutefois, le Stroop mesure les capacités d'inhibition, et n'est donc pas une tâche d'organisation. Par conséquent, une amélioration à cette tâche

aurait été improbable, puisque le GMT cible principalement l'amélioration des stratégies d'organisation et de planification.

Une autre étude a été menée auprès de 19 patients atteints de lésions frontales suite à un accident vasculaire cérébral. Cette fois-ci, deux tâches cognitives étaient améliorées suite au GMT : la «*Sustained Attention to Response Task*», une tâche de «Go – No Go», et le «*Tower Test*», décrit dans l'étude comme une tâche de construction visuospatiale complexe, mais associée fréquemment à une mesure des fonctions exécutives (e.g. planification). Par contre, aucune amélioration n'a été observée pour ce qui est de l'évaluation auto-rapportée. Toutefois, les auteurs notent que leur échantillon était très hétérogène pour ce qui est des atteintes frontales, expliquant la grande variabilité des résultats tels qu'évalués par les mesures auto-rapportées (Levine et al., 2011).

En résumé, le GMT est bien adapté et validé auprès de différentes populations ayant des déficits exécutifs causés par des altérations frontales. Il semble toutefois important de noter certaines limites du GMT. D'abord, le programme est disponible en anglais uniquement et n'a pas été traduit officiellement pour la population francophone. Aussi, il s'agit d'un programme chargé, car il inclut de nombreuses discussions et exercices qui demandent une grande attention de la part du participant. Cela pourrait donc être lourd pour des patients présentant une fatigabilité au quotidien. Entre autres, les exercices qui doivent être complétés à la maison pourraient être perçus comme trop exigeants en temps et en effort demandé par certains patients. De plus, ce programme n'a pas été conçu pour des personnes présentant des difficultés motrices comme celles associées à la MP. Par exemple, de nombreux tableaux doivent être remplis à la main et quelques exercices demandent de distribuer des cartes à jouer, ce qui pourrait occasionner une fatigue motrice supplémentaire. Pourtant, chez certains patients MP-TCL, les déficits exécutifs sont explicables par des altérations au niveau frontal, régions interconnectées étroitement aux ganglions de la base, et dont le fonctionnement est affecté sévèrement dès le début de la MP. Une intervention comme le GMT pourrait donc bien être adaptée aux patients MP aux prises avec des problèmes exécutifs et pourrait éventuellement les aider à mieux fonctionner dans les activités de la vie quotidienne.

Objectifs généraux et hypothèses du projet

Les objectifs généraux du présent projet sont de traduire en français le manuel d'administration et le matériel du GMT, de l'adapter à la population parkinsonienne, de vérifier ensuite sa tolérabilité (fatigue, SPC, changements de médication ou de dosage et fardeau de l'aidant) et son efficacité chez des patients atteints de MP présentant un TCL. Deux grandes étapes serviront à réaliser ces objectifs: (1) une étude de cas à niveaux de base multiples pour les trois premiers objectifs et (2) une étude avec devis randomisé – contrôle pour valider le dernier. Pour l'étude randomisée – contrôle, l'efficacité du programme sera évaluée sur les fonctions exécutives et les AIVQ. Les objectifs secondaires concerneront l'évaluation des SPC, de la qualité de vie des patients MP, de la cognition globale ainsi que du fardeau de l'aidant. Un dernier objectif sera d'explorer la relation entre le fonctionnement de la théorie de l'esprit avant le début du GMT et l'amélioration des fonctions exécutives et des AIVQ après l'intervention.

Les hypothèses générales sont que le GMT **1)** améliorera les fonctions exécutives telles que mesurées par des questionnaires auto- et hétéro-rapportés ainsi que par une tâche écologique qui seront décrits dans les prochaines sections. De plus, puisque le GMT est plus concret qu'un programme d'entraînement cognitif, deux objectifs plus exploratoires sont de voir si le GMT permet **2)** d'améliorer ou de maintenir les SPC tels que mesurés par le «*Neuropsychiatric Inventory*» (NPI), la cognition globale telle que mesurée avec la «*Dementia Rating Scale – 2*» (DRS-2), ainsi que l'accomplissement des AIVQ et la qualité de vie tels que mesurés par le «*Parkinson Disease Questionnaire – 39 items*» (PDQ-39). En ce qui concerne le fardeau de l'aidant naturel, il pourrait y avoir **3)** soit une amélioration au niveau du fardeau, soit aucun changement, tel que mesuré par le «*Zarit Burden Interview – 12 items*» (ZBI-12). Enfin, la dernière hypothèse est que **4)** la capacité de théorie de l'esprit des patients MP-TCL, telle que mesurée par la tâche de Faux Pas à l'évaluation de base, sera un prédicteur de l'amélioration des fonctions exécutives post-GMT.

Étude 1 : méthodologie

Objectifs et hypothèses

Les objectifs de la première étude sont de **1)** traduire le matériel d'administration et le contenu du GMT, **2)** de l'adapter spécifiquement aux patients MP, **3)** de vérifier si le GMT est bien toléré et sécuritaire pour des patients MP-TCL et **4)** de vérifier si les modifications sont efficaces pour la

population ciblée. Le dernier objectif sera **5)** d'explorer la relation entre la capacité de théorie de l'esprit et la réussite du GMT.

Les hypothèses sont donc que **1)** le GMT sera bien toléré, donc que les participants ne se décriront pas comme trop fatigués (selon une évaluation auto-rapportée menée à la fin de chaque séance), qu'il n'y aura pas de changement dans leurs médicaments (antiparkinsoniens, antidépresseurs, ou autre médication psychotrope), qu'il y aura une réduction ou un maintien des SPC tels que rapportés par un proche aidant, ainsi qu'une amélioration ou un maintien du sentiment de fardeau de l'aidant. **2)** Le GMT modifié permettra aux participants d'améliorer leurs fonctions exécutives, la réalisation des AIVQ, leur qualité de vie ainsi que d'améliorer ou de maintenir leur cognition globale. Enfin, **3)** la capacité de théorie de l'esprit, telle que mesurée lors de l'évaluation du niveau de base, constituera un bon prédicteur de l'efficacité du GMT.

Devis expérimental

Une étude de cas à niveaux de base multiples sera le devis utilisé. Le principe est que chaque participant doit obtenir des résultats stables sur les mesures de l'évaluation de base *avant* de commencer l'intervention (Levine et Downey-Lamb, 2005). Un premier participant débute l'intervention alors qu'un second participant poursuit les évaluations de base; puis éventuellement, le second participant reçoit également l'intervention. Ainsi, il est possible de mieux contrôler les différences individuelles existant entre les participants et d'améliorer la validité interne de l'étude en montrant qu'un changement pré – post dans les mesures peut être vraisemblablement mieux expliqué par l'intervention que par des éléments externes à l'intervention (Hawkins, Sanson-Fisher, Shakeshaft, D'Este, et Green, 2007; Levine et Downey-Lamb, 2005).

Recrutement

Puisqu'une première étude qui a eu lieu dans le laboratoire portait sur le profil cognitif des patients MP, l'étudiante au Ph.D. regardera les dossiers des participants de cette première étude qui ont signé une clause du formulaire de consentement afin d'être recontactés pour des recherches ultérieures (numéro d'approbation PEJ-703, Maxime Doiron). S'il manque des participants, les autres seront recrutés à la clinique neurologique des troubles du mouvement (Département des Sciences Neurologiques, CHU – pavillon Enfant-Jésus) par Dr Nicolas Dupré, neurologue. Ce dernier, lors de consultations cliniques,

identifiera les personnes qui pourraient répondre aux critères d'inclusion et leur proposera de faire partie du projet de recherche, notamment en leur remettant un feuillet d'information sur l'étude. Il leur demandera leur consentement afin que leurs coordonnées soient transférées à la candidate au Ph.D en respectant les règles de confidentialité. Un autre moyen de recrutement sera de passer par l'organisme Parkinson Québec. Cet organisme tient des conférences et des groupes de soutien destinés aux personnes atteintes de MP des régions de Québec et de Chaudière-Appalaches, durant lesquels l'étudiante au Ph.D présentera son projet de recherche en recueillant les coordonnées des personnes intéressées. Ensuite, peu importe le moyen de recrutement, l'étudiante en charge du projet communiquera par téléphone avec les éventuels participants afin de leur expliquer plus précisément en quoi consistent les objectifs de l'étude et l'intervention. Si la personne accepte de participer, une première rencontre pour la signature des formulaires de consentement et le début de l'évaluation diagnostique sera fixée.

Participants

Deux participants seront recrutés pour l'étude de cas selon les critères suivants.

Critères d'inclusion

1. Diagnostic de la MP selon la *UK PD Brain Bank* (Hughes et al., 1992).
2. Diagnostic de MP-TCL, type amnésique à domaines multiples ou non-amnésique à domaine simple ou à domaines multiples, mais toujours avec présence de dysfonctions exécutives (selon une évaluation de type 2 de Litvan et al., 2012).
   - Déclin graduel des habiletés cognitives, tel qu'observé par le patient, son proche aidant et/ou par le clinicien.
   - Déclin cognitif objectif, tel que mesuré par des résultats entre moins un et moins deux écart-types (mais pas en-deça de moins deux écart-types) de la moyenne (normes selon l'âge et/ou le niveau d'éducation) à au moins un test des fonctions exécutives. Il pourra y avoir une performance entre moins un et moins deux écart-types par rapport à la moyenne sur d'autres tests mesurant les fonctions exécutives et/ou dans un autre domaine cognitif parmi : 1) attention et mémoire de travail, 2) fonctions langagières, 3) mémoire épisodique ou sémantique, 4) fonctions visuo-spatiales. Deux tâches neuropsychologiques par domaine

devront être effectuées pour confirmer l'altération cognitive objective du participant (critère de l'évaluation neuropsychologique de type II, Litvan et al., 2012).

- Les difficultés n'interfèrent pas significativement avec les activités quotidiennes de base, mais le patient pourra éprouver certaines difficultés dans les AIVQ dans le cadre de la présente étude.
- Les cas de démence parkinsonnienne légère, avec un score au MoCA entre 21 et 26 tels qu'établis par les études de Dalyrimple-Alford et al. (2010) et de Robben et al. (2010), seront acceptés advenant le cas où il n'y aurait pas suffisamment de patients MP-TCL.

3. Présence d'un proche aidant prêt à participer aux activités avec le patient et à remplir les questionnaires.
4. Médication antiparkinsonienne (L-DOPA ou autre) stable depuis au moins 2 mois.
5. Médication psychotrope et/ou nootrope stable depuis au moins 3 mois.

Critères d'exclusion

1. Diagnostic de démence parkinsonienne ou un score au MoCA en deçà de 21 (Dalyrimple-Alford et al., 2010; Robben et al., 2010).
2. Diagnostic d'un autre trouble neurologique pouvant causer des troubles cognitifs.
3. Histoire ou présence d'abus de substance.

Procédure

Toutes les rencontres auront lieu au domicile du participant, en compagnie du proche aidant. Ces rencontres auront lieu lorsque la médication du patient sera optimale en termes de contrôle des symptômes moteurs. Afin de déterminer la période ON, les patients et leur proche seront interrogés par l'étudiante au doctorat et les rencontres seront fixées en conséquence. Lors de la première rencontre, toutes les modalités de l'étude seront présentées en détails et les formulaires d'information et de consentement seront signés par le participant et son proche. La doctorante demeurera disponible pour répondre à toutes les questions des participants ou de leur proche. Suite à l'obtention du consentement, l'évaluation diagnostique pourra débuter. Pour ne pas fatiguer le participant, au moins trois rencontres d'évaluation diagnostique seront prévues, d'une durée maximale chacune de 60 à 75 minutes. Le tableau

6.1 de l'annexe 6 fournit une estimation des temps requis pour chacune des mesures de la batterie diagnostique, d'une durée totale maximum d'environ trois heures.

Lorsque l'évaluation diagnostique aura permis de confirmer que le participant rencontre bien les critères d'inclusion de l'étude, l'administration des mesures de niveau de base pourra commencer. L'évaluation du participant sera administrée par la candidate au Ph.D lors de rencontres fixées d'avance. Pour ce qui est des questionnaires au proche aidant, ils seront administrés par l'étudiante à un autre moment, lors d'un rendez-vous téléphonique, par souci de discrétion. Au moins deux évaluations de niveau de base seront faites pour le premier participant, alors qu'au moins trois évaluations du niveau de base seront faites pour le deuxième participant. Cette façon de faire suit les procédures standards du devis à niveaux de base multiples (Levine et Downey-Lamb, 2005). Dans le cas où les mesures ne soient pas suffisamment stables après le nombre de rencontres préétablies, d'autres évaluations de niveau de base seront ajoutées au besoin.

Lorsque les résultats aux mesures du niveau de base seront stables, l'intervention pourra débuter. Celle-ci aura également lieu au domicile du participant, à raison d'une fois par semaine, durant cinq semaines, en présence de l'aidant naturel. Un module par semaine sera couvert, et les participants auront des activités à compléter entre les séances. La durée de chaque séance pourra être variable (en moyenne 60 minutes). Dans le cas où le patient serait trop fatigué, les différentes séances du GMT peuvent être séparées en deux séances plus courtes au besoin. Une semaine et un mois après la fin du GMT, des mesures post-intervention seront administrées selon les mêmes modalités que pour l'évaluation du niveau de base.

Intervention

Le GMT original est constitué de neuf modules administrés à raison d'une session d'une heure et demie par semaine, et qui ont pour objectif d'apprendre les étapes d'une stratégie pour rester conscient de ce que le patient est en train de faire, sans se détourner d'un objectif final (Levine et al., 2000; 2007). Le patient apprend notamment les étapes d'un processus dans lequel il doit prendre le temps de S'ARRÊTER, de FORMULER son objectif principal et de le DIVISER en sous-tâches autant de fois que nécessaire. Toutefois, pour des raisons pratiques, certains ajustements du GMT seront effectués pour qu'il soit plus tolérable pour des patients MP présentant des difficultés motrices et une grande fatigabilité.

Par exemple, alors que le GMT original comprend de nombreux exercices de distribution de cartes à jouer (exercices d’alternance de règle), le GMT modifié n’inclut pas ces exercices. Il en va de même pour quelques-uns des tableaux à remplir à la main dans le manuel du participant, dont les thèmes seront plutôt abordés sous forme de discussion. Le programme original a été quelque peu raccourci pour être complété en cinq semaines au lieu de neuf, et chaque séance est d’une durée maximum de 60 à 75 minutes, au lieu de 90 à 120. Toutefois, le GMT ainsi modifié devra toujours permettre aux patients d’apprendre de nouvelles stratégies pour pallier à leurs déficits (Levine et al., 2000; 2007; 2011; Schweizer et al., 2008; Stubberud et al., 2013; van Hoorren et al., 2007).

Mesures

Plusieurs tâches neuropsychologiques standardisées et validées seront effectuées pour l’évaluation diagnostique afin de vérifier les critères d’inclusion. En ce qui concerne les mesures de niveau de base et de suivi, des questionnaires seront utilisés afin de vérifier si le GMT permet une amélioration des fonctions exécutives et des autres concepts évalués. Il y aura également une mesure neuropsychologique spécifique aux fonctions exécutives, possédant une forme parallèle, et dont les deux versions (originale et parallèle) seront administrées en alternance, afin de minimiser l’effet de pratique. Une autre étudiante du laboratoire, Laïla El-Amrani, fera l’évaluation post-GMT, pour que les résultats finaux demeurent en double aveugle. L’utilisation de questionnaires auto-rapportés donne un aperçu des déficits tels qu’ils sont perçus par le participant, tandis que les questionnaires remplis par le proche aidant pourront compléter et valider les informations obtenues. Les tableaux 6.1, 6.2 et 6.3 de l’annexe 6 fournissent une estimation des temps requis pour chacune des mesures de la batterie diagnostique et des mesures d’efficacité du GMT.

Batterie de tests pour l’évaluation diagnostique

La batterie de tests qui sera utilisée pour l’évaluation diagnostique des participants est décrite en détails dans l’annexe 4. Les tests ont été choisis de façon à remplir les critères d’évaluation de type II du MP-TCL selon Litvan et al. (2012).

Questionnaires et tests pour mesurer la tolérabilité, la sécurité et l’efficacité du GMT

Les mesures primaires choisies seront les mesures de tolérabilité, de sécurité, des SPC et du fardeau de l'aidant. Les mesures secondaires concerneront les fonctions exécutives, la qualité de vie, les AIVQ et la cognition globale.

Tolérabilité et sécurité. L'assiduité aux séances et aux activités proposées, le niveau de fatigue ressentie à chaque séance (échelle Likert de 0-10, voir l'annexe 5) et les changements de médication ou de dosage de la médication seront utilisés comme mesures de la tolérabilité. De plus, les mesures de SPC et de fardeau de l'aidant, qui seront décrites dans une prochaine section, serviront également à mesurer la tolérabilité et la sécurité du GMT, dans le cadre de l'étude pilote.

Fonctions exécutives. Les fonctions exécutives seront évaluées à l'aide de mesures auto-rapportées par les participants et également par une mesure cognitive objective.

Le «*Dysexecutive* (DEX) *questionnaire*» (Wilson et al., 1996), inclus dans la BADS, contient 20 questions. Il est auto-rapporté et concerne des comportements de désorganisation qui sont cotés sur une échelle de 0 à 4 (0 correspondant à «jamais» et 4 à «presque toujours») pour un score maximum négatif possible de 80 (plus le score est élevé, plus la personne vit des problèmes). Il possède une bonne validité de convergence et corrèle positivement avec le score total de la BADS. Il est considéré comme une mesure écologique, bien adaptée pour mesurer les déficits exécutifs vécus par les patients frontaux (Wilson et al., 1996).

Le questionnaire fourni dans la trousse du GMT (traduit par l'étudiante au Ph.D.) comprend 34 questions sur des erreurs commises fréquemment qui doivent être cotées sur une échelle de 0 à 10 (0 correspondant à «aucun problème» et 10 correspondant à un «problème très grave»). Le score maximum est donc de 340 (plus le score est élevé, plus la personne vit des problèmes).

Une mesure de la BADS a été choisie comme mesure objective des fonctions exécutives, puisqu'elle est disponible dans une version originale et une version parallèle, ce qui permettra de prendre des mesures pré-intervention et post-intervention en évitant le plus possible un effet de pratique. En effet, le «*Zoo Map Test*» mesure les capacités d'organisation et de planification. Le participant doit établir l'itinéraire qu'il devrait suivre dans un zoo. Il peut être guidé ou non par l'intervenant. Le «*Zoo Map Test*» possède une bonne validité de construit et une bonne corrélation avec des tâches classiques mesurant les fonctions exécutives (Wilson et al., 1996).

Cognition globale. La «*Dementia Rating Scale 2*» (DRS-2; Mattis, 2001) est une mesure comprenant une forme originale et une forme alternative, ce qui fait qu'elle pourra être administrée à deux reprises aux participants, avant et après le GMT. Brièvement, elle se divise en cinq sous-échelles : attention, initiation / persévération, construction, concepts et mémoire; son score total maximum possible (positif) est de 144. De plus, il existe maintenant des normes québécoises pour la coter (Lavoie et al., 2013). Cet outil est considéré comme ayant de bonnes propriétés psychométriques et il permet de détecter efficacement le déclin cognitif chez les patients MP-TCL et ceux atteints de démence parkinsonienne (Matteau et al., 2011; 2012).

Activités instrumentales de la vie quotidienne et qualité de vie. Le PDQ-39 est un questionnaire spécifique à la population atteinte de MP et en 39 items. Il évalue huit dimensions reliées à la qualité de vie : l'incapacité motrice (10 items), les AVQ (6 items), le bien-être émotionnel (6 items), la stigmatisation (4 items), le support émotionnel (3 items), la cognition (4 items), la communication (3 items) et le confort (3 items) (Pirogovsky et al., 2014; Rosenthal et al., 2010). Chaque item est coté sur une échelle allant de «jamais» (0 point) à «toujours» (4 points) et tous les scores (pour chaque échelle et score total) sont changés en pourcentage. Plus le score est élevé, moins bonne est la qualité de vie. Ce questionnaire est largement utilisé en raison de sa bonne validité de construit, sa fidélité test-retest ainsi que sa bonne consistance interne (Peto et al., 1998).

Fonctions exécutives du participant, cotées par un proche. Le DEX (version aidant) ainsi que le questionnaire fourni dans la trousse du GMT (version aidant contenant 25 questions dans les mêmes modalités que le questionnaire auto-rapporté, traduit par l'étudiante au Ph.D) seront administrés afin de vérifier si les changements apportés par le GMT sont perceptibles pour le proche du participant.

Fardeau de l'aidant. Le ZBI-12 est un questionnaire auto-rapporté mesurant le sentiment de fardeau de l'aidant en 12 questions (Bédard et al., 2001; Hébert et al., 2007). La cotation se fait sur une échelle de type Likert en cinq points («jamais» correspond à un score de 0 et «toujours» correspond à un score de 4). Le score total maximum est de 48, ce qui correspond à un sentiment de fardeau élevé. Il s'agit d'une mesure largement utilisée chez les aidants naturels, notamment en raison d'une bonne fidélité test-retest (Hébert et al., 2007).

Symptômes cognitifs et comportementaux. Le NPI-12 (versions aidant et clinicien) permet de mesurer différents symptômes tels que les délires, les hallucinations, l'agitation, la dysphorie, l'anxiété, l'apathie, l'irritabilité, l'euphorie, la désinhibition, les difficultés motrices, les difficultés de sommeil et les comportements alimentaires problématiques (Cummings et al., 1994; De Meideiros et al., 2010). Pour chaque symptôme, la fréquence est cotée sur une échelle de quatre et la sévérité est cotée sur une échelle de trois. Le score pour chaque symptôme est le produit de la fréquence et de la sévérité, ce qui fait un maximum de 12 points par symptôme. Pour le score total, le score de chaque symptôme est additionné, ce qui fait un maximum total de 144 (Cummings et al., 1994; De Meideiros et al., 2010). Les deux versions du NPI ont été validées auprès de la population parkinsonienne et montrent une bonne concordance inter-juges (pour le score total : $r = 0.93$; Aarsland et al., 1999).

Analyses statistiques prévues

Afin de mesurer l'ampleur du changement entre le niveau de base et l'évaluation post-intervention, une mesure de taille d'effet sera utilisée : la «*Common Langage Effect Size*» (CLES), une mesure comparable au *d* de Cohen (McGraw et Wong, 1992) pour chacune des mesures primaires et secondaires. La CLES permet de calculer une taille d'effet spécifique à chaque participant en générant un score Z représentant la différence entre les moyennes du niveau de base (M_1) et les moyennes post-intervention (M_2, McGraw et Wong, 1992).

$$Z_C = (M_1 - M_2) / \sqrt{\ } \ (Var_1 - Var_2)$$

Où Var_1 et Var_2 sont les variances des moyennes pré- et post-intervention.

Modifications suite à l'étude 1 pour la planification de l'étude 2

Les résultats des analyses de l'étude 1 permettront de valider le n nécessaire pour l'étude 2 en complétant une analyse de puissance. La durée de chaque séance a été raccourcie, mais dans le cas où une durée de 60 minutes soit mal tolérée, le format flexible du GMT permettra de scinder chaque séance en deux séances plus courtes, de 30 minutes chacune. De plus, certaines des activités à compléter à la maison pourraient être raccourcies. Toutefois, les études précédentes sur le GMT (Levine et al., 2000; 2007) avaient notamment été menées avec des patients atteints de traumatismes crâniaux cérébraux, populations dont la fatigue cognitive peut être très variable. Il faudra toutefois porter une attention particulière à la fatigue motrice des participants dans le cas de la présente étude.

Étude 2 : méthodologie

Objectifs et hypothèses

Les objectifs de l'étude 2 sont de vérifier si, comparé à une condition contrôle active (programme d'intervention alternatif), le GMT permet d'améliorer **1)** les fonctions exécutives telles que rapportées par le participant et son proche sur le DEX, les questionnaires inclus dans le GMT et le test neuropsychologique écologique évaluant les fonctions exécutives entre les évaluations pré- et post-GMT. De plus, l'étude 2 permettra de vérifier si le GMT amènera: **2)** une amélioration ou un maintien dans l'accomplissement des AIVQ et dans la qualité de vie tels que rapportés par le participant avec le PDQ-39 ainsi que dans la cognition globale telle que mesurée par la DRS-2. Dans le même sens, le GMT pourrait amener : **3)** des changements dans les SPC tel que rapportés par le clinicien et le proche aidant selon le NPI-12; ainsi que **4)** dans le sentiment de fardeau de l'aidant tel que rapporté par le ZBI-12.

Les hypothèses sont que **1)** le GMT sera encore une fois bien toléré, que les fonctions exécutives seront améliorées après l'entraînement. Aussi, **2)** l'accomplissement des AIVQ, la qualité de vie, la cognition globale, les SPC et le fardeau de l'aidant seront soit maintenus, soit améliorés pour le groupe GMT suite à l'entraînement.

Devis expérimental

Un devis randomisé – contrôle en simple aveugle avec groupe contrôle actif sera utilisé pour cette étude. Les participants seront appariés selon l'âge, le sexe, le niveau d'éducation et le type de TCL, puis ils seront assignés aléatoirement à un des deux groupes suivants (n = 5 par groupe, à confirmer) : le groupe 1, expérimental, suivra le GMT durant cinq semaines, à raison d'une séance de 60 à 75 minutes par semaine, et le groupe 2 suivra un programme contrôle (actif), c'est-à-dire des séances de psychoéducation sur la MP et les fonctions cognitives qui seront de même fréquence et de même durée que celles du groupe expérimental.

Recrutement

La même méthode de recrutement que l'étude 1 sera utilisée, à moins d'un changement majeur qui serait apporté au cours de l'étude 1.

Participants

Cette fois-ci, dix à douze participants MP-TCL seront recrutés (n = 5 par groupe; à confirmer). Les critères d'inclusion et d'exclusion seront les mêmes que pour l'étude 1.

Procédure

La procédure pour la rencontre initiale d'information et de consentement sera la même que pour l'étude 1. Pour ce qui est de l'évaluation diagnostique et des mesures de niveau de base, la procédure sera la même que pour l'étude 1, sauf pour ce qui est du nombre d'évaluations de niveau de base (pour l'étude 2, une seule évaluation de niveau de base par participant). Lorsque l'administration des mesures du niveau de base sera complétée, l'intervention pourra débuter. Celle-ci aura également lieu au domicile du participant, à raison d'une fois par semaine, durant cinq semaines, en présence de l'aidant. Un module par semaine sera couvert, et les participants auront des activités à compléter entre les séances. La durée de chaque séance pourra être variable (en moyenne une heure). À la fin du GMT, des mesures post-intervention immédiates seront administrées selon les mêmes modalités que pour l'évaluation du niveau de base. Pour s'assurer d'un devis aveugle, une autre étudiante au Ph.D du laboratoire fera les évaluations post-intervention et de suivi sans connaître auquel des deux groupes chaque participant a été assigné. En ce qui concerne les participants, l'étudiante au Ph.D expliquera au moment de la signature des formulaires de consentement que deux groupes d'entraînement différents seront formés, mais les participants ne seront pas informés du programme auquel ils ont été assignés. Deux rencontres de suivi, quatre et 12 semaines après la mesure post-intervention immédiate, seront effectuées.

Intervention

À moins que des changements ne soient apportés au GMT après l'étude pilote, le même programme que pour l'étude 1 sera administré. En ce qui concerne la condition contrôle actif, il s'agira de séances de psychoéducation sur la MP et les fonctions cognitives atteintes chez les patients, à raison d'une séance de une heure par semaine, durant cinq semaines. Les thèmes de la psychoéducation seront divers : présentation générale de la MP, des atteintes cérébrales et des troubles associés à la MP autres que moteurs. La séance de psychoéducation sera de 30 minutes, et le reste de la rencontre sera consacré à des exercices de pleine conscience (par ex. exercices de respiration et balayage corporel). La psychoéducation constitue une procédure standard chez les patients qui éprouvent des troubles cognitifs (Strauss, Sherman et Spreen, 2006).

Mesures

Les mêmes mesures que l'étude 1 seront utilisées pour l'évaluation diagnostique et l'efficacité du GMT. Cette fois, les mesures primaires choisies seront les mesures d'efficacité sur les fonctions exécutives. Les mesures secondaires concerneront les mesures de tolérabilité, de qualité de vie, de cognition, des SPC, et du fardeau de l'aidant. Pour les évaluations de suivi, qui auront lieu quatre et12 semaines après l'évaluation post-GMT immédiate, les mêmes mesures qui auront été administrées au niveau de base seront utilisées. L'annexe 7 fournit un échéancier pour situer les différentes mesures évaluées au cours des études 1 et 2.

L'échelle d'apathie de Marin (Marin, Biedrzycki et Firinciogullari, 1991) a été ajoutée en mai 2018 au protocole initial. Cette échelle est remplie par le proche aidant et donne un aperçu des symptômes d'apathie. Cette mesure sera administrée au niveau de base, à mi-parcours, ainsi qu'aux suivis post-intervention immédiats, ceux de quatre et 12 semaines.

Analyses statistiques prévues

Pour chaque groupe, pour chaque variable, des statistiques descriptives seront calculées (moyenne, écart-type). Ensuite, un modèle général linéaire mixte, permettant de vérifier l'existence de différences intra- et inter-groupes en ce qui concerne les mesures pré- et post-intervention, sera appliqué pour déterminer si le GMT est supérieur au programme de psychoéducation/exercices de pleine conscience. Le n nécessaire sera déterminé suite à une analyse de puissance menée à partir des résultats de l'étude 1. Toutes les analyses seront menées avec le prologiciel SPSS, version 21.0.

Considérations éthiques

Risques et effets secondaires potentiels

Il se pourrait que certains patients soient anxieux lors de l'évaluation neuropsychologique, car ils voudront bien performer. De plus, les séances d'évaluation neuropsychologique et les séances du GMT pourraient occasionner de la fatigue chez les participants. Étant donné que les séances du GMT se feront une fois par semaine durant cinq semaines, il se pourrait que les participants aient des conflits d'horaire.

L'étudiante a tenté de répondre à ces éventuels problèmes, notamment en rajoutant une question à la fin de chaque module dans le cahier du participant pour s'informer de la fatigue des participants. Si les participants sont trop fatigués pour continuer une séance, le format flexible permettra de séparer les activités en deux ou trois séances, au besoin.

Bénéfices potentiels

Les participants auront accès à une méthode d'entraînement cognitif qui pourrait améliorer leur vie au quotidien et favoriser leur autonomie dans une certaine mesure. Ils en apprendront davantage sur leur fonctionnement cognitif et participeront à l'avancement des connaissances sur la MP et les dysfonctions exécutives qui y sont associées. De plus, advenant le cas où un participant aurait un résultat anormal à une des mesures, il pourra être redirigé vers les ressources appropriées.

Confidentialité

Toutes les données seront confidentielles et seront codées afin que le nom et les coordonnées de chaque participant n'apparaissent sur aucun rapport ou aucune des données informatisées. Les données recueillies au cours de l'évaluation seront conservées dans un dossier gardé sous clé au laboratoire de neuropsychologie gériatrique de l'Université Laval durant 10 ans; elles seront détruites de façon sécuritaire suite à cette période. Seul le code chiffré attribué à chaque participant sera utilisé dans la base de données informatisée (pour les analyses statistiques) et seul ce code apparaîtra dans les fichiers liés aux analyses statistiques. Si la recherche fait l'objet de publications dans des revues et des congrès scientifiques, aucun participant ne sera identifié. Seules l'étudiante au Ph.D et la directrice du laboratoire auront accès au code permettant d'identifier les participants à partir de leur numéro de dossier. Seuls le médecin traitant, l'étudiante au Ph.D et la directrice du laboratoire auront accès aux données nominatives des participants. Les participants pourront signer ou non une clause du formulaire de consentement concernant l'utilisation ultérieure de leurs données dénominalisées pour d'autres projets (analyses secondaires sur banque de données) du laboratoire. S'ils sont intéressés, ils auront accès aux publications concernant leurs données. Toute information permettant de les reconnaître directement ou indirectement sera enlevée par souci de confidentialité.

Consentement libre et éclairé

Les participants sont libres de participer ou non à la recherche et pourront retirer leur consentement en tout temps. Cette procédure est la même pour les proches aidants acceptant de participer. Étant donné que cette recherche s'adresse à des individus majeurs aptes, l'ensemble des procédures scientifiques et éthiques leur sera expliqué en détails afin que leur consentement soit entièrement libre et éclairé. En cas de malaise ou de détresse, l'étudiante au doctorat prendra toute les mesures nécessaires afin d'aider au mieux le participant. Si le malaise du participant dépasse ses compétences, le participant et son proche seront redirigés vers les ressources appropriées. Si le participant décide de retirer son consentement, il n'y aura aucune conséquence en ce qui a trait à l'accès et à la qualité des services reçus dans leur établissement de santé. En signant le formulaire de consentement, les participants ne renoncent à aucun droit et les chercheurs gardent leurs responsabilités légales et professionnelles à leur égard. Toutefois, aucune compensation financière ne sera donnée aux participants.

Retombées potentielles et conclusion

Bien que certains médicaments soient efficaces afin de retarder les symptômes moteurs de la MP, les effets sur la cognition chez les patients MP-TCL demeurent limités (Roy, Doiron, Talon-Croteau, Dupré et Simard, en préparation). Avec le vieillissement de la population, il a été estimé qu'entre 8,7 et 9,3 millions de personnes développeront la MP d'ici 2030 (Dorsey, Constantinescu, Thompson, Biglan, Holloway et al., 2007). Les avenues non-pharmacologiques pourraient constituer un bon complément à la médication, souvent plus sécuritaires et moins coûteuses pour le système de santé. De plus, ce genre d'intervention a le potentiel d'améliorer concrètement la vie des gens, en ciblant la bonification des AIVQ et en favorisant de ce fait l'autonomie des patients.

Échéancier

Année	Objectif	Fait?
**Année 1 (2015-2016)**		
Automne 2015<br>Hiver 2016	• Traduction du GMT et des questionnaires, et préparation de l'étude de cas à niveaux de base multiples (adaptation du programme pour des patients parkinsoniens).	X
Été 2016	• Rédaction du séminaire de projet.	X
**Année 2 (2016-2017)**		
Automne 2016	• Présentation du séminaire de projet au comité d'encadrement.	X
Hiver 2017	• Demande d'approbation éthique et préparation de l'étude de cas (si corrections à apporter lors de l'approbation éthique).	X
Été 2017	• Étude de cas à niveaux de base multiples avec deux patients, adaptation/modification du GMT, cueillette et analyse des données.	X
**Année 3 (2017-2018)**		
Automne 2017	• Rédaction et soumission pour publication de l'article 1 portant sur l'étude de cas.	½
Hiver 2018	• Préparation de l'étude randomisée – contrôle, approbation éthique à renouveler, recrutement, évaluation diagnostique des participants et début de l'entraînement GMT des participants.	X
	• Avril 2018 au plus tard: 1er séminaire évolutif.	X
Été 2018	• Entraînement GMT des participants.	
**Année 4 (2018-2019)**		
Automne 2018<br>Hiver 2019	• Suite et fin de l'étude randomisée – contrôle, analyse des résultats, rédaction et soumission de l'article 2.	
	• Avril 2019 au plus tard : 2e séminaire évolutif.	
Été 2019	• Rédaction de l'introduction et de la discussion générales de la thèse.	
	• Présentation du séminaire d'autorisation de dépôt de la thèse et dépôt initial de la thèse.	
**Année 5 (2019-2020)**		
Automne 2019, hiver 2020 et été 2020	• Internat.	

Références

Aarsland D. (2016). Cognitive impairment in Parkinson's disease and dementia with Lewy bodies. *Parkinsonism and Related Disorders*, *22 Suppl 1*, S144–8. doi:10.1016/j.parkreldis.2015.09.034

Aarsland D, Bronnick K, Alves G, Tysnes OB, Pedersen KF, Ehrt U, et Larsen JP. (2009). The spectrum of neuropsychiatric symptoms in patients with early untreated Parkinson's disease. *Journal of Neurology, Neurosurgery, and Psychiatry*, *80*(8), 928–930. doi:10.1136/jnnp.2008.166959

Aarsland D, Bronnick K, et Fladby T. (2011). Mild cognitive impairment in Parkinson's disease. *Current Neurology and Neuroscience Reports*, *11*(4), 371–378. doi:10.1007/s11910-011-0203-1

Aarsland D, Larsen JP, Lim NG, Janvin C Karlsen K, Tandberg E et Cummings JL. (1999). Range of neuropsychiatric disturbances in patients with Parkinson's disease. *Journal of Neurology Neurosurgery and psychiatry*; 67, 492-496.

Adler, CH, et Thorpy, MJ. (2005). Sleep issues in Parkinson's disease. *Neurology*, 64(12 suppl 3), S12-S20.

Alcaro A, Huber R, Panksepp J. (2007). Behavioral functions of the mesolimbic dopaminergic system: an affective neuroethological perspective. *Brain Res Rev*. 56, 283-321.

Anderson JR. (1982). Acquisition of cognitive skill. *Psychological review*, 89(4), 369.

Anderson JR., et Schunn C. (2000). Implications of the ACT-R learning theory: No magic bullets. Advances in instructional psychology, *Educational design and cognitive science*, 1-33.

Angelucci F, Peppe A, Carlesimo GA, Serafini F, Zabberoni S, Barban F, et al. (2015). A pilot study on the effect of cognitive training on BDNF serum levels in individuals with Parkinson's disease. *Frontiers Human Neuroscience*, *9*, 130. doi:10.3389/fnhum.2015.00130

Baddeley A. (2010). Working memory. *Current Biology*, 20(4), R136-R140.

Baddeley A, Sala SD, Papagno C, et Spinnler H. (1997). Dual-task performance in dysexecutive and nondysexecutive patients with a frontal lesion. *Neuropsychology*, Vol 11(2), 187-194. doi: http://dx.doi.org/10.1037/0894-4105.11.2.187

Barker RA, et Williams-Gray CH. (2015). Mild Cognitive Impairment and Parkinson's Disease-Something to Remember. *Journal of Parkinson's disease*, 4(4), 651-656.

Bastiaanse R et Lenders KL. (2009). Language and Parkinson's disease. *Cortex*; 25, 912-914.

Bédard M, Molloy W, Squire L, Dubois S, Lever JA, et O'Donnell M. (2001). The Zarit Burden Interview : a new short version and screening version. *Gerontologist*; 41(5), 652-657.

Bell-McGinty S, Podell K, Franzen M, Baird AD, et Williams MJ. (2002). Standard measures of execu- tive function in predicting instrumental activities of daily living in older adults. *International Journal of Geriatric Psychiatry*, 17(9), 828-834.

Benedict, RHB, Schretlen D, Groninger L, Dobraski M, et Shpritz B. (1996). Revision of the Brief Visuospatial Memory Test: Studies of normal performance, reliability, and validity. *Psychological Assessment*, 8(2), Jun 1996, 145-153.

Benton AL (1992). *Benton Visual Retention Test – revised* (5th ed.). San Antonio: The Psychological Corporation.

Benton AL (1994). *Contributions to neuropsychological assessment: A clinical manual*. Oxford University Press, USA.

Boller F, Passafiume D, Keefe NC, Rogers K, Morrow L, et Kim Y. (1984). Visual Impairements in Parkinson's disease: role of perceptual and motor factors. *Archives of Neurology*; 41, 485-490.

Bott NT, Johnson ET, Schuff N, Galifianakis N, Subas T, Pollock J, et al. (2014). Sensitive measures of executive dysfunction in non-demented Parkinson's disease. *Parkinsonism and Related Disorders*, *20*(12), 1430–1433. doi:10.1016/j.parkreldis.2014.10.007

Bora E, Walterfang M, et Velakoulis D. (2015). Theory of mind in Parkinson's disease: A meta-analysis. *Behavioural Brain Research*, 292, 515-520.

Braak H, Del Tredici K, Rüb U, de Vos RAI, Jansen Steur ENH et Braak E. (2003). Staging of brain pathology related to sporadic Parkinson's disease. *Neurobiology of Aging*, *24*(2), 197–211.

Bronnick K, Alves G, Aarsland D, Tysnes OB et Larsen JP. (2011). Verbal memory in drug naive, newly diagnosed Parkinson's disease: The retrieval hypothesis revisdited. *Neuropsychology*, 25 (1), 114-124.

Buschert V, Bokde ALW, et Hampel H. (2010). Cognitive intervention in Alzheimer disease. *Nature*, *6*(9), 508–517.
doi:10.1038/nrneurol.2010.113

Buschke H. (1984). Cued recall in amnesia. Journal of Clinical Neuropsychology, 6(4), 433-440.
doi: 10.1080/01688638408401 233

Cahn DA, Sullivan EV, Shear PK, Pfefferbaum A, Heit G, Silverberg G. (1998). Differential contributions of cognitive and motor component processes to physical and instrumental activities of daily living in Parkinson's disease. *Arch Clin Neuropsychol*, 13(7), 575-583.

Calleo J, Burrows C, Levin H, Marsh L, Lai E, et York MK. (2012). Cognitive rehabilitation for executive dysfunction in Parkinson's disease: application and current directions. *Parkinson's Disease*.

Cerasa A, Gioia MC, Salsone M, Donzuso G, Chiriaco C, Realmuto S, et al. (2014). Neurofunctional correlates of attention rehabilitation in Parkinson's disease: an explorative study. *Neurological Sciences*, *35*(8), 1173–1180.
doi:10.1007/s10072-014-1666-z

Chaudhuri KR, et Schapira AH. (2009.) Non-motor symptoms of Parkinson's disease: dopaminergic pathophysiology and treatment. *Lancet Neurol*. 8, 464-74.

Cheewakriengkrai L, et Gauthier S. (2013). A 10-year perspective on donepezil. *Expert Opinion on Pharmacotherapy*, *14*(3), 331–338. doi:10.1517/14656566.2013.760543

Cicchetti F, Drouin-Ouellet J, et Gross RE. (2009). Environmental toxins and Parkinson's disease: what have we learned from pesticide-induced animal models? *Trends in Pharmacological Sciences*, *30*(9), 475–483. doi:10.1016/j.tips.2009.06.005

Choi J, et Twamley EW. (2013). Cognitive rehabilitation therapies for Alzheimer's disease: a review of methods to improve treatment engagement and self-efficacy. *Neuropsychology review*, 23(1), 48-62.

Claassen DO, Josephs KA, Ahlskog JE, Silber MH, Tippmann-Peikert M, Boeve BF. (2010). REM sleep behavior disorder preceding other aspects of synuceinopathies by up to half a century. *Neurology*; 75:494–499.

Clare L, et Woods B. (2003). Cognitive rehabilitation and cognitive training for early-stage Alzheimer's disease and vascular dementia. *The Cochrane Library*.

Connolly BS, et Lang AE. (2014). Pharmacological treatment of Parkinson disease: a review. *Journal of the American Medical Association*, 311(16), 1670-1683.

Costa A, Peppe A, Serafini F, Zabberoni S, Barban F, Caltagirone C, et Carlesimo GA. (2014). Prospective memory performance of patients with Parkinson's disease depends on shifting aptitude: evidence from cognitive rehabilitation. *Journal of the International Neuropsychological Society*, *20*(7), 717–726. doi:10.1017/S1355617714000563

Cummings JL, Mega M, Gray K, Rosenberg-Thompson S, Carusi DA, et Gornbein J. (1994). The Neuropsychiatric Inventory comprehensive assessment of psychopathology in dementia. *Neurology*, 44(12), 2308-2308.

Dalrymple-Alford, JC, MacAskill MR, Nakas CT, Livingston L, Graham C, Crucian GP, & Porter RJ. (2010). The MoCA well-suited screen for cognitive impairment in Parkinson disease. Neurology, 75(19), 1717-1725.

Delis DC, Kramer JH, Kaplan E, et Ober BA. (2000). *California Verbal Learning Test – second edition. Adult version*. Manual. The Psychological corporation. San Antonio : TX.

Delis D, Kaplan E, et Kramer J. (2001). *Delis-Kaplan Executive Function System*. The Psychological Corporation, San Antonio, TX: Harcourt Brace et Company.

Del Tredici K, et Braak H. (2013). Dysfunction of the locus coeruleus-norepinephrine system and related circuitry in Parkinson's disease-related dementia. *Journal of Neurology, Neurosurgery, and Psychiatry*, *84*(7), 774–783. doi:10.1136/jnnp-2011-301817

De Medeiros K, Robert P, Gauthier S, Stella F, Politis A, Leoutsakos J, et Lyketsos C. (2010). The Neuropsychiatric Inventory-Clinician rating scale (NPI-C): reliability and validity of a revised assessment of neuropsychiatric symptoms in dementia. International Psychogeriatrics, 22(06), 984-994.

Dion M, Potvin O, Belleville S, Ferland G, Renaud M, Bherer L, . . . & Hudon, C. (2015). Normative data for the Rappel libre/Rappel indicé à 16 items (16-item Free and Cued Recall) in the elderly Quebec-French population. The Clinical Neuropsychologist, 28(Suppl.1), 1- 19. doi: 10.1080/13854046.2014.9 15058

Dirnberger G, et Jahanshahi M. (2013). Executive dysfunction in Parkinson's disease: a review. *Journal of Neuropsychology*, *7*(2), 193–224. doi:10.1111/jnp.12028

Doiron M, et Simard M. (2012). *Idiopathic Parkinson's disease, vascular risk factors and cognition: A critical review*.

Doiron M, Dupré N, Langlois M, Provencher P et Simard M. (Sous presse). Smoking history is associated to cognitive impairment in Parkinson's disease, *Aging et Mental Health*. DOI: 10.1080/13607863.2015.1090393

Dorsey ER, Constantinescu R, Thompson JP, Biglan KM, Holloway RG, Kieburtz K, Marshall FJ, Ravina BM, Schifitto G, Siderowf A, et Tanner CM. (2007). Projected number of people with Parkinson disease in the most populous nations, 2005 through 2030. *Neurology*. 68, 384-6.

Doyon J, Gaudreau D, Laforce RJ, Castonguay M, Bedard PJ, Bedard F, et Bouchard JP. (1997). Role of the striatum, cerebellum, and frontal lobes in the learning of a visuomotor sequence. Brain and cognition, 34(2), 218-245.

Dujardin K, Tard C, Duhamel A, Delval A, Moreau C, Devos D, et Defebvre L. (2013). The pattern of attentional deficits in Parkinson's disease. *Parkinsonism and Related Disorders*, *19*(3), 300–305. doi:10.1016/j.parkreldis.2012.11.001

Duncan J. (1986). Disorganisation of behaviour after frontal lobe damage. *Cognitive Neuropsychology*. doi:10.1080/02643298608253360

Edwards JD, Hauser RA, O'Connor ML, Valdés EG, Zesiewicz TA, et Uc EY. (2013). Randomized trial of cognitive speed of processing training in Parkinson disease. *Neurology*, *81*(15), 1284–1290. doi:10.1212/WNL.0b013e3182a823ba

Fearnley JM, Lees AJ. (1991). Ageing and Parkinson's disease: substantia nigra regional selectivity. *Brain*. 114 (Pt 5), 2283-301.

Folstein MF, Folstein SE, et McHugh PR. (1975). “Mini-mental state”: a practical method for grading the cognitive state of patients for the clinician. *J Psychiatric Res*; 12: 189-198.

Freedman M, et Stuss DT. (2011). Theory of Mind in Parkinson's disease. *Journal of the Neurological Sciences*, 310(1), 225-227.

Gallagher DA, O'Sullivan SS, Evans AH, Lees AJ, et Schrag A. (2007). Pathological gambling in Parkinson's disease: risk factors and differences from dopamine dysregulation. An analysis of published case series. *Movement Disorders,* 22(12), 1757-1763.

Gelb DJ, Oliver E, et Gilman S. (1999). Diagnostic criteria for Parkinson disease. *Archives of neurology*, 56(1), 33-39.

Gioia GA, Isquith PK, Guy SC et Kenworthy L. (2000). Test review behavior rating inventory of executive function. *Child Neuropsychology*, 6(3), 235-238.

Goetz CG, Poewe W, Rascol O, Sampaio C, Stebbins GT, Counsell C, Giladi N, Holloway RG, Moore CG, Wenning GK, Yahr MD, et Seidl L. (2004). Movement Disorder Society Task Force Report on the Hoehn and Yahr Staging Scale: Status and Recommendations: Movement Disorder Society Task Force on Rating Scales for Parkinson's Disease. *Movement Disorders*, 19(9), pp. 1020–1028.

Grandmaison É et Simard M. (2003). A critical review of memory stimulation programs in Alzheimer's disease. *The Journal of Neuropsychiatry and Clinical Neurosciences*, *15*(2), 130–144. doi:10.1176/jnp.15.2.130

Grober E, Buschke H, Crystal HA, Bang S, & Dresner R. (1988). Screening for dementia by memory testing. Neurology, 38(6), 900-903.
doi: 10.1212/WNL.38.6.900

Guidi M, Paciaroni L, Paolini S, Scarpino O, et Burn DJ. (2015). Semantic profiles in mild cognitive impairment associated with Alzheimer's and Parkinson's diseases. *Functional Neurology*, 30(2), 113.

Hawkins K, Jennings D, Marek K, Siderowf A, et Stern M. (2010). Cognitive deficits associated with dopamine transporter loss in the pre-motor subjects in the PARS cohort. *Movement Disorders*: (25), S690-S691.

Hawkins NG, Sanson-Fisher RW, Shakeshaft A, D'Este C et Green LW. (2007). The multiple baseline design for evaluating population-based research. *American Journal of Preventive Medicine*, *33*(2), 162–168.
doi:10.1016/j.amepre.2007.03.020

Hérbert R, Bravo G, et Préville M. (2000). Reliability, validity, and reference values of the Zarit Burden Interview for assessing informal caregivers of community-dwelling older persons with dementia. *Canadian Journal on Aging*, 19, 494-507.

Hindle JV, Martyr A, et Clare L. (2014). Cognitive reserve in Parkinson's disease: a systematic review and meta-analysis. *Parkinsonism and related disorders*, 20(1), 1-7.

Hindle JV, Petrelli A, Clare L, et Kalbe E. (2013). Nonpharmacological enhancement of cognitive function in Parkinson's disease: a systematic review. *Movement Disorders*, *28*(8), 1034–1049. doi:10.1002/mds.25377

Hoehn MM, et Yahr MD. (1967). Parkinsonism: onset, progression and mortality. *Neurology*; 17(5): 427-442.

Hooper HE. (1958). The Hooper Visual Organization Test: Manual. Los Angeles: Western Psychological Service.

Huckans, M., Hutson, L., Twamley, E., Jak, A., Kaye, J., et Storzbach, D. (2013). Efficacy of cognitive rehabilitation therapies for mild cognitive impairment (MCI) in older adults: working toward a theoretical model and evidence-based interventions. *Neuropsychology Review*, *23*(1), 63–80. doi:10.1007/s11065-013-9230-9

Hughes AJ, Daniel SE, Kilford L, et Lees AJ. (1992). Accuracy of clinical diagnosis of idiopathic Parkinson's disease: a clinico-pathological study of 100 cases. *Journal of Neurology, Neurosurgery, and Psychiatry*, *55*(3), 181–184. doi:10.1136/jnnp.55.3.181

Huntley JD, Gould RL, Liu K, Smith M, et Howard RJ. (2015). Do cognitive interventions improve general cognition in dementia? A meta-analysis and meta-regression. *BMJ Open*, *5*(4), e005247. doi:10.1136/bmjopen-2014-005247

Jellinger KA. (2006). The morphological basis of mental dysfunction in Parkinson's disease. *Journal of the Neurological Sciences*, *248*(1-2), 167–172. doi:10.1016/j.jns.2006.05.002

Jellinger KA. (2012). Neuropathology of sporadic Parkinson's disease: evaluation and changes of concepts. *Movement Disorders*, *27*(1), 8–30. doi:10.1002/mds.23795

Jellinger KA, et Attems J. (2015). Challenges of multimorbidity of the aging brain: a critical update. *Journal of Neural Transmission*, *122*(4), 505–521. doi:10.1007/s00702-014-1288-x

Kalbe E, et Kessler J. (2015). Task force WANTED: many reasons to promote research on cognitive rehabilitation to prevent, delay, and treat cognitive dysfunctions in patients with Parkinson's disease. *Parkinsonism and Related Disorders*, *21*(2), 166–167. doi:10.1016/j.parkreldis.2014.11.014

Kaplan E, Goodglass H, et Weintraub S. (2001). *Boston Naming Test*. Pro-edition.

Kawamura M, et Koyama S. (2007). Social cognitive impairment in Parkinson's disease. *Journal of Neurology*, 254(4), IV49-IV53.

Kehagia AA, Barker RA, et Robbins TW. (2010). Neuropsychological and clinical heterogeneity of cognitive impairment and dementia in patients with Parkinson's disease. *The Lancet Neurology*, *9*(12), 1200–1213. doi:10.1016/S1474-4422(10)70212-X

Kesner RP et Creem-Regehr SH. (2013). Parietal contributions to spatial cognition.

Kessels R, van Zandvoort M, Postma A, Kappelle LJ, et de Haan EH. (2000). The Corsi Block-Tapping Task: Standardization and Normative Data, 252-258. doi: 10.1207/S15324826AN0704_8

Kolb B, et Winshaw IQ. (2008). *Fundamentals of Human Neuropsychology (6th edition)*. Worth Publisher. 818 p.

Kudlicka A, Clare L, et Hindle JV. (2011). Executive functions in Parkinson's disease: Systematic review and meta-analysis. *Movement Disorders*, 26(13), 2305-2315.

Kulisevsky J, Fernández de Bobadilla R, Pagonabarraga J, Martínez-Horta S, Campolongo A, García-Sánchez C, et al. (2013). Measuring functional impact of cognitive impairment: validation of the Parkinson's disease cognitive functional rating scale. *Parkinsonism and Related Disorders*, *19*(9), 812–817.
doi:10.1016/j.parkreldis.2013.05.007

Laforce R, et Doyon J. (2001). Distinct contribution of the striatum and cerebellum to motor learning. *Brain and cognition*, 45(2), 189-211.

Lavoie M, Callahan B, Belleville S, Simard M, Bier N, Gagnon L, Gagnon J-F, Blanchet S, Potvin O, Hudon C, Macoir J. (2013) Normative data for the Dementia Rating Scale in the French-Quebec Population. *The Clinical Neuropsychologist*; 27(7): 1150-1166.

Lawson RA, Yarnall AJ, Duncan GW, Breen DP, Khoo TK, Williams-Gray CH, ICICLE-PD study group. (2016). Cognitive decline and quality of life in incident Parkinson's disease: The role of attention. Parkinsonism et related disorders.

Lawson RA, Yarnall AJ, Duncan GW, Khoo TK, Breen DP, Barker RA, et al. (2014). Severity of mild cognitive impairment in early Parkinson's disease contributes to poorer quality of life. *Parkinsonism and Related Disorders*, *20*(10), 1071–1075.
doi:10.1016/j.parkreldis.2014.07.004

Lees AJ, Hardy J, et Revesz T. (2009). Parkinson's disease. *The Lancet*, *373*(9680), 2055–2066. doi:10.1016/S0140-6736(09)60492-X

Leentjens AF, Dujardin K, Marsh L, Martinez‐Martin P, Richard IH, Starkstein SE et Stebbins GT. (2008). Apathy and anhedonia rating scales in Parkinson's disease: critique and recommendations. Movement Disorders, 23(14), 2004-2014. Lesage, S., Brice, A., 2009. Parkinson's disease: from monogenic forms to genetic susceptibility factors. *Hum Mol Genet.* 18, R48-59.

Leung IH, Walton CC, Hallock H, Lewis SJ, Valenzuela M, et Lampit A. (2015). Cognitive training in Parkinson disease A systematic review and meta-analysis. *Neurology*, 85(21), 1843-1851.

Levine B, and Downey-Lamb MM. (2005). *Design and evaluation of rehabilitation experiments. Neuropsychological interventions: clinical research and practice*. Eslinger PJ. New York: The Guilford Press.

Levine B, Robertson IH, Clare L, Carter G, Hong J, Wilson BA, et al. (2000). Rehabilitation of executive functioning: an experimental-clinical validation of goal management training. *Journal of the International Neuropsychological Society*, *6*(3), 299–312.

Levine B, Schweizer TA, O'Connor C, Turner G, Gillingham S, Stuss DT, et al. (2011). Rehabilitation of Executive Functioning in Patients with Frontal Lobe Brain Damage with Goal Management Training. *Frontiers Human Neuroscience*, *5*. doi:10.3389/fnhum.2011.00009

Levine B, Stuss DT, Winocur G, Binns MA, Fahy L, Mandic M, et al. (2007). Cognitive rehabilitation in the elderly: effects on strategic behavior in relation to goal management. *Journal of the International Neuropsychological Society*, *13*(1), 143–152. doi:10.1017/S1355617707070178

Liepelt-Scarfone I, Gräber S, Fruhmann Berger M, Feseker A, Baysal G, Csoti I, et al. (2012). Cognitive profiles in Parkinson's disease and their relation to dementia: a data-driven approach. *International Journal of Alzheimer's Disease*, *2012*, 910757. doi:10.1155/2012/910757

Litvan I, Aarsland D, Adler CH, Goldman JG, Kulisevsky J, Mollenhauer B, et al. (2011). MDS Task Force on mild cognitive impairment in Parkinson's disease: critical review of PD-MCI. *Movement Disorders*, *26*(10), 1814–1824. doi:10.1002/mds.23823

Litvan I, Goldman JG, Tröster AI, Schmand BA, Weintraub D, Petersen RC, et al. (2012). Diagnostic criteria for mild cognitive impairment in Parkinson's disease: Movement Disorder Society Task Force guidelines. *Movement Disorders*, *27*(3), 349–356. doi:10.1002/mds.24893

Marin RS, Biedrzycki RC & Firinciogullari S. (1991). Reliability and validity of the Apathy Evaluation Scale. *Psychiatry research*, 38(2), 143-162.

Matteau E, Dupré N, Langlois M, Provencher P, et Simard M. (2012). Clinical validity of the Mattis Dementia Rating Scale-2 in Parkinson disease with MCI and dementia. *Journal of Geriatric Psychiatry and Neurology*, *25*(2), 100–106. doi:10.1177/0891988712445086

Mattis S. (2001). Dementia Rating Scale-2. Lutz, Florida: Psychological Assessment Ressources Inc.

McCaffrey RJ, Ortega A, et Haase RF. (1993). Effects of repeated neuropsychological assessments. *Archives of Clinical Neuropsychology*, 8(6), 519-524.

McGraw KO, et Wong SP. (1992). A Common Language Effect Size Statistic. *Psychological Bulletin*, 361–365.

Milman U, Atias H, Weiss A, Mirelman A, et Hausdorff JM. (2014). Can cognitive remediation improve mobility in patients with Parkinson's disease? Findings from a 12 week pilot study. *Journal of Parkinson's Disease*, *4*(1), 37–44. doi:10.3233/JPD-130321

Mohlman J, Chazin D, et Georgescu B. (2011). Feasibility and acceptance of a nonpharmacological cognitive remediation intervention for patients with Parkinson disease. *Journal of Geriatric Psychiatry*

and Neurology, *24*(2), 91–97. doi:10.1177/0891988711402350

Monchi O, Hanganu A, et Bellec P. (2016). Markers of cognitive decline in PD: the case for heterogeneity. *Parkinsonism and Related Disorders*.

Müller MT, et Bohnen NI. (2013). Cholinergic dysfunction in Parkinson's disease. *Current Neurology and Neuroscience Reports*, *13*(9), 377. doi:10.1007/s11910-013-0377-9

Nasreddine ZS, Phillips NA, Bedirian V, Charbonneau S, Whitehead V, Collin I, et al. (2005). The Montreal Cognitive Assessment, MoCA: a brief screening tool for mild cognitive impairment. *J Am Geriatr Soc*, 53, 695-699.

Nombela C, Bustillo PJ, Castell PF, Sanchez L, Medina V, et Herrero MT. (2011). Cognitive rehabilitation in Parkinson's disease: evidence from neuroimaging. *Frontiers in Neurology*, *2*, 82. doi:10.3389/fneur.2011.00082

Norman DA et Shallice T. (1986). Attention to action (pp. 1-18). Springer US.

Pagano G, Rengo G, Pasqualetti G, Femminella GD, Monzani F, Ferrara N, et Tagliati M. (2015). Cholinesterase inhibitors for Parkinson's disease: a systematic review and meta-analysis. *Journal of Neurology, Neurosurgery, and Psychiatry*, *86*(7), 767–773. doi:10.1136/jnnp-2014-308764

Pagonabarraga J, et Kulisevsky J. (2012). Cognitive impairment and dementia in Parkinson's disease. *Neurobiology of Disease*, *46*(3), 590–596. doi:10.1016/j.nbd.2012.03.029

Pagonabarraga J, Kulisevsky J, Llebaria G, García ‐ Sánchez C, Pascual ‐ Sedano B, et Gironell A. (2008). Parkinson's disease ‐ cognitive rating scale: A new cognitive scale specific for Parkinson's disease. *Movement Disorders*, 23(7), 998-1005.

Palavra NC, Naismith, SL, et Lewis, SJ. (2013). Mild cognitive impairment in Parkinson's disease: a review of current concepts. *Neurology Research International*.

Pan-Montojo F, et Reichmann H. (2014). Considerations on the role of environmental toxins in idiopathic Parkinson's disease pathophysiology. *Translational Neurodegeneration*, *3*(1), 10. doi:10.1186/2047-9158-3-10

París AP, Saleta HG, de la Cruz Crespo Maraver M, Silvestre E, Freixa MG, Torrellas CP, et al. (2011). Blind randomized controlled study of the efficacy of cognitive training in Parkinson's disease. *Movement Disorders*, *26*(7), 1251–1258. doi:10.1002/mds.23688

Parkinson J. (1817). An essay on the shaking palsy. Whittingham and Rowland, for Sherwood, Neely, and Jones. London.

Parnetti L, Calabresi P. (2006). Spatial cognition in Parkinson's disease and neuro- degenerative dementias. *Cogn Process;*7: S77e8.

Peña J, Ibarretxe-Bilbao N, García-Gorostiaga I, Gomez-Beldarrain MA, Díez-Cirarda M, et Ojeda N. (2014). Improving functional disability and cognition in Parkinson disease: randomized controlled trial. *Neurology*, *83*(23), 2167–2174. doi:10.1212/WNL.0000000000001043

Péron J, Vicente S, Leray E, Drapier S, Drapier D, Cohen R, et Vérin M. (2009). Are dopaminergic pathways involved in theory of mind? A study in Parkinson's disease. *Neuropsychologia*, *47*(2), 406-414.

Petersen RC. (2004). Mild cognitive impairment as a diagnostic entity. *Journal of Internal Medicine*, 256(3), 183-194.

Petersen RC, et Morris JC. (2005). Mild cognitive impairment as a clinical entity and treatment target. *Archives of Neurology*, 62(7), 1160-1163.

Peto V, Jenkinson C, et Fitzpatrick R. (1998). PDQ-39: A review of the development, validation and application of a Parkinson's disease quality of life questionnaire and its associated measures. *Journal of Neurology*, 245(Suppl. 1), S10–S14. doi:10.1007/PL00007730

Petrelli A, Kaesberg S, Barbe MT, Timmermann L, Fink GR, Kessler J, et Kalbe E. (2014). Effects of cognitive training in Parkinson's disease: a randomized controlled trial. *Parkinsonism and Related Disorders*, *20*(11), 1196–1202. doi:10.1016/j.parkreldis.2014.08.023

Petrelli A, Kaesberg S, Barbe MT, Timmermann L, Rosen JB, Fink GR, et al. (2015). Cognitive training in Parkinson's disease reduces cognitive decline in the long term. *European Journal of Neurology*, *22*(4), 640–647. doi:10.1111/ene.12621

Pirogovsky E, Schiehser DM, Obtera KM, Burke MM, Lessig SL, Song DD, et al. (2014). Instrumental activities of daily living are impaired in Parkinson's disease patients with mild cognitive impairment. *Neuropsychology*, *28*(2), 229–237. doi:10.1037/neu0000045

Poletti M, et Bonuccelli U. (2013). Acute and chronic cognitive effects of levodopa and dopamine agonists on patients with Parkinson's disease: a review. *Therapeutic Advances in Psychopharmacology*, *3*(2), 101–113. doi:10.1177/2045125312470130

Poletti M, Emre M, et Bonuccelli U. (2011a). Mild cognitive impairment and cognitive reserve in Parkinson's disease. *Parkinsonism and Related Disorders*, *17*(8), 579–586. doi:10.1016/j.parkreldis.2011.03.013

Poletti M, Enrici I, Bonuccelli U, Adenzato M. (2011b). Theory of Mind in Parkinson's disease. Behavioural

Brain Research, 319, 342-350.

Politis, M., et Niccolini, F. (2015). Serotonin in Parkinson's disease. *Behavioural Brain Research*, *277*, 136–145.
doi:10.1016/j.bbr.2014.07.037

Postuma RB, Berg D, Stern M, Poewe W, Olanow CW, Oertel W, et Halliday G. (2015). MDS clinical diagnostic criteria for Parkinson's disease. *Movement Disorders*, 30(12), 1591-1601.

Premack DG, et Woodruff G. (1978). Does the chimpanzee have a Theory of Mind? *Behavioural Brain Science*; 1: 515–26.

Reuter I, Mehnert S, Sammer G, Oechsner M, et Engelhardt M. (2012). Efficacy of a multimodal cognitive rehabilitation including psychomotor and endurance training in Parkinson's disease. *Journal of Aging Research*, *2012*, 235765.
doi:10.1155/2012/235765

Robben, S. H., Sleegers, M. J., Dautzenberg, P. L., van Bergen, F. S., ter Bruggen, J. P., & Rikkert, M. G. (2010). Pilot study of a three‐step diagnostic pathway for young and old patients with Parkinson's disease dementia: screen, test and then diagnose. International journal of geriatric psychiatry, 25(3), 258-265.

Robbins TW et Cools R. (2014). Cognitive deficits in Parkinson's disease: a cognitive neuroscience perspective. *Movement Disorders*, 29(5), 597-607.

Robertson IH. (1996). *Goal Management Training: a clinical manual*. Cambridge, UK : PsyConsult.

Roca M, Torralva T, Gleichgerrcht E, Chade A, Arévalo GG, Gershanik O et Manes F. (2010). Impairments in social cognition in early medicated and unmedicated Parkinson disease. *Cognitive and Behavioral Neurology*, *23*(3), 152-158.

Rolinski M, Fox C, Maidment I et McShane R. (2012). Cholinesterase inhibitors for dementia with Lewy bodies, Parkinson"s disease dementia and cognitive impairment in Parkinson"s disease. *The Cochrane Database of Systematic Reviews*, *3*, CD006504.
doi:10.1002/14651858.CD006504.pub2

Rosenthal E, Brennan L, Xie S, Hurtig H, Milber J, Weintraub D, et al. (2010). Association between cognition and function in patients with Parkinson disease with and without dementia. *Movement Disorders*, *25*(9), 1170–1176.
doi:10.1002/mds.23073

Roy M-A, Doiron M, Talon-Croteau J, Dupré N, et Simard M. (En préparation). Effects of Levodopa and other Dopaminergic Treatments on Cognition in Cognitively Intact and Mildly Impaired Parkinson's Disease Patients : A Systematic Review.

Sai Y, Zou Z, Peng K, et Dong Z. (2012). The Parkinson's disease-related genes act in mitochondrial homeostasis. *Neuroscience and Biobehavioral Reviews*, *36*(9), 2034–2043.
doi:10.1016/j.neubiorev.2012.06.007

Sala SD, Baddeley A, Papagno C, et Spinnler H. (1995). Dual ‐ task paradigm: a means to examine the central executive. *Annals of the New York Academy of Sciences*, 769(1), 161-172.

Salmon DP, Thal LJ, Butters N et Heindel WC. (1990). Longitudinal evaluation of dementia of the Alzheimer type A comparison of 3 standardized mental status examinations. *Neurology*, 40(8), 1225.

Sammer G, Reuter I, Hullmann K, Kaps M, et Vaitl D. (2006). Training of executive functions in Parkinson's disease. *Journal of the Neurological Sciences*, *248*(1-2), 115–119. doi:10.1016/j.jns.2006.05.028

Schapira AH, Jenner P. (2011). Etiology and pathogenesis of Parkinson's disease. *Movement Disorder.* 26, 1049-55.

Schmidt M. (1996). *Rey auditory verbal learning test: a handbook*. Los Angeles: Western Psychological Services.

Schrag A, Jahanshahi M, et Quinn N. (2000). What contributes to quality of life in patients with Parkinson's disease? *Journal of Neurology, Neurosurgery and Psychiatry*, 69, 308–312.

Schweizer TA, Levine B, Rewilak D, O'Connor C, Turner G, Alexander MP, et al. (2008). Rehabilitation of executive functioning after focal damage to the cerebellum. *Neurorehabilitation and Neural Repair*, *22*(1), 72–77. doi:10.1177/1545968307305303

Sitzer DI, Twamley EW et Jeste DV. (2006). Cognitive training in Alzheimer's disease: a meta-analysis of the literature. *Acta Psychiatrica Scandinavica*, 114(2), 75-90.

Shulman LM, Pretzer-Aboff I, Anderson KE, Stevenson R, Vaughan CG, Gruber-Baldini AL et Weiner WJ. (2006). Subjective report versus objective measurement of activities of daily living in Parkinson's disease. *Movement Disorders*, 21(6), 794-799.

Shulman LM, Gruber-Baldini AL, Anderson KE, Vaughan CG, Reich SG, Fishman PS, et Weiner WJ. (2008). The evolution of disability in Parkinson disease. *Movement Disorders*, 23(6), 790-796.

Smith EE, et Jonides J. (1999). Storage and executives processes in the frontal lobes. Science; 283 (5408): 1657-1661.

Sofi F, Cesari F, Abbate R, Gensini GF, et Casini A. (2008). Adherence to Mediterranean diet and health status: meta-analysis. *BMJ*, *337*(sep11 2), a1344–a1344. doi:10.1136/bmj.a1344

Sollinger AB, Goldstein FC, Lah JJ, Levey AI, et Factor SA. (2010). Mild cognitive impairment in Parkinson's disease: subtypes and motor characteristics. *Parkinsonism and Related Disorders*, *16*(3), 177–180. doi:10.1016/j.parkreldis.2009.11.002

St-Hilaire A, Hudon C, Vallet G, Bherer L, Lussier M, Gagnon JF, Simard M, Gosselin N, Escudier F, Rouleau I et Macoir J. (Sous presse). Normative Data for Phonemic and Semantic Verbal Fluency Test in the Adult French-Quebec Population and Validation Study in Alzheimer's Disease and Depression. *The Clinical Neuropsychologist*: 30(1).

Stahl SM. (2008). *Stahl's essential psychopharmacology: neuroscientific basis and practical applications*. Troisième édition. Cambridge University Press, New York, 1117 pp.

Stern Y. (2002). What is cognitive reserve? Theory and research application of the reserve concept. *Journal of the International Neuropsychological Society*, 8(03), 448-460.

Stone VE, Baron-Cohen S et Knight RT. (1998). Frontal lobe contributions to theory of mind. *Journal of Cognitive Neuroscience,* 10, 640-656.

Strauss E, Sherman EM et Spreen O. (2006). Report writing and feedback session. Dans *A compendium of Neuropsychological Tests: Administration, Norms, and Commentary* (Third Edition). Oxford University Press; pp 86-97.

Stubberud J, Langenbahn D, Levine B, Stanghelle J, et Schanke AK. (2013). Goal management training of executive functions in patients with spina bifida: a randomized controlled trial. *Journal of the International Neuropsychological Society*, *19*(6), 672–685. doi:10.1017/S1355617713000209

Tanner CM. (1989). The role of environmental toxins in the etiology of Parkinson's disease. *Trends in Neurosciences*, *12*(2), 49–54. doi:10.1016/0166-2236(89)90135-5

Thivierge S, Jean L, Vézina J et Simard M. (2014). A Randomized Cross-over Controlled Study on Cognitive Rehabilitation of Instrumental Activities of Daily Living in Alzheimer Disease. *Physical Comorbidity*, *22*(11), 1188–1199. doi:10.1016/j.jagp.2013.03.008

Thivierge S, Simard M, Jean L, et Grandmaison É. (2008). Errorless learning and spaced retrieval techniques to relearn instrumental activities of daily livingin mild Alzheimer's disease: A case report study. *Neuropsychiatric Disease and Treatment*, *4*(5), 987–999.

Tröster AI. (2011). A précis of recent advances in the neuropsychology of mild cognitive impairment(s) in Parkinson's disease and a proposal of preliminary research criteria. *Journal of the International Neuropsychological Society*, *17*(3), 393–406. doi:10.1017/S1355617711000257

Tulving E. (1995). Organization of memory: Quo vadis. The Cognitive Neurosciences.

Van der Linden M, Coyette F, Poitrenaud J, Kalafat M, Calicis F, Wyns C, . . . Membres du GREMEM. (2004). L'épreuve de rappel libre/ rappel indicé à 16 items (RL/RI-16). In M. Van der Linden, S. Adam, A. Agniel, & Membres du GRENEM (Eds.), L'évaluation des troubles de la mémoire : présentation de quatre tests de mémoire épisodique avec leur étalonnage. (pp. 25-47). Marseille: Solal

van Hooren S, Valentijn S, et Bosma H. (2007). Effect of a structured course involving goal management training in older adults: A randomised controlled trial. *Patient Education*, *65*(2), 205–213. doi:10.1016/j.pec.2006.07.010

Van Zomeren, AH, et Brouwer WJ. (1994). Theories and concepts of attention. Clinical neuropsychology

of attention (2), 7-38.

Vertstichel P et Cambier J. (2005). *Les aphasies*, dans Neuropsychologie et neurologie clinique du comportement, 3e édition. Presses de l'université de Montréal. 455-486

Weintraub D, Simuni T, Caspell-Garcia C, Coffey C, Lasch S, Siderowf A, et al. (2015). Cognitive performance and neuropsychiatric symptoms in early, untreated Parkinson's disease. *Movement Disorders*, *30*(7), 919–927. doi:10.1002/mds.26170

Weintraub D et Burn DJ. (2011). Parkinson's disease: the quintessential neuropsychiatric disorder. *Movement Disorders*, 26(6), 1022-1031.

Wechsler D. (2008). Wechsler Adult Intelligence Scale-Fourth Edition. San Antonio, TX: Pearson.

Williams JR, Hirsch ES, Anderson K, Bush AL, Goldstein SR, Grill S, et Pontone, G. (2012). A comparison of nine scales to detect depression in Parkinson disease Which scale to use? *Neurology*, 78(13), 998-1006.

Williams-Gray CH, Foltynie T, Brayne CEG, Robbins TW, et Barker RA. (2007). Evolution of cognitive dysfunction in an incident Parkinson's disease cohort. *Brain*, *130* (7), 1787–1798. doi:10.1093/brain/awm111

Wilson B, Cockburn J, Baddeley A, et Hiorns R. (1989). The development and validation of a test battery for detecting and monitoring everyday memory problems. *Journal of Clinical and Experimental Neuropsychology*, 11(6), 855-870.

Wilson BA, Alderman N, Burgess PW, Emslie H, Evans JJ. (1996). *The behavioural assessment of the dysexecutive syndrome*. Thames Valley Company; Bury St Edmunds.

Wirdefeldt K, Adami HO, Cole P, Trichopoulos D, Mandel J. (2011). Epidemiology and etiology of Parkinson's disease: a review ofthe evidence. *Eur J Epidemiol.* (26) Suppl 1, S1- 58.

Woods B, Aguirre E, Spector AE, et Orrell M. (2012). Cognitive stimulation to improve cognitive *functioning in people with dementia. Cochrane Database Syst Rev, 2.*

Zeighami, Y., Ulla, M., Iturria-Medina, Y., Dadar, M., Zhang, Y., Larcher, K. M. H., et Dagher, A. (2015). Network structure of brain atrophy in de novo Parkinson's disease. *E-Life, 4, e08440.*

Zimmermann R, Gschwandtner U, Benz N, Hatz F, Schindler C, Taub E, et Fuhr P. (2014). Cognitive training in Parkinson disease: cognition-specific vs nonspecific computer training. *Neurology*, *82*(14), 1219–1226. doi:10.1212/WNL.0000000000000287

Zimprich A, Biskup S, Leitner P, Lichtner P, Farrer M, Lincoln S, Kachergus J, Hulihan M, Uitti RJ, Calne DB, Stoessl AJ, Pfeiffer RF, Patenge N, Carbajal IC, Vieregge P, Asmus F, Muller-Myhsok B, Dickson DW, Meitinger T, Strom TM, Wszolek ZK, Gasser T. (2004). Mutations in LRRK2 cause autosomal-dominant parkinsonism with pleomorphic pathology. *Neuron*. 44, 601-7.

ANNEXE 1

Tableau 1.1 - Critères diagnostiques de la MP idiopathique selon la «*United Kingdom Parkinson's Disease Society Brain Bank*» (Hughes et al., 1992)

**1. Diagnostic du syndrôme parkinsonien**
**Bradykinésie** (lenteur dans les mouvements volontaires avec réduction progressive de la vitesse et de l'amplitude des mouvements répétitifs).<br><br>**Au moins un autre symptôme parmi :**<br><br>• Rigidité musculaire,<br>• Tremblements au repos de l'ordre de 4-6 Hz,<br>• Instabilité posturale qui n'est pas explicable par une dysfonction visuelle, vestibulaire, cérébelleuse ou proprioceptive.
**2. Critères d'exclusion de la MP idiopathique**
• Historique d'infarctus répétés avec progression atypique des symptômes parkinsoniens,<br>• Historique de traumatismes crâniens répétés,<br>• Historique d'encéphalite confirmée,<br>• Crises oculogyres,<br>• Traitements aux neuroleptiques lors de l'apparition des symptômes,<br>• Plus d'un proche atteint des symptômes,<br>• Rémission spontanée,<br>• Symptômes uniquement unilatéraux après 3 ans,<br>• Paralysie supra-nucléaire,<br>• Signes neurologiques focaux,<br>• Dysfonction sévère du système nerveux autonome en début de maladie,<br>• Démence sévère avec troubles de la mémoire, du langage ou des praxies en début de maladie,<br>• Présence du réflexe de Babinski,<br>• Présence d'une tumeur cérébrale ou d'une hydrocéphalie marquée selon des résultats de neuroimagerie,<br>• Réponse négative à la L-DOPA (excluant une malabsorption),<br>• Exposition à la MPTP.
**3. Autres critères supportant le diagnostic (au moins 3 symptômes pour poser un diagnostic de MP idiopathique bien défini)**
• Début unilatéral,<br>• Présence de tremblements au repos,<br>• Développement progressif,<br>• Asymétrie persistante affectant le même côté qu'au début des symptômes,<br>• Réponse excellente à la L-DOPA (70% À 100%),

- Réponse à la L-DOPA pendant 5 ans ou plus,
- Développement clinique s'étalant sur 10 ans ou plus.

Tableau 1.2 - Critères diagnostiques de la MP idiopathique selon la «*National Institute of Neurological Disorders and Stroke*» (Gelb et al., 1999)

Groupe A : Caractéristiques de la MP
• Tremblements au repos,<br>• Bradykinésie,<br>• Rigidité,<br>• Début des symptômes asymétrique.
**Groupe B : Caractéristiques suggérant d'autres diagnostics possibles**
**Lorsque tôt dans les symptômes cliniques :**<br>• Instabilité posturale dominante au cours des trois premières années,<br>• Phénomène de «*freezing*» au cours de trois premières années,<br>• Hallucinations non-liées à la médication,<br>• Développement d'une démence avant les symptômes moteurs ou bien au cours de la première année,<br>• Paralysie supra-nucléaire, ou ralentissement des saccades visuelles,<br>• Dysfonction sévère du système nerveux autonome en début de maladie,<br>• Autre cause médicale pouvant mieux expliquer les symptômes de parkinsonisme, tel qu'une lésion cérébrale ou une tumeur au cerveau.
**Critères pour un diagnostic possible de MP idiopathique**
• Au moins deux des symptômes mentionnés en A, dont au moins un est la bradykinésie ou les tremblements,<br>• Aucune des caractéristiques du groupe B n'est présente au moins 3 ans après le début des symptômes,<br>• Bonne réponse à la L-DOPA ou à un agoniste dopaminergique.

Tableau 1.3 - Critères diagnostiques de la MP idiopathique selon la «*Movement Disorder Society*» (Postuma et al., 2015)

**Caractéristiques supportant le diagnostic de MP**
**Critères obligatoires : syndrome de parkinsonisme**<br>• Bradykinésie,<br>• Rigidité,<br>• Tremblements au repos.<br><br>**Autres caractéristiques supportant le diagnostic**<br>• Réponse franche et claire à une thérapie dopaminergique,<br>• Présence de dyskinésie induite par la L-DOPA,<br>• Tremblement au repos d'un membre selon une évaluation clinique,<br>• Présence d'anosmie (perte de l'olfaction) ou de dénervation cardiaque selon une scintigraphie.
**Caractéristiques excluant le diagnostic de MP**
**Caractéristiques excluant automatiquement le diagnostic de MP**<br>• Dysfonction sévère du système nerveux autonome en début de maladie,<br>• Paralysie supra-nucléaire, ou ralentissement des saccades visuelles,<br>• Diagnostic probable d'une démence fronto-temporale ou d'une aphasie primaire progressive au cours des cinq premières années,<br>• Syndrome de parkinsonisme restreint aux membres inférieurs pour au moins les trois premières années,<br>• Médication anti-dopaminergique qui concorde avec l'apparition des symptômes de parkinsonisme,<br>• Absence de réponse à des fortes doses de L-DOPA, même lorsque les symptômes moteurs sont modérés ou sévères,<br>• Perte de sensations au niveau cortical (aphasie progressive, agraphie, perte de l'usage d'un seul membre, etc.),<br>• Fonctionnement normal du système dopaminergique présynaptique, selon une imagerie fonctionnelle,<br>• Autre cause médicale pouvant mieux expliquer les symptômes de parkinsonisme, tel qu'une lésion cérébrale ou une tumeur au cerveau.<br><br>**Caractéristiques pouvant suggérer un autre diagnostic que celui de MP**<br>• Progression rapide de l'instabilité posturale demandant l'utilisation d'une chaise roulante moins de cinq ans suite au diagnostic,<br>• Absence complète de progression des symptômes moteurs pendant au moins cinq ans sauf si reliée au traitement pharmacologique,<br>• Dysfonction nerveuse sévère affectant la production du langage : dysarthrie, dysphonie ou dysphagie sévères, moins de cinq ans suite au diagnostic,<br>• Dysfonction de la respiration (surtout inspiration), que ce soit diurne ou nocturne,<br>• Défaillance sévère du système nerveux autonome moins de cinq ans suite au diagnostic, par exemple :<br>◦ Hypotension orthostatique,<br>◦ Rétention urinaire sévère, ou incontinence urinaire sévère moins de trois ans suite au diagnostic,<br>• Chutes récurrentes en raison de graves problèmes d'équilibre moins de trois ans suite au diagnostic,

- Contractions disproportionnées des pieds ou des mains moins de 10 ans suite au diagnostic,
- Absence des symptômes non-moteurs fréquemment associés à la MP moins de cinq ans suite au diagnostic, tels que :
    - Problèmes de sommeil (trouble du sommeil paradoxal, insomnie, etc.),
    - Dysfonction légère du système nerveux autonome (constipation, urgences urinaires diurnes, etc.)
    - Anosmie
    - Symptômes psychologiques et comportementaux (dépression, apathie, anxiété, etc.)
- Autres symptômes pyramidaux non-expliqués,
- Parkinsonisme bilatéral, rapporté par le patient, un proche ou le clinicien.

Types de diagnostics de MP

MP cliniquement établie :

- Absence de caractéristique excluant automatiquement le diagnostic de MP,
- Au moins deux critères supportant le diagnostic de MP,
- Absence de caractéristique pouvant suggérer un autre diagnostic que celui de MP.

MP cliniquement probable :

- Absence de caractéristique excluant automatiquement le diagnostic de MP,
- Au moins deux critères supportant le diagnostic de MP,
- Présence d'au plus deux caractéristiques pouvant suggérer un autre diagnostic que celui de MP, mais il doit y avoir au plus autant de ces caractéristiques que de caractéristiques supportant le diagnostic. Donc, s'il y a présence de deux caractéristiques pouvant suggérer un autre diagnostic, il doit y avoir présence de deux caractéristiques supportant le diagnostic.

Tableau 1.4 – Sommaire de tests neuropsychologiques suffisamment sensibles pour l'évaluation du TCL dans la MP

Domaine cognitif	Tests neuropsychologiques recommandés
**Attention et mémoire de travail**	• Empan endroit, envers et ordre croissant de la WAIS-IV (Weschler, 2008).<br>• Séquences lettres-chiffres de la WAIS-IV (Weschler, 2008).<br>• Trail Making Test (TMT) de la D-KEFS, conditions 1-3 (Delis, Kaplan et Kramer, 2001).<br>• Test de Stroop, version de la D-KEFS (Delis, Kaplan et Kramer, 2001).
**Fonctions exécutives**	• Test de fluidité verbale, particulièrement la condition d'alternance, version de la D-KEFS (Delis, Kaplan et Kramer, 2001).<br>• «*Behavioral Assesment of Dysexecutive Syndrome*» (BADS), une batterie regroupant plusieurs tests écologiques (Wilson et al., 1996).
**Langage**	• Test de dénomination sous confrontation, comme le test de dénomination de Boston (Kaplan, Goodglass et Weintraub, 1983).
**Mémoire épisodique**	• Liste de mots à apprendre avec rappel immédiat et différé, comme le «*California Verbal Learning Test*» (CVLT) (Delis, Kramer, Kaplan et Ober, 2000) ou le «*Rey Auditory Verbal Learning Test*» (RAVLT) (Schmidt, 1996).<br>• Mémoire épisodique visuelle: «*Brief Visuospatial Memory Test – Revised*» (Benedict et al., 1996).
**Fonctions visuo-spatiales**	• Test de jugement d'orientation des lignes de Benton (Benton, 1992).<br>• Test d'organisation visuelle de Hooper (Hooper, 1958).
**Cognition globale**	• MoCA (Nasreddine et al., 2005) ou MMSE (Folstein et al., 1975).<br>• Échelle de démence de Mattis-2ième édition (Mattis, 2001).

ANNEXE 2

Les stades cliniques de la MP de Hoehn et Yahr (1967) révisés (Goetz et al., 2004)

Stade	Description
1	Symptômes moteurs unilatéraux uniquement, souvent au niveau des membres supérieurs
1.5	Mouvements unilatéraux et axiaux, le tronc est aussi pris de tremblements involontaires
2	Problèmes bilatéraux du mouvement, mais sans problème d'équilibre
2.5	Problèmes bilatéraux du mouvement avec certaines difficultés d'équilibre, mais récupération au test de poussée
3	Difficultés bilatérales légères ou modérées et un peu d'instabilité posturale, mais indépendance physique préservée
4	Difficultés plus sévères, mais capacité à se tenir debout et déplacements à l'aide d'une marchette ou d'une canne
5	Déplacements en chaise roulante et aide nécessaire dans les soins de base et les activités quotidiennes

ANNEXE 3

L'arbre décisionnel de Petersen (2004) pour caractériser le TCL (traduction libre)

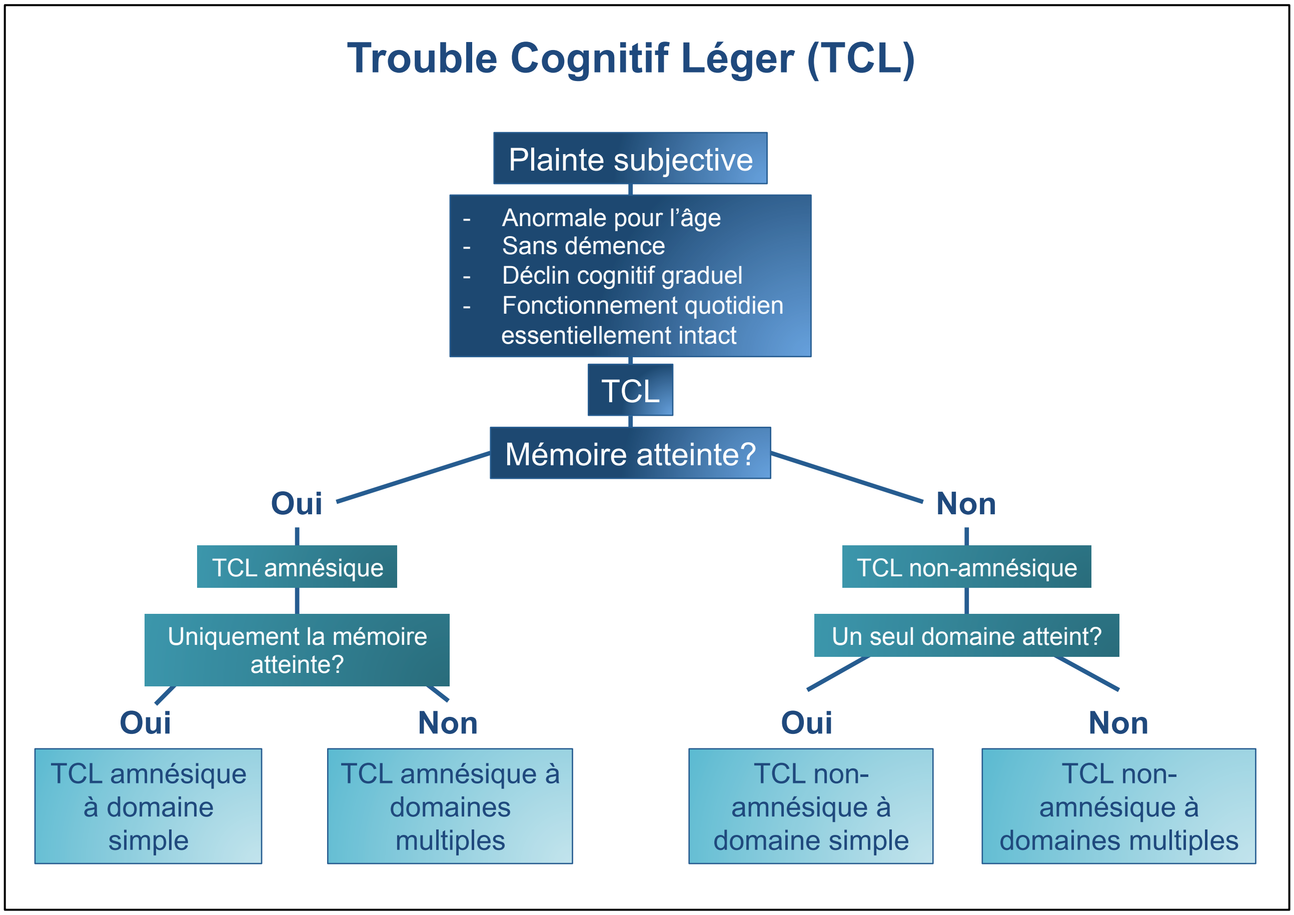

ANNEXE 4

Questionnaire de santé générale

Un questionnaire de santé générale sera administré aux participants afin d'avoir un aperçu de leur état de santé. Si ce dernier est considéré comme trop précaire, la personne ne pourra pas participer à l'étude (ex. cancer en traitement). Pour ce faire, une courte entrevue semi-structurée sera administrée aux participants lors de l'évaluation diagnostique (voir le tableau ci-dessous).

Problèmes physiques ou médicaux	Présence, non-traité ou non-contrôlé	Présence, traité ou contrôlé	Durée ou date d'apparition
Hypertension artérielle			
Hypotension artérielle			
Diabète (type I ou II)			
Hypercholestérolémie			
Hyperlipidémie			
Problèmes cardiaques			
Histoire AVC ou AIT			
Tabagisme			
Obésité			
Alcoolisme			
Traumatismes crâniens			
Infection neurologique			
Cancer			
Troubles de la vision			
Troubles de l'olfaction			
Troubles auditifs			
Troubles gustatifs			
Troubles du toucher			
Troubles du sommeil			
Problèmes d'incontinence			
Problèmes d'ordre sexuel			
Hyperthyroïdie ou hypothyroïdie			
Épilepsie			
Paralysie			

Migraines			
Arthrite ou rhumatismes			
Sensations de faiblesses ou étourdissements			
Fatigue ou baisse d'énergie			

Questions de santé générales posées aux participants

Batterie de tests pour l'évaluation diagnostique

Attention et mémoire de travail.

La double tâche de Baddeley (Baddeley et al., 1997) mesure la performance d'un individu dans deux types de conditions : lors de l'accomplissement d'une tâche seule, ou lorsque deux tâches doivent être accomplies en même temps. Dans ce cas-ci, le participant doit effectuer d'abord une tâche d'empan verbal (e.g. répéter des séquences de chiffres), puis une tâche de poursuite visuelle (e.g. dessiner des X dans des carrés imprimés sur une feuille dans un certain ordre), et il doit ensuite faire les deux tâches en même temps. Le score à cette tâche est la différence entre la performance lors de la condition simple et celle de la condition double. Le score total (U) se calcule de la manière suivante :

$$U = (1 – (Pm + Pt/2)) * 100$$

*Où **Pm** est la différence entre la performance en condition simple et celle en condition double pour la tâche de mémorisation; et **Pt** est la différence entre la performance en condition simple et celle en condition double pour la tâche de poursuite visuelle. Donc, plus le score en pourcentage est élevé, meilleure est la performance.*

Le Trail Making Test ou TMT (conditions 1 à 3) de la D-KEFS (Delis, Kaplan et Kramer, 2001) sera aussi utilisé. Pour les trois conditions, les feuilles comportent de nombreux chiffres et lettres dispersés. La condition 1 correspond à une tâche de balayage visuel simple (e.g. rayer les 3). La condition 2 est de relier une séquence de nombre par un trait (1, 2, 3, 4, etc.). La condition 3 consiste à relier une séquence de lettre par un trait (A, B, C, D, etc.). Le temps requis pour compléter chaque condition constitue le score à ce test.

Les Blocs de Corsi (Kessels et al., 2000) mesurent l'empan spatial. Sur une structure comprenant des cubes dispersés sur la surface, l'expérimentateur pointe une séquence de cubes. Le participant doit ensuite pointer correctement la séquence qu'il vient de voir, dans le même ordre, puis dans l'ordre inverse. Le nombre maximal de cubes correctement pointés constitue le score de l'empan spatial.

La tâche d'empan envers de la WAIS-IV (Weschler, 2008) mesure l'empan verbal à l'aide d'une séquence de chiffre que le participant doit répéter dans l'ordre inverse après l'avoir entendue de l'examinateur. Dans ce cas-ci, c'est le nombre maximal de chiffres énoncés correctement dans l'ordre inverse est le score de ce test.

Fonctions langagières.

Un test de fluidité verbale sera utilisé. D'abord, la première condition évaluée sera une version récemment normalisée pour la population québécoise de fluidité lexicale (e.g. nommer le plus de mots possible commençant par les lettres T, N et P, une minute par lettre) (St-Hilaire et al., 2016). Ensuite, la deuxième condition sera une condition de fluidité sémantique. La tâche normalisée pour la population québécoise sera employée (e.g. nommer le plus d'animaux possible en une minute) (St-Hilaire et al., 2016).

Le sous-test «dénomination de la Neuropsychological Assessment Battery permet de mesurer la dénomination (White et Stern, 2003). L'expérimentateur montre des images représentant un objet familier. Le sujet doit nommer correctement les objets illustrés sur l'image. Un point par bonne réponse est accordé lorsque le participant nomme correctement l'objet.

Mémoire épisodique.

Dans le «*Brief Visuospatial Memory Test – Revised*» (BVMT–R; Benton, 1992), 15 formes complexes sont présentées au participant et celui-ci doit ensuite choisir le dessin qu'il a vu parmi d'autres choix. La forme de reconnaissance a été choisie en raison des incapacités motrices liées à la MP. Pour la cotation, le nombre de bonnes réponses choisies est la variable d'intérêt.

Le test de Rappel Libre – Rappel Indicé à 16 items (RL/RI-16, Van der Linden, Coyette, Poitrenaud, Kalafat, Calicis, Wyns, et al., 2004) a été choisi pour évaluer la mémoire épisodique en modalité verbale. Ce test est une traduction en français du «*Free and Cued Seclective Reminding Test*» (Buschke, 1984; Grober, Buschke, Crystal, Bang, & Dresner, 1988). Il permet notamment de différencier les difficultés d'encodage, de reconnaissance et de mémoire sémantique. D'abord, une liste de 16 mots est apprise au participant. Ces mots sont écrits sur des cartons en groupe de quatre. Afin que le participant identifie chacun des mots, un indice sémantique est fourni. Un rappel indicé immédiat est fait pour chacune des quatre planches. Le participant dispose de trois essais par planche pour apprendre les mots. Ensuite,

une tâche d'interférence (i.e. compter à rebours pendant 20 secondes) est demandée au participant. Par après, le participant doit rappeler immédiatement le plus de mots possible en deux minutes, peut importe l'ordre. Pour chaque mot qui n'aurait pas été rappelé, l'indice sémantique est fournit à nouveau pour aider le participant à rappeler le mot. Au total, trois essais sont ainsi administrés pour rappeler le plus de mots possible. Après cela, un délai de 20 minutes est laissé, puis un autre essai de rappel différé libre et indicé est fait. Ensuite, une tâche de reconnaissance est administrée, dans laquelle les 16 mots initiaux, 16 distracteurs appartenant aux mêmes catégories sémantiques ainsi que 16 autres distracteurs qui n'ont pas de lien avec les mots initiaux sont tous montrés. Pour la cotation, chaque mot rappelé correctement vaut un point, que ce soit en rappel libre, indicé, ou en reconnaissance. Pour cette dernière, il est aussi important de noter les distracteurs nommés par le participant. Des normes québécoises sont disponibles pour ce test (Dion, Potvin, Belleville, Ferland, Renaud & al., 2015).

Fonctions visuospatiales.

Le test de Jugement d'orientation des lignes (Benton, 1994) est un test dans lequel deux lignes avec des angles différents sont présentées au participant. Ensuite, 11 lignes de référence dispersées en demi-cercle sur une feuille sont montrées. Le participant doit coupler correctement chacune des deux lignes à une des 11 lignes de référence. En tout, le test comprend 30 items. Le score total est le nombre de bonnes réponses choisies par le participant.

Le sous-test Casse-tête visuel de la WAIS-IV (Weschler, 2008) est un test dans lequel une série de figures complexes est montrée au participant. Pour chaque figure, le participant doit choisir trois formes simples parmi six qui permettent de compléter adéquatement la figure complexe. Pour avoir un point, le participant doit choisir correctement les trois formes à chacun des items (1 ou 0 par item, pour un maximum possible de 24).

Fonctions exécutives.

D'abord, un sous-test de la BADS a été choisi (Wilson et al., 1996). Pour la tâche modifiée des Six éléments, le participant doit faire six activités et doit avancer le plus possible en 10 minutes. Le but est donc d'observer ses capacités d'organisation et de planification. Le score à cette tâche se calcule en fonction du nombre de tâches complétées, moins le nombre de tâches qui contiennent une erreur. Le

temps requis et le nombre d'erreurs correspondent à un profil de points, se situant entre zéro et quatre (quatre correspondant à un score parfait).

Les deux dernières conditions (4 et 5) du TMT de la D-KEFS (Delis, Kaplan et Kramer, 2001) permettent d'évaluer respectivement les capacités d'alternance et la vitesse psychomotrice. Encore une fois, c'est le temps requis pour exécuter la tâche qui est la variable d'intérêt. La condition 4 mesure la capacité d'alternance car le sujet doit relier dans les lettres en ordre alphabétique et les chiffres en ordre croissant et en les alternant (e.g. A, 1, B, 2, C, 3, etc.). La dernière condition permet de contrôler pour les déficits moteurs, car il s'agit de tracer une ligne sur un pointillé le plus rapidement possible. Il sera possible de corriger les temps obtenus lors des conditions 1 à 4 avec le score de la condition 5. Le score de la condition 4 corrigé (en secondes) constituera la variable ici :

Score corrigé Condition 4 = Score Condition 4 – Score Condition 5

Théorie de l'esprit. La tâche de Faux Pas a été choisie (Stone et al., 1998), puisqu'elle a déjà été employée dans la littérature chez des patients MP pour montrer qu'il y avait un déficit de théorie de l'esprit (composante cognitive) chez ces patients (Kawamura et Koyama, 2007; Péron et al., 2009; Roca et al., 2010). La procédure est de lire 20 histoires courtes au participant et de vérifier s'il identifie correctement le faux pas qui a été commis pour 10 des histoires. Huit questions par histoire sont ensuite posées au participant pour s'assurer qu'il a bien compris. Les questions concernent la détection du faux pas, la compréhension du faux pas, la compréhension des intentions des protagonistes, la compréhension des croyances, la compréhension des émotions ainsi que la compréhension générale de l'histoire. Pour la cotation, un point par bonne réponse est donné au participant (8 questions fois 20 histoires : total maximum possible de 160 points). Toutefois, si les questions contrôles ne sont pas réussies, il n'est pas possible de donner les points au participant. De plus, différents ratios peuvent être calculés et semblent plus pertinents à l'analyse de la performance selon les auteurs du test (Stone et al., 1998). Ces ratios se calculent en pourcentage et sont expliqués en détails dans le manuel du test :

1 – Nombre réussi de questions contrôles, pour les histoires avec et sans faux pas.

2 – Score de détection correcte des faux pas.

3 – Score de compréhension des faux pas.

4 – Score de compréhension des intentions.

5 – Score de compréhension des croyances.

6 – Score de compréhension des émotions.

Cognition globale. Le «*Montreal Cognitive Assesment*» (MoCA, Nasreddine et al., 2005) est une mesure de dépistage qui inclut 11 sous-tests très brefs mesurant les fonctions visuo-spatiales, les fonctions exécutives, la dénomination, la mémoire, l'attention, le langage, l'abstraction ainsi que l'orientation dans le temps. Les réponses correctes, additionnées, permettent de calculer un score maximum possible et positif de 30.

ANNEXE 5 :

Échelle de fatigue remplie à la fin de chaque séance avec les participants

Goal Management Training

Numéro de dossier: _______________

Date: _______________

Évaluation de la fatigue

1 2 3 4 5 6 7 8 9 10

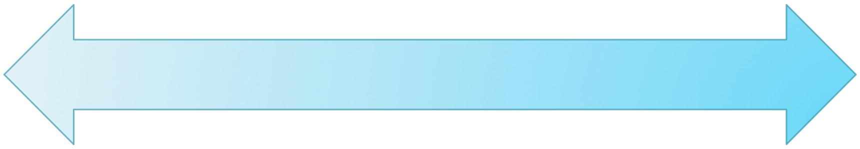

Aucune fatigue

Fatigue extrême

ANNEXE 6

Estimation des temps requis pour chacune des mesures d'évaluation diagnostique et d'efficacité du GMT

Tableau 6.1 - Évaluation diagnostique

Construit mesuré	Nom des épreuves / questionnaires	Temps requis (minutes)
**Attention et mémoire de travail (2)**	1 – Double tâche de Baddeley<br>2 – TMT conditions 1 - 3<br>3 – Blocs de Corsi<br>4 – Empan à l'envers de WAIS-IV	15-20<br>5-10<br>5<br>5
**Fonctions langagières (2)**	1 – Test de fluence verbale lexicale (TNP) et sémantique (animaux) de la D-KEFS<br>2 – Boston Naming Test	5<br>15
**Mémoire (2)**	1 – Brief Visuospatial Memory Test – Revised (BVMT–R)<br>2 – Rappel Libre – Rappel Indicé à 16 items	10-15<br>20-25
**Fonctions visuo-spatiales (2)**	1 – Jugement d'orientation des lignes (Benton)<br>2 – Casse-tête visuel de la WAIS-IV	15<br>15
**Fonctions exécutives (3)**	1 – Trail Making Test, conditions 4 – 5<br>2 – Tâche des six éléments de la BADS	5-10<br>10-15
**Cognition globale**	1 – MoCA	15
**Théorie de l'esprit**	1 – Tâche de Faux Pas	30-40

Temps total maximum : 3 h, répartis sur 3 séances de 60 à 75 minutes chacune maximum.

Tableau 6.2 - Efficacité du GMT : mesures primaires

Construit mesuré	Mesures	Temps requis (minutes)
**Tolérabilité / sécurité**	1 – Assiduité aux séances et aux activités proposées; changements dans la médication<br>2 – Évaluation du journal du participant (si mention de fatigue) et évaluation de la fatigue ressentie par le patient sur une échelle de type Likert avant et après chaque séance	5<br>5
**Fonctions exécutives**	1 – Dysexecutive (DEX) questionnaire<br>2 – Questionnaires du GMT (version participant) inclus dans le package<br>3 – Zoo Map Test de la BADS	10-15<br>10<br>10-15

Temps total maximum : 60 minutes.

Tableau 6.3 - Efficacité du GMT : Mesures secondaires

Construit mesuré	Nom des épreuves / questionnaires	Temps requis (minutes)
**Cognition**	Dementia Rating Scale – II	15-45
**AIVQ / Qualité de vie**	PDQ-39	20-25
**Fonctions exécutives du participant (PROCHE)**	1 – Dysexecutive (DEX) questionnaire, version aidant<br>2 – Questionnaires du GMT, version aidant	10-15<br>10
**Fardeau de l'aidant (PROCHE)**	Zarit Bruden Interview - 12	10-15
**Symptômes cognitifs et comportementaux du participant (PROCHE)**	Neuropsychiatric Inventory, versions aidant et clinicien	15

Temps total maximum: pour le participant, 1h; pour les proches, 55 minutes.

ANNEXE 7

Tableau 7.1 - Planification des tests pour l'étude pilote : participant 1 (deux mesures de niveau de base)

Date:	Sem 1	Sem 2	Sem 3	Sem 4	Sem 5	Sem 6	Sem 7	Sem 8	Sem 9	Sem 10	Sem 11	…	Sem 16
	Cons.	Éval DX	Éval DX	Éval Dx	Niv. Base (2X/sem)	GMT-1	GMT-2	GMT-3	GMT-4	GMT-5	Mesures Post	…	Suivi 1 mois
**Évaluation Diagnostique**													
MoCA		X											
Tâche double de Baddeley		X											
Tâche des six éléments		X											
Empan Envers		X											
Dénomination de la NAB		X											
Jugement orientation des lignes		X											
RL/RI-16			X										
TMT			X										
Bloc de Corsi			X										
BVMT-R			X										
Fluence verbale (lexicale et sémantique)			X										
Casse-tête visuel			X										
Tâche de Faux Pas				X									
DRS-2				X									
**Tolérabilité**													
Fatigabilité					X X	X	X	X	X	X	X		X
Changement de médication					X X	X	X	X	X	X	X		X
NPI (AIDANT)					X X						X		X
ZBI-12 (AIDANT)					X X						X		X
PDQ-39					X X						X		X
**Efficacité: participant**													
DEX					X X						X		X
Questionnaires du GMT					X X						X		X
Zoo Map Test					X X						X		X
**Efficacité: proche aidant**													
DEX					X X						X		X
Questionnaires du GMT					X X						X		X

Légende:
Cons. = Consentement
Éval. DX = Évaluation diagnostique
Niv. Base = Niveau de base
GMT 1 à 5 = Séances du GMT
Mesures post = post-GMT

Tableau 7.2 - Planification des tests pour l'étude pilote : participant 2 (trois mesures de niveau de base)

Date:	Sem 1	Sem 2	Sem 3	Sem 4	Sem 5	Sem 6	Sem 7	Sem 8	Sem 9	Sem 10	Sem 11	…	Sem 16
	Cons.	Éval DX	Éval DX	Éval Dx	Niv. Base (2X/sem)	GMT-1	GMT-2	GMT-3	GMT-4	GMT-5	Mesures Post	…	Suivi 1 mois
**Évaluation Diagnostique**													
MoCA		X											
Tâche double de Baddeley		X											
Tâche des six éléments		X											
Empan Envers		X											
Dénomination de la NAB		X											
Jugement orientation des lignes		X											
RL/RI-16			X										
TMT			X										
Bloc de Corsi			X										
BVMT-R			X										
Fluence verbale (lexicale et sémantique)			X										
Casse-tête visuel			X										
Tâche de Faux Pas				X									
DRS-2				X									
**Tolérabilité**													
Fatigabilité					X X	X	X	X	X	X	X		X
Changement de médication					X X	X	X	X	X	X	X		X
NPI (AIDANT)					X X						X		X
ZBI-12 (AIDANT)					X X						X		X
PDQ-39					X X						X		X
**Efficacité: participant**													
DEX					X X						X		X
Questionnaires du GMT					X X						X		X
Zoo Map Test					X X						X		X
**Efficacité: proche aidant**													
DEX					X X						X		X
Questionnaires du GMT					X X						X		X

Légende:
Cons. = Consentement
Éval. DX = Évaluation diagnostique
Niv. Base = Niveau de base
GMT 1 à 5 = Séances du GMT
Mesures post = post-GMT

Tableau 7.3 - Planification des tests pour l'étude randomisée – contrôle

Date:	Sem 1	Sem 2	Sem 3	Sem 4	Sem 5	Sem 6	Sem 7	Sem 8	Sem 9	Sem 10	Sem 11	…	Sem 16	Sem 24
	Cons.	Éval DX	Éval DX	Éval Dx	Niv. Base	INTERV 1	INTERV 2	INTERV 3	INTERV 4	INTERV 5	Mesures Post	…	Suivi 1 mois	Suivi 3 mois
**Évaluation Diagnostique**														
Questionnaire médical		x												
MoCA		x												
Tâche double de Baddeley		x												
Tâche des six éléments		x												
Empan Envers		x												
Dénomination de la NAB		x												
Jugement orientation des lignes		x												
RL/RI-16			x											
TMT			x											
Bloc de Corsi			x											
BVMT-R			x											
Fluence verbale (lexicale et sémantique)			x											
Casse-tête visuel			x											
DRS-2				x							x		x	x
**Tolérabilité**														
Fatigabilité					x	x	x	x	x	x	x		x	x
Changement de médication					x	x	x	x	x	x	x		x	x
NPI (AIDANT)					x			x			x		x	x
ZBI-12 (AIDANT)					x			x			x		x	x
PDQ-39					x			x			x		x	x
**Efficacité: participant**														
DEX					x			x			x		x	x
Questionnaires du GMT					x			x			x		x	x
Zoo Map Test					x						x		x	x
**Efficacité: proche aidant**														
DEX					x			x			x		x	x
Questionnaires du GMT					x			x			x		x	x

Légende:
Cons. = Consentement
Éval. DX = Évaluation diagnostique
Niv. Base = Niveau de base
INTERV = Intervention
Mesures post = post-intervention

ANNEXE 8

Adaptations du GMT pour patients MP-TCL, comparaison avec le GMT original

GMT original	GMT modifié et adapté pour patients MP-TCL
Total: neuf modules.	Total: cinq modules.
Durée moyenne de chaque module:<br>une heure trente.	Durée moyenne de chaque module:<br>une heure.
Exercices proposés lors des séances:<br>• Respiration et pleine conscience,<br>• Distribution de cartes (alternance de règle),<br>• Tâches complexes (gestion de plusieurs objectifs).	Exercices proposés lors des séances:<br>• Respiration et pleine conscience,<br>• Tâches complexes (gestion de plusieurs objectifs) *seulement lors des deux derniers modules*.
Exercices proposés entre les séances:<br>• Tableaux à remplir,<br>• Exercices de pleine conscience,<br>• Intégration des acquis dans les activités quotidiennes.	Exercices proposés entre les séances:<br>• Mêmes exercices, mais moins de tableaux, intégrés sous forme de discussion.
Répétition fréquente des notions théoriques (absence d'esprit et erreurs, pilote automatique, tableau mental, cycle des ARRÊTS!) et plusieurs résumés des notions par module.	Répétition moins fréquente et un seul résumé à la fin de chaque module.<br>Une pause de 5 minutes est proposée au milieu des séances au besoin.
Format idéal en petits groupes.	Format individuel, en présence du proche aidant.

ANNEXE 9

Plan provisoire pour les séances de psychoéducation du groupe contrôle actif

*** Chaque séance de psychoéducation est d'une durée approximative de 30 à 40 minutes, suivie d'une séance d'exercices de pleine conscience d'une durée de 20 à 30 minutes (exercices de respiration et de balayage corporel). Les participants n'auront pas d'exercices à faire entre les séances à proprement dit, à moins d'une initiative personnelle.*

<u>Séance 1 : Présentation générale de la MP</u>

- Système dopaminergique (altérations du)
- Neuroanatomie (structures atteintes)
- Symptômes moteurs, sous-types et progression des symptômes
- Types de traitements disponibles trucs et astuces pour gérer ces symptômes (discussion avec le proche)

<u>Séance 2 : Symptômes non-moteurs</u>

- Sommeil
    - Trouble du sommeil paradoxal
    - Insomnie / hypersomnie
- Troubles du système nerveux autonome
    - Odorat
    - Digestion
- Traitements disponibles, trucs et astuces pour gérer ces symptômes (discussion avec le patient et le proche)

<u>Séance 3 : Symptômes non-moteurs (symptômes psychologiques et comportementaux)</u>

- Dépression et anxiété
- Symptômes psychotiques

- Traitements disponibles, trucs et astuces pour gérer ces symptômes (discussion avec le patient et le proche)

Séance 4 : Symptômes non-moteurs (cognition 1 – les fonctions cognitives)

- Mémoire
- Attention
- Langage
- Fonctions visuospatiales
- Fonctions exécutives

Séance 5 : Symptômes non-moteurs (cognition 2 – cognition et MP)

- Profil des atteintes dans la MP
- MP-TCL
- Démence parkinsonienne
- Traitements disponibles, trucs et astuces pour gérer ces symptômes (discussion avec le proche)
