# Supplementary material for: Goal management training and psychoeducation / mindfulness for treatment of executive dysfunction in Parkinson’s disease: A feasibility pilot trial
Source: PLoS One. 2022 Feb 18;17(2):e0263108. doi: 10.1371/journal.pone.0263108 (PMC8856541; doi:10.1371/journal.pone.0263108)
Supplement: S2 File — (ZIP) [file pone.0263108.s003.zip › 2020-protocol-PD-MCI-AGR.pdf]

ClinicalTrials.gov PRS DRAFT Receipt (Working Version)
Last Update: 11/16/2020 08:27

ClinicalTrials.gov ID: NCT04636541

Study Identification

Unique Protocol ID: AGiguère-Rancourt

Brief Title: Goal Management Training for Parkinson Disease Mild Cognitive Impairment

Official Title: Goal Management Training Home-Based Approach for Cognitive Impairment in Parkinson's Disease: A Single-Blind Randomized Trial

Secondary IDs:

Study Status

Record Verification: November 2020

Overall Status: Completed

Study Start: April 30, 2018 [Actual]

Primary Completion: July 20, 2019 [Actual]

Study Completion: July 20, 2019 [Actual]

Sponsor/Collaborators

Sponsor: Laval University

Responsible Party: Sponsor

Collaborators:

Oversight

U.S. FDA-regulated Drug: No

U.S. FDA-regulated Device: No

U.S. FDA IND/IDE: No

Human Subjects Review: Board Status: Approved
Approval Number: 2017-2867
Board Name: Comité d'éthique de la recherche
Board Affiliation: CHU de Québec - Université Laval Regulatory
Phone: 418-525-4444
Email: ethiquedelarecherche@chudequebec.ca
Address:

10, de l'Espinay, édifice D, 7th floor Québec (Québec) G1L3L5

Data Monitoring: No

FDA Regulated Intervention: No

Study Description

Brief Summary: Mild cognitive impairment is experienced by approximately 30% of patients with Parkinson's disease (PD-MCI), often affecting executive functions. There is currently no pharmacological treatment available for PD-MCI and non-pharmacological treatments are still scarce. The aim of this study was to test preliminary efficacy/effectiveness of two home-based cognitive interventions adapted for patients with PD-MCI: Goal Management Training, adapted for PD-MCI (Adapted-GMT), and a psychoeducation program combined with mindfulness exercises. Twelve persons with PD-MCI with executive dysfunctions, as measured by extensive neuropsychological evaluation, were randomly assigned to one of two intervention groups. Both groups received five sessions each lasting 60-90 minutes for five weeks, in presence of the caregiver. Measures were collected at baseline, mid-point, at one-week, four-week and 12-week follow-ups. Primary outcomes were executive functions assessed by subjective (DEX questionnaire patient- and caregiver-rated) and objective (Zoo Map Test) measures. Secondary outcomes included quality of life (PDQ-39), global cognition (DRS-II), and neuropsychiatric symptoms (NPI-12). Safety data (fatigue, medication change and compliance) were also recorded. Repeated measures ANCOVAs were applied to outcomes. Both groups significantly ameliorated executive functions overtime as indicated by improvements in DEX-patient and DEX-caregiver scores. PDQ-39 scores decreased at the four-week follow-up in the Psychoeducation/Mindfulness group whereas they were maintained in the Adapted-GMT group. All other measures were maintained over time in both groups. Adapted-GMT and Psychoeducation/Mindfulness groups both improved executive functioning. This is one of the first studies to test home-based approaches, tailored to the participant's cognitive needs, and involving caregivers.

Detailed Description:

Conditions

Conditions: Parkinson Disease
Mild Cognitive Impairment

Keywords: Mild Cognitive Impairment
Parkinson Disease
Cognitive Intervention
Goal Management Training
Executive Functions

Study Design

Study Type: Interventional

Primary Purpose: Supportive Care

Study Phase: N/A

Interventional Study Model: Parallel Assignment
Comparison of two different cognitive intervention (two groups, randomized, single blinded)

Number of Arms: 2

Masking: Triple (Participant, Care Provider, Outcomes Assessor)
The research was a single blind randomized comparative study. After the screening evaluation, participants were randomly assigned to either group A or B, described below (block randomization, three blocks of four participants).

Allocation: Randomized

Enrollment: 12 [Actual]

Arms and Interventions

Arms	Assigned Interventions
Experimental: Goal Management Training<br>GMT modules were adapted for French-speaking patients with PD-MCI. Each session was reduced from nine 90-120-minute sessions (original GMT) to five 60-90-minute sessions, one session per week, in order to avoid fatigue. As for original GMT, participants were given exercises between sessions (mindfulness exercises and metacognitive reflections). In original-GMT, some information is repeated several times, but not in Adapted-GMT. Exercises demanding motor dexterity, such as card distribution, were removed. Adapted-GMT included information on PD-MCI and executive dysfunction (some psychoeducation). In addition, Adapted-GMT modules were administered individually with an iPad, as opposed to a power-point group presentation in original-GMT. A workbook was handed to participants, as in previous studies.	Behavioral: Goal Management Training<br>Goal Management Training® (GMT) has been developed to improve executive functions. It was validated in patients presenting executive dysfunction following many conditions: acquired traumatic brain injury, neurodevelopmental spina bifida, attention deficit and hyperactivity disorder (ADHD), subjective cognitive complaints and multiple sclerosis. GMT includes self-instruction strategies, self-monitoring exercises, cognitive training techniques, psychoeducation on cognitive processes, mindfulness exercises and assignments between sessions. It has been shown to increase patient awareness of deficits and improve cognitive control in goal-directed behaviors. The original GMT is a nine-week program administered to dysexecutive patients in 90-to-120-minute group sessions. Thus, it might be suitable for PD-MCI patients presenting with executive dysfunction.
Active Comparator: Psychoeducation sessions coupled mindfulness exercises<br>Five modules were designed as a discussion with patients and caregivers about various PD symptoms: module I-brain and motor symptoms; module II-autonomic symptoms; module III- psychological symptoms; module IV-brain and cognition; and module V-cognitive impairments in PD. Patients were handed the information book about the five modules at the beginning of the study. The objective was to improve their understanding of their condition and to discuss other components that could affect their cognitive abilities. After the 40-60-minute informative part, mindfulness exercises were offered for 20-30 minutes per session. Participants were not invited to practice exercises between sessions, but 3/6 participants reported they did.	Behavioral: Psychoeducation<br>See the Arm section for full details. For a justification of how we designed this intervention: Many clinical guidelines include general recommendations about giving information to PD patients and family so they can take part into decision process. However, few standardized psychoeducation interventions are available, and they don't include information on PD cognitive decline. Some studies investigated Mindfulness Based Stress Reduction (MBSR) and other related mindfulness interventions in PD patients. In this approach, formal meditative exercises are included to develop non-judgmental attention to experiences in the present moment. In elderly patients with MCI unrelated to PD, mindfulness interventions show positive effects on cognitive functioning, including attention, executive functioning and memory (Gard et al., 2014). Therefore, non-pharmacological interventions for PD-MCI including both education on cognitive symptoms, as well as mindfulness exercises, are promising.

Outcome Measures

Primary Outcome Measure:

1. Raw score Change from baseline DEX (self rated) to 3 weeks after beginning of intervention
   Questionnaire on subjective executive functions

   [Time Frame: 3 weeks after beginning of intervention (mid-point)]
2. Raw score Change from baseline DEX (self rated) to 1 week post test
   Questionnaire on subjective executive functions

[Time Frame: 1 week post-test]

3. Raw score Change from baseline DEX (self rated) to 4 weeks post test
   Questionnaire on subjective executive functions

   [Time Frame: 4 weeks post-test]

4. Raw score Change from baseline DEX (self rated) to 12 weeks post test
   Questionnaire on subjective executive functions

   [Time Frame: 12 weeks post-test]

5. Raw score Change from baseline DEX (caregiver rated) to 3 weeks after the beginning of intervention
   Questionnaire on subjective executive functions (caregiver rates the executive functions of the participant

   [Time Frame: 3 weeks after beginning of intervention (mid-point)]

6. Raw score Change from baseline DEX (caregiver rated) to 1 week post test
   Questionnaire on subjective executive functions (caregiver rates the executive functions of the participant

   [Time Frame: 1 week post-test]

7. Raw score Change from baseline DEX (caregiver rated) to 4 weeks post test
   Questionnaire on subjective executive functions (caregiver rates the executive functions of the participant

   [Time Frame: 4 weeks post-test]

8. Raw score Change from baseline DEX (caregiver rated) to 12 weeks post test
   Questionnaire on subjective executive functions (caregiver rates the executive functions of the participant

   [Time Frame: 12 weeks post-test]

9. Raw score Change from baseline Zoo Map Test to 1 week post test
   Neuropsychological test assessing planification and organisation

   [Time Frame: 1 week post-test]

10. Raw score Change from baseline Zoo Map Test to 4 weeks post test
    Neuropsychological test assessing planification and organisation

    [Time Frame: 4 weeks post-test]

11. Raw score Change from baseline Zoo Map Test to 12 weeks post test
    Neuropsychological test assessing planification and organisation

    [Time Frame: 12 weeks post-test]

Secondary Outcome Measure:

12. Raw score Change from baseline Parkinson Disease Questionnaire (39 items; PDQ-39) to 3 weeks after the beginning of intervention
    Self rated questionnaire on quality of life with symptoms of Parkinson Disease

    [Time Frame: 3 weeks after beginning of intervention (mid-point of intervention)]

13. Raw score Change from baseline PDQ-39 to 1 week post-test
    Self rated questionnaire on quality of life with symptoms of Parkinson Disease

    [Time Frame: 1 week post-test]

14. Raw score Change from baseline PDQ-39 to 4 weeks post-test
    Self rated questionnaire on quality of life with symptoms of Parkinson Disease

    [Time Frame: 4 weeks post-test]

15. Raw score Change from baseline PDQ-39 to 12 weeks post-test
    Self rated questionnaire on quality of life with symptoms of Parkinson Disease

    [Time Frame: 12 weeks post-test]

16. Mean Change from baseline Dementia Rating Scale, 2nd edition (DRS-II) to 1 week post-test
    A brief neuropsychological instrument designed to assess general cognitive functioning

[Time Frame: 1 week post-test]

17. Mean Change from baseline Dementia Rating Scale, 2nd edition (DRS-II) to 4 weeks post-test
    A brief neuropsychological instrument designed to assess general cognitive functioning

    [Time Frame: 4 weeks post-test]
18. Mean Change from baseline Dementia Rating Scale, 2nd edition (DRS-II) to 12 weeks post-test
    A brief neuropsychological instrument designed to assess general cognitive functioning

    [Time Frame: 12 weeks post-test]
19. Raw score Change from baseline Zarit Burden Interview (12 items) to 3 weeks after the beginning of intervention
    A 12-item questionnaire assessing the feeling of burden of the caregiver

    [Time Frame: 3 weeks after the beginning of intervention (mid-point)]
20. Raw score Change from baseline Zarit Burden Interview (12 items) to 1 week post-test
    A 12-item questionnaire assessing the feeling of burden of the caregiver

    [Time Frame: 1 week post-test]
21. Raw score Change from baseline Zarit Burden Interview (12 items) to 4 weeks post-test
    A 12-item questionnaire assessing the feeling of burden of the caregiver

    [Time Frame: 4 weeks post-test]
22. Raw score Change from baseline Zarit Burden Interview (12 items) to 12 week post-test
    A 12-item questionnaire assessing the feeling of burden of the caregiver

    [Time Frame: Baseline, mid-point of intervention, 1 week post-test, 4 weeks post-test and 12 weeks post-test]
23. Raw score Change from baseline Neuropsychiatric Inventory, 12 items to 3 weeks after the beginning of intervention (mid-point)
    assessment of twelve neuropsychiatric symptoms usually found in dementia

    [Time Frame: 3 weeks after the beginning of intervention (mid-point)]
24. Raw score Change from baseline Neuropsychiatric Inventory, 12 items to 1 week post-test
    assessment of twelve neuropsychiatric symptoms usually found in dementia

    [Time Frame: 1 week post-test]
25. Raw score Change from baseline Neuropsychiatric Inventory, 12 items to 4 weeks post-test
    assessment of twelve neuropsychiatric symptoms usually found in dementia

    [Time Frame: 4 week post-test]
26. Raw score Change from baseline Neuropsychiatric Inventory, 12 items to 12 weeks post-test
    assessment of twelve neuropsychiatric symptoms usually found in dementia

    [Time Frame: 12 week post-test]
27. Raw score Change from baseline Apathy Evaluation Scale (AES) to 3 weeks after the beginning of intervention (mid-point)
    An 18-item questionnaire assessing different aspects of apathy (cognitive, behavioral and emotional).

    [Time Frame: 3 weeks after the beginning of intervention (mid-point)]
28. Raw score Change from baseline Apathy Evaluation Scale (AES) to 1 week post-test
    An 18-item questionnaire assessing different aspects of apathy (cognitive, behavioral and emotional).

    [Time Frame: 1 week post-test]
29. Raw score Change from baseline Apathy Evaluation Scale (AES) to 4 weeks post-test
    An 18-item questionnaire assessing different aspects of apathy (cognitive, behavioral and emotional).

    [Time Frame: 4 weeks post-test]
30. Raw score Change from baseline Apathy Evaluation Scale (AES) to 12 weeks post-test
    An 18-item questionnaire assessing different aspects of apathy (cognitive, behavioral and emotional).

[Time Frame: 12 weeks post-test]

Eligibility

Minimum Age: 50 Years

Maximum Age: 80 Years

Sex: All

Gender Based: No

Accepts Healthy Volunteers: No

Criteria: Inclusion Criteria:

1. PD diagnosis from the United Kingdom Research Brain Bank diagnostic criteria for PD (Hughes et al., 1992);
2. PD-MCI diagnosis from the Movement Disorder Society Task Force diagnostic criteria. Single and multiple-domain MCI were both included, only if executive functions were significantly impaired (-1 standard deviation on executive function tests according to age and education-adjusted norms);
3. Montreal Cognitive Assessment scores between 21 and 27;
4. Anti-Parkinson medication stable (at screening) since at least two months;
5. All other medications, including psychotropics, stable for at least three months.

Exclusion Criteria:

1. Participants with PD and dementia diagnosis
2. Patients with other neurological or psychiatric disorders.

Contacts/Locations

Central Contact Person: Ariane Giguère-Rancourt
Telephone: 418-564-7827
Email: ariane.giguere-rancourt.1@ulaval.ca

Central Contact Backup: Martine Simard
Telephone: 418-656-2131 Ext. 412193
Email: martine.simard@psy.ulaval.ca

Study Officials: Martine Simard
Study Director
Professor at Laval School of psychology

Locations: **Canada**
School of Psychology
Québec, Canada, G1V0A6
Contact: Ariane Giguère-Rancourt 418-564-7827 ariane.giguere-rancourt.1@ulaval.ca

School of Psychology
Québec, Canada, G1V0A6
Contact: Ariane Giguère-Rancourt 418-564-7827 ariane.giguere-rancourt.1@ulaval.ca

IPDSharing

Plan to Share IPD: No

References

Citations:

Clare L, Teale JC, Toms G, Kudlicka A, Evans I, Abrahams S, Goldstein LH, Hindle JV, Ho AK, Jahanshahi M, Langdon D, Morris R, Snowden JS, Davies R, Markova I, Busse M, Thompson-Coon J. Cognitive rehabilitation, self-management, psychotherapeutic and caregiver support interventions in progressive neurodegenerative conditions: A scoping review. NeuroRehabilitation. 2018;43(4):443-471. doi: 10.3233/NRE-172353. PubMed 30412509

[Study Results] Couture M, Giguère-Rancourt A, Simard M. The impact of cognitive interventions on cognitive symptoms in idiopathic Parkinson's disease: a systematic review. Neuropsychol Dev Cogn B Aging Neuropsychol Cogn. 2019 Sep;26(5):637-659. doi: 10.1080/13825585.2018.1513450. Epub 2018 Sep 17. PubMed 30221586

Cummings JL. The Neuropsychiatric Inventory: assessing psychopathology in dementia patients. Neurology. 1997 May;48(5 Suppl 6):S10-6. Review. PubMed 9153155

Dubois B, Burn D, Goetz C, Aarsland D, Brown RG, Broe GA, Dickson D, Duyckaerts C, Cummings J, Gauthier S, Korczyn A, Lees A, Levy R, Litvan I, Mizuno Y, McKeith IG, Olanow CW, Poewe W, Sampaio C, Tolosa E, Emre M. Diagnostic procedures for Parkinson's disease dementia: recommendations from the movement disorder society task force. Mov Disord. 2007 Dec;22(16):2314-24. Review. PubMed 18098298

[Study Results] Giguère-Rancourt A, Plourde M, Doiron M, Langlois M, Dupré N, Simard M. Goal management training ® home-based approach for mild cognitive impairment in Parkinson's disease: a multiple baseline case report. Neurocase. 2018 Oct - Dec;24(5-6):276-286. doi: 10.1080/13554794.2019.1583345. Epub 2019 Mar 1. PubMed 30821637

Goldman JG, Vernaleo BA, Camicioli R, Dahodwala N, Dobkin RD, Ellis T, Galvin JE, Marras C, Edwards J, Fields J, Golden R, Karlawish J, Levin B, Shulman L, Smith G, Tangney C, Thomas CA, Tröster AI, Uc EY, Coyan N, Ellman C, Ellman M, Hoffman C, Hoffman S, Simmonds D. Cognitive impairment in Parkinson's disease: a report from a multidisciplinary symposium on unmet needs and future directions to maintain cognitive health. NPJ Parkinsons Dis. 2018 Jun 26;4:19. doi: 10.1038/s41531-018-0055-3. eCollection 2018. Review. PubMed 29951580

Grimes D, Gordon J, Snelgrove B, Lim-Carter I, Fon E, Martin W, Wieler M, Suchowersky O, Rajput A, Lafontaine AL, Stoessl J, Moro E, Schoffer K, Miyasaki J, Hobson D, Mahmoudi M, Fox S, Postuma R, Kumar H, Jog M; Canadian Nourological Sciences Federation. Canadian Guidelines on Parkinson's Disease. Can J Neurol Sci. 2012 Jul;39(4 Suppl 4):S1-30. PubMed 23126020

Hindle JV, Watermeyer TJ, Roberts J, Brand A, Hoare Z, Martyr A, Clare L. Goal-orientated cognitive rehabilitation for dementias associated with Parkinson's disease-A pilot randomised controlled trial. Int J Geriatr Psychiatry. 2018 May;33(5):718-728. doi: 10.1002/gps.4845. Epub 2018 Jan 4. PubMed 29314218

Hughes AJ, Daniel SE, Kilford L, Lees AJ. Accuracy of clinical diagnosis of idiopathic Parkinson's disease: a clinico-pathological study of 100 cases. J Neurol Neurosurg Psychiatry. 1992 Mar;55(3):181-4. PubMed 1564476

Jenkinson C, Peto V, Fitzpatrick R, Greenhall R, Hyman N. Self-reported functioning and well-being in patients with Parkinson's disease: comparison of the short-form health survey (SF-36) and the Parkinson's Disease Questionnaire (PDQ-39). Age Ageing. 1995 Nov;24(6):505-9. PubMed 8588541

Litvan I, Goldman JG, Tröster AI, Schmand BA, Weintraub D, Petersen RC, Mollenhauer B, Adler CH, Marder K, Williams-Gray CH, Aarsland D, Kulisevsky J, Rodriguez-Oroz MC, Burn DJ, Barker RA, Emre M. Diagnostic criteria for mild cognitive impairment in Parkinson's disease: Movement Disorder Society Task Force guidelines. Mov Disord. 2012 Mar;27(3):349-56. doi: 10.1002/mds.24893. Epub 2012 Jan 24. Review. PubMed 22275317

Macht M, Gerlich C, Ellgring H, Schradi M, Rusiñol AB, Crespo M, Prats A, Viemerö V, Lankinen A, Bitti PE, Candini L, Spliethoff-Kamminga N, de Vreugd J, Simons G, Pasqualini MS, Thompson SB, Taba P, Krikmann U, Kanarik E. Patient education in Parkinson's disease: Formative evaluation of a standardized programme in seven European countries. Patient Educ Couns. 2007 Feb;65(2):245-52. Epub 2006 Sep 11. PubMed 16965885

Matteau E, Dupré N, Langlois M, Provencher P, Simard M. Clinical validity of the Mattis Dementia Rating Scale-2 in Parkinson disease with MCI and dementia. J Geriatr Psychiatry Neurol. 2012 Jun;25(2):100-6. doi: 10.1177/0891988712445086. PubMed 22689702

Roy MA, Doiron M, Talon-Croteau J, Dupré N, Simard M. Effects of Antiparkinson Medication on Cognition in Parkinson's Disease: A Systematic Review. Can J Neurol Sci. 2018 Jul;45(4):375-404. doi: 10.1017/cjn.2018.21. Epub 2018 May 11. PubMed 29747716

Stamenova V, Levine B. Effectiveness of goal management training® in improving executive functions: A meta-analysis. Neuropsychol Rehabil. 2019 Dec;29(10):1569-1599. doi: 10.1080/09602011.2018.1438294. Epub 2018 Mar 14. Review. PubMed 29540124

Vlagsma TT, Koerts J, Fasotti L, Tucha O, van Laar T, Dijkstra H, Spikman JM. Parkinson's patients' executive profile and goals they set for improvement: Why is cognitive rehabilitation not common practice? Neuropsychol Rehabil. 2016;26(2):216-35. doi: 10.1080/09602011.2015.1013138. Epub 2015 Feb 19. PubMed 25693688

Bora E, Walterfang M, Velakoulis D. Theory of mind in Parkinson's disease: A meta-analysis. Behav Brain Res. 2015 Oct 1;292:515-20. doi: 10.1016/j.bbr.2015.07.012. Epub 2015 Jul 9. Review. PubMed 26166188

Bédard M, Molloy DW, Squire L, Dubois S, Lever JA, O'Donnell M. The Zarit Burden Interview: a new short version and screening version. Gerontologist. 2001 Oct;41(5):652-7. PubMed 11574710

Marin RS, Biedrzycki RC, Firinciogullari S. Reliability and validity of the Apathy Evaluation Scale. Psychiatry Res. 1991 Aug;38(2):143-62. PubMed 1754629

Hiseman JP, Fackrell R. Caregiver Burden and the Nonmotor Symptoms of Parkinson's Disease. Int Rev Neurobiol. 2017;133:479-497. doi: 10.1016/bs.irn.2017.05.035. Epub 2017 Jul 21. Review. PubMed 28802929

Links:

Available IPD/Information:
