# Supplementary material for: Goal management training and psychoeducation / mindfulness for treatment of executive dysfunction in Parkinson’s disease: A feasibility pilot trial
Source: PLoS One. 2022 Feb 18;17(2):e0263108. doi: 10.1371/journal.pone.0263108 (PMC8856541; doi:10.1371/journal.pone.0263108)
Supplement: S2 File — (ZIP) [file pone.0263108.s003.zip › 2017-2867%20-%20Renouvellement%20annuel%20%28F9%20-%2035770%29%20%282019-02-04%29.pdf]

CHU de Québec Université Laval

Le 4 février 2019

Nicolas Dupré
a/s de Ariane Giguère-Rancourt
Neurosciences
Hôpital de l'Enfant-Jésus
CHU de Québec-Université Laval

Objet : Projet 2017-2867 / Renouvellement annuel F9 - 35770
«Goal Management Training» : Tolérabilité et efficacité d'un programme d'entraînement des fonctions exécutives chez des patients atteints de la maladie de Parkinson idiopathique
argi001

(6.F février 2019-C) /mml

Docteur Dupré,

Les membres du Comité d'éthique de la recherche du CHU de Québec-Université Laval ont pris connaissance de vos documents concernant le projet en titre en comité délégué le 1er février 2019.

- Renouvellement annuel - F9 - 35770
  - Formulaire DSP autorisé (2017-07).pdf

Décision du CÉR : Après évaluation et discussion, le Comité renouvelle l'approbation éthique du projet en titre pour une période de un an, **du 4 février 2019 au 4 février 2020**.

De plus, le Comité invite le chercheur à transmettre sa demande d'accès aux dossiers des usagers au DSP avec l'approbation de son renouvellement éthique.

À noter :

- Le Comité d'éthique de la recherche du CHU de Québec-Université Laval (numéros FWA00000329 et IRB00001242) est désigné par le gouvernement du Québec (MSSS) et adhère aux directives publiées dans l'*ÉPTC 2 - Énoncé de politique des trois conseils : Éthique de la recherche avec des êtres humains (2014)*, conformément au *Plan d'action ministériel en éthique de la recherche et en intégrité scientifique (MSSS1998);*
- Le Comité adhère aux exigences édictées pour les Comités d'éthique de la recherche selon la Partie C, Titre 5 du *Règlement sur les aliments et drogues (C.R.C. ch.870)* et agit en conformité avec les standards du *United States Code of Federal Regulations* encadrant la recherche impliquant des sujets humains;
- Le Comité fonctionne de manière compatible avec les standards internationaux en appliquant notamment la ligne directrice de l'ICH adoptée par Santé Canada: *Les bonnes pratiques cliniques : directives consolidées.*

Les membres seront informés de cette décision lors d'une prochaine réunion plénière.

Je vous prie d'agréer, Docteur Dupré, l'expression de mes sentiments les meilleurs.

Renée Destrempe

Renée Destrempe
Agente de planification, de programmation et de recherche
Bureau de l'éthique
CHU de Québec-Université Laval
ethiquedelarecherche@chudequebec.ca
