# Supplementary material for: Goal management training and psychoeducation / mindfulness for treatment of executive dysfunction in Parkinson’s disease: A feasibility pilot trial
Source: PLoS One. 2022 Feb 18;17(2):e0263108. doi: 10.1371/journal.pone.0263108 (PMC8856541; doi:10.1371/journal.pone.0263108)
Supplement: Attachment — Submitted filename: Response to reviewers.pdf [file pone.0263108.s004.pdf]

UNIVERSITÉ
LAVAL

Quebec City, October 18th, 2021.

Walid Kamal Abdelbasset, Ph.D.
Academic Editor
PLOS ONE
**RE:** Submission of the revised manuscript PONE-D-21-08484

Dear Dr Abdelbasset,

I am pleased to submit the revised manuscript entitled '*Goal Management Training and Psychoeducation for Treatment of Executive Dysfunction in Parkinson's disease: A Feasibility Pilot Trial'* for publication in PLOS One.

Again, we thank you and the reviewers for all your efforts, as well as for the pertinent comments. As you will see, we have made the requested changes in the revised manuscript. You will find both marked and unmarked versions of the manuscript.

We added a non-institutional e-mail address for the database in the author section: ariane.grancourt@gmail.com.

We sincerely hope that this revised version will be acceptable for publication in your journal. Should you have any questions, please do not hesitate to contact me; I will answer your queries with pleasure.

Best regards,

Ariane GR

Ariane Giguère-Rancourt, Ph.D.
School of Psychology
Université Laval, Pavillon Félix-Antoine Savard
2325 Allée des Bibliothèques
Québec (Canada) G1V 0A6
ariane.giguere-rancourt.1@ulaval.ca
