# Supplementary material for: Goal management training and psychoeducation / mindfulness for treatment of executive dysfunction in Parkinson’s disease: A feasibility pilot trial
Source: PLoS One. 2022 Feb 18;17(2):e0263108. doi: 10.1371/journal.pone.0263108 (PMC8856541; doi:10.1371/journal.pone.0263108)
Supplement: Attachment — Submitted filename: Response to reviewers dec 2021.pdf [file pone.0263108.s005.pdf]

UNIVERSITÉ
LAVAL

Quebec City, December 21st, 2021.

Walid Kamal Abdelbasset, Ph.D.
Academic Editor
PLOS ONE
**RE:** Submission of the revised manuscript PONE-D-21-08484

Dear Dr Abdelbasset,

I am pleased to submit the revised manuscript entitled '*Goal Management Training and Psychoeducation for Treatment of Executive Dysfunction in Parkinson's disease: A Feasibility Pilot Trial*' for publication in PLOS One.

Again, we thank you and the reviewers for all your efforts, as well as for the pertinent comments. As you will see, we have made the requested changes in the revised manuscript. You will find both marked and unmarked versions of the manuscript.

We sincerely hope that this revised version will be acceptable for publication in your journal. Should you have any questions, please do not hesitate to contact me; I will answer your queries with pleasure.

Best regards,

Ariane GR

Ariane Giguère-Rancourt, Ph.D.
School of Psychology
Université Laval, Pavillon Félix-Antoine Savard
2325 Allée des Bibliothèques
Québec (Canada) G1V 0A6
ariane.giguere-rancourt.1@ulaval.ca

# Referee(s)' Comments to Author:

## Reviewer #1: Reviewer comments

Thank you for giving me this opportunity to review this article.

Abstract:

1. Summarize the abstract (follow the abstract guidelines). **RESPONSE: the abstract was summarized in 300 words.**
2. Include the study duration and eligibility criteria of study participants. **RESPONSE: Added. Please see the method section of the abstract.**
3. Mention the reports with 95%CI (Upper – lower limit) for all the variables. **RESPONSE: Added.**
4. The conclusion should be more concise and drawn on the basis of study reports. **RESPONSE: The conclusion was summarized.**

Manuscript

1. Summarize the introductory part. **RESPONSE: The introduction was briefly summarized.**
2. How come this trial is differing from reference number 9? – please justify. **RESPONSE: Couture et al. (2019) is a systematic review of all cognitive intervention RCTs for PD patients, whereas the present study is a feasibility pilot trial assessing the effects of two interventions that were never tested before with PD-MCI patients.**
3. Please describe about Psych – mind treatment and its benefits. **RESPONSE: Please see the description provided at page 8-9.**
4. The authors fail to find and report the research gap in this session. **RESPONSE: Please see the last paragraph of the introduction, page 6, which tries to explain the research gap.**
5. Include the clinical significance of this trial over clinicians, patients and researchers. **RESPONSE: Please see the end of introduction at pages 5 and 6.**
6. Present the manuscript as per CONSORT guidelines. **RESPONSE: You will find at the end of the present letter the CONSORT Table.**
7. Include the study setting and study duration. **RESPONSE: Study setting, and duration is described at page 8 and in figure 2.**
8. Include the reliability and validity of all the outcome measures used in the study. **RESPONSE: Please see page 14-15 for reliability and validity of outcome measures.**
9. Include the detail description of intervention and control group. **RESPONSE: There was no control group, since this is a single blind randomized between group comparative study. Both groups are described in detail in the method section, please see page 8 and 9.**
10. Include the method of sample size calculation with suitable reference. **RESPONSE: The power analysis was withdrawn at the first round of revision, please see your comment 17 of the first letter: « *What is the need of doing sample size calculation as it is a feasibility pilot trial?* ». The power calculation was therefore deleted.**
11. Mention about the demographic details of the participants in the results section. **RESPONSE: Please see table 1 at page 11-12 and the first paragraph of the result section, page 15.**
12. In the results section, please discuss about the treatment compliance rate, adverse effects and the number of dropouts. **RESPONSE: Please see the feasibility results provided at pages 15, 16 and 17, as well as the discussion, page 21.**
13. Mention the reports with 95%CI (Upper – lower limit) for all the variables. **RESPONSE: Added in the text, please see the result section, pages 19-20.**
14. Report the effect size and MCID values of all the primary and secondary variables. **RESPONSE: Added in Table 2.**
15. Summarize the discussion part and include the mechanism of interventions on different variables with recent references. **RESPONSE: We basically restructured and re-written the entire text in the**

**Introduction and Discussion sections and discussed potential mechanism of interventions (please see page 26), as well as discussion with recent references (pages 23-24).**

16. The conclusion should be more concise and drawn on the basis of study reports. **RESPONSE: Please see page 29 for the revised conclusion.**

# CONSORT 2010 checklist of information to include when reporting a randomised trial*

| Section/Topic | Item No | Checklist item | Reported on page |
|---|---|---|---|
| **Title and abstract** | | | |
| | 1a | Identification as a randomised trial in the title | 1 |
| | 1b | Structured summary of trial design, methods, results, and conclusions (for specific guidance see CONSORT for abstracts) | 2 |
| **Introduction** | | | |
| Background and objectives | 2a | Scientific background and explanation of rationale | 3-6 |
| | 2b | Specific objectives or hypotheses | 6 |
| **Methods** | | | |
| Trial design | 3a | Description of trial design (such as parallel, factorial) including allocation ratio | 6-14 |
| | 3b | Important changes to methods after trial commencement (such as eligibility criteria), with reasons | N-A |
| Participants | 4a | Eligibility criteria for participants | 7 |
| | 4b | Settings and locations where the data were collected | 6-7 |
| Interventions | 5 | The interventions for each group with sufficient details to allow replication, including how and when they were actually administered | 8-9 |
| Outcomes | 6a | Completely defined pre-specified primary and secondary outcome measures, including how and when they were assessed | 9-14 |
| | 6b | Any changes to trial outcomes after the trial commenced, with reasons | N-A |
| Sample size | 7a | How sample size was determined | 14 |
| | 7b | When applicable, explanation of any interim analyses and stopping guidelines | N-A |
| Randomisation: | | | |
| Sequence generation | 8a | Method used to generate the random allocation sequence | 8 |
| | 8b | Type of randomisation; details of any restriction (such as blocking and block size) | 8 |

| | | | |
|---|---|---|---|
| Allocation concealment mechanism | 9 | Mechanism used to implement the random allocation sequence (such as sequentially numbered containers), describing any steps taken to conceal the sequence until interventions were assigned | 8 |
| Implementation | 10 | Who generated the random allocation sequence, who enrolled participants, and who assigned participants to interventions | 8 |
| Blinding | 11a | If done, who was blinded after assignment to interventions (for example, participants, care providers, those assessing outcomes) and how | 8 |
| | 11b | If relevant, description of the similarity of interventions | 8-9 |
| Statistical methods | 12a | Statistical methods used to compare groups for primary and secondary outcomes | 14 |
| | 12b | Methods for additional analyses, such as subgroup analyses and adjusted analyses | N-A |
| | | **Results** | |
| Participant flow (a diagram is strongly recommended) | 13a | For each group, the numbers of participants who were randomly assigned, received intended treatment, and were analysed for the primary outcome | 14 + figure 1-2 |
| | 13b | For each group, losses and exclusions after randomisation, together with reasons | 14 + figure 1 |
| Recruitment | 14a | Dates defining the periods of recruitment and follow-up | 7-8 + figure 2 |
| | 14b | Why the trial ended or was stopped | N-A |
| Baseline data | 15 | A table showing baseline demographic and clinical characteristics for each group | Table 1 - p.11 |
| Numbers analysed | 16 | For each group, number of participants (denominator) included in each analysis and whether the analysis was by original assigned groups | Table 2 – p. 18 |
| Outcomes and estimation | 17a | For each primary and secondary outcome, results for each group, and the estimated effect size and its precision (such as 95% confidence interval) | Table 2 - p. 18 |
| | 17b | For binary outcomes, presentation of both absolute and relative effect sizes is recommended | N-A |
| Ancillary analyses | 18 | Results of any other analyses performed, including subgroup analyses and adjusted analyses, distinguishing pre-specified from exploratory | 14-20 |
| Harms | 19 | All-important harms or unintended effects in each group (for specific guidance see CONSORT for harms) | 14-15 |
| | | **Discussion** | |
| Limitations | 20 | Trial limitations, addressing sources of potential bias, imprecision, and, if relevant, multiplicity of analyses | 28-29 |

| | | | |
|---|---|---|---|
| Generalisability | 21 | Generalisability (external validity, applicability) of the trial findings | 29 |
| Interpretation | 22 | Interpretation consistent with results, balancing benefits and harms, and considering other relevant evidence | 20-27 |
| **Other information** | | | |
| Registration | 23 | Registration number and name of trial registry | 7 |
| Protocol | 24 | Where the full trial protocol can be accessed, if available | 7 |
| Funding | 25 | Sources of funding and other support (such as supply of drugs), role of funders | 1 |

N-A: Non-available

* We strongly recommend reading this statement in conjunction with the CONSORT 2010 Explanation and Elaboration for important clarifications on all the items. If relevant, we also recommend reading CONSORT extensions for cluster randomised trials, non-inferiority and equivalence trials, non-pharmacological treatments, herbal interventions, and pragmatic trials. Additional extensions are forthcoming: for those and for up to date references relevant to this checklist, see www.consort-statement.org.
